# Expression of circadian regulatory genes is dysregulated by increased cytokine production in mice subjected to concomitant intestinal injury and parenteral nutrition

**DOI:** 10.1371/journal.pone.0290385

**Published:** 2023-08-30

**Authors:** Colin T. Shearn, Aimee L. Anderson, Michael W. Devereaux, Karim C. El Kasmi, David J. Orlicky, Ronald J. Sokol

**Affiliations:** 1 Department of Pediatrics, Section of Pediatric Gastroenterology, Hepatology and Nutrition, University of Colorado Anschutz Medical Campus, School of Medicine, Aurora, CO, United States of America; 2 Digestive Health Institute, University of Colorado Anschutz Medical Campus, School of Medicine, Aurora, CO, United States of America; 3 Department of Pathology, University of Colorado Anschutz Medical Campus, School of Medicine, Aurora, CO, United States of America; 4 Children’s Hospital Colorado, Aurora, CO, United States of America; Texas A&M University, UNITED STATES

## Abstract

**Background:**

We have developed a mouse model of Parenteral Nutrition Associated Cholestasis (PNAC) in which combining intestinal inflammation and PN infusion results in cholestasis, hepatic macrophage activation, and transcriptional suppression of bile acid and sterol signaling and transport. In the liver, the master circadian gene regulators *Bmal/Arntl* and *Clock* drive circadian modulation of hepatic functions, including bile acid synthesis. Once activated, Bmal and Clock are downregulated by several transcription factors including Reverbα (*Nr1d1*), Dbp (*Dbp*), Dec1/2 (*Bhlhe40/41*), Cry1/2 (*Cry1/2*) and Per1/2 (*Per1/2*). The aim of this study was to examine the effects of PN on expression of hepatic circadian rhythm (CR) regulatory genes in mice.

**Methods:**

WT, IL1^KO^ or TNFR^KO^ mice were exposed to dextran sulfate sodium (DSS) for 4 days followed by soy-oil lipid emulsion-based PN infusion through a central venous catheter for 14 days (DSS-PN) and the expression of key CR regulatory transcription factors evaluated. Animals were NPO on a 14 hr light-dark cycle and were administered PN continuously over 24 hrs. Mice were sacrificed, and hepatic tissue obtained at 9-10AM (Zeitgeber Z+3/Z+4 hrs). PNAC was defined by increased serum aspartate aminotransferase, alanine aminotransferase, total bile acids, and total bilirubin and the effect of i.p. injection of recombinant IL-1β (200ng/mouse) or TNFα (200ng/mouse) on CR expression was examined after 4 hrs.

**Results:**

In the PNAC model, DSS-PN increased serum biomarkers of hepatic injury (ALT, AST, serum bile acids) which was suppressed in both DSS-PN IL1^KO^ and DSS-PN TNFR^KO^ mice. In WT DSS-PN, mRNA expression of *Arntl* and *Dec1* was suppressed corresponding to increased *Nr1d1*, *Per2*, *Dbp* and *Dec2*. These effects were ameliorated in both DSS-PN IL1^KO^ and DSS-PN TNFR^KO^ groups. Western analysis of the circadian transcription factor network revealed in WT mice DSS-PN significantly suppressed Reverbα, Bmal, Dbp, Per2 and Mtnr1b. With the exception of Dbp, DSS-PN mediated suppression was ameliorated by both IL1^KO^ and TNFR^KO^. Intraperitoneal injection of IL-1β or TNFα into WT mice increased serum AST and ALT and suppressed mRNA expression of *Nr1d1*, *Arntl* and *Clock* and increased *Dbp* and *Per2*.

**Conclusions:**

Altered expression of CR-dependent regulatory genes during PNAC accompanies cholestasis and is, in part, due to increased cytokine (IL-1β and TNFα) production. Evaluation of the effects of modulating CR in PNAC thus deserves further investigation.

## Background

Parenteral nutrition (PN) is a life-saving therapy in adults and infants who do not tolerate adequate enteral nutrition secondary to short bowel syndrome (SBS), necrotizing enterocolitis, bowel resections, or other causes of intestinal failure (IF). A significant complication in infants receiving PN, PN-associated cholestasis (PNAC; also called IF-associated liver disease [IFALD]) is characterized by cholestasis, increased hepatic steatosis, and ultimately hepatic fibrosis [1–4]. A critical factor in the development of PNAC is increased intestinal permeability resulting from intestinal inflammation, bacterial overgrowth and dysbiosis, promoting the increased absorption of bacterial lipopolysaccharides (LPS) into the enterohepatic circulatory system. These products then induce hepatic macrophage cytokine production and downregulation of hepatocyte bile and sterol transporters [4–8]. Importantly, plant phytosterols such as β-sitosterol and stigmasterol within the PN solution have an important role in PN-dependent macrophage activation and suppression of bile acid transport supporting dysregulation of bile acid homeostasis [4–12].

We have developed a mouse model in which intestinal injury (dextran sulfate sodium [DSS] enterally for 4 days) is combined with 2-week PN infusion to approximate the pathophysiology in PN-dependent infants with intestinal inflammation and altered barrier function [8]. In this model, liver injury and cholestasis as evidenced by increased serum bile acids, are associated with intestinal dysbiosis, increased intestinal permeability, increased LPS absorption into the portal vein, recruitment and activation of hepatic macrophages and upregulation of the proinflammatory cytokines Interleukin 1 beta (IL-1β) and tumor necrosis factor alpha (TNFα)[4, 8, 11, 12]. On a molecular level, macrophage activation and cholestasis in mice corresponded to suppression of hepatic transcription of Farnesoid X receptor (Fxr) [10], Liver receptor homolog 1 (Lrh1), and canalicular transporters for bile salts (bile salt export pump (BSEP), multidrug resistance–associated protein 2 (MRP2], and the plant sterol transporters sterolin 1 and 2 (Abcg5/g8) [4, 9]. These findings replicate data obtained from other models of PNAC including piglets [13–15] as well as data reported in children with PNAC and IFALD [6] who showed histologic and molecular evidence of liver inflammation as well as alteration of bile acid homeostasis.

**Fig 1:**
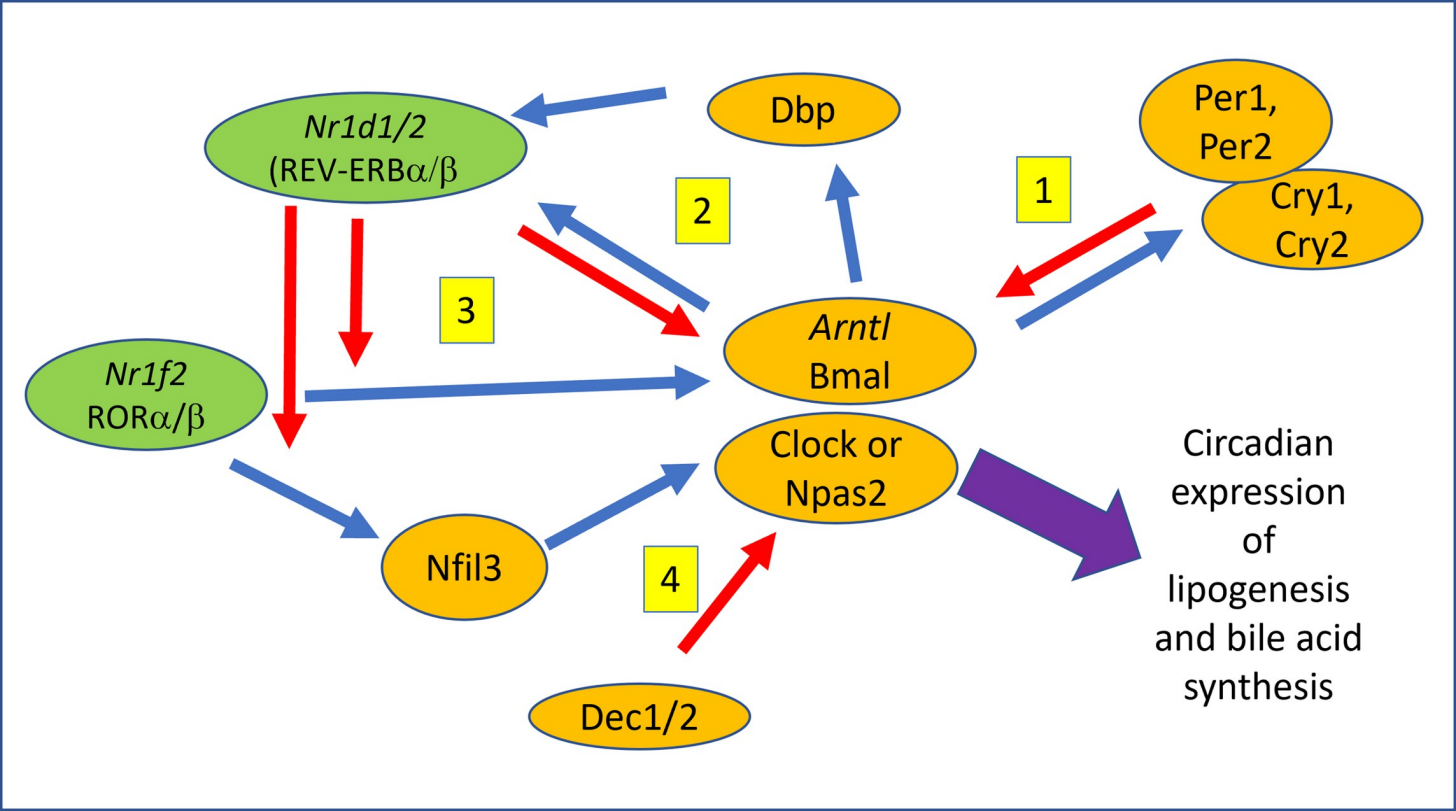
Summary of circadian regulatory feedback loops. In the liver, diurnal expression of metabolic pathways is controlled by light/dark as well as fast/feed cycles. Critical in this regulation is the transcription factor Bmal (Arntl). In the liver, Bmal regulates daily rhythmic expression of bile acid as well as lipid synthesis linking nutrition to homeostasis. Regulation of Bmal occurs via a network of nuclear receptor (Green) and transcription factors (orange) which form multiple feedback loops (Yellow) ensuring diurnal regulation of bile acid and lipid metabolism.

The mammalian circadian rhythm system is composed of a central neuronal clock located in the suprachiasmatic nucleus (SCN) of the hypothalamus as well as organ and cell specific peripheral clocks [16, 17]. Beyond the SCN, there are organ specific peripheral effects. In the liver, a majority of the metabolic pathways including synthesis and metabolism of cholesterol and bile acids are coordinated by circadian rhythms and are regulated by daily feeding and fasting cycles. Due to meal consumption, enzymes that regulate cholesterol and bile acid synthesis exhibit circadian rhythmicity resulting in diurnal rhythmic variation of cholesterol and bile acid concentrations [18, 19]. At the core of this network are two central transcription factors, Bmal1/2 (brain and muscle Arnt-like 1, *Arntl*) and Clock (circadian locomotor output cycles kaput) which bind together to form a heterodimer that binds to E-box containing promoters of genes resulting in diurnal expression (**Fig 1**).

The Bmal/Clock heterodimer is tightly regulated by several interlocking feedback inhibition loops. Initially, the Bmal/Clock complex is transcriptionally regulated by a feedback loop composed of the orphan nuclear receptors Reverbα (*Nr1d1*, receptor reverses erythroblastosis virus α/β), Rorα (*Nr1f1*, retinoid-related orphan receptors) and Nfil3 (E4bp4, nuclear factor, interleukin 3 regulated). Transcription of Rorα is repressed by Reverbα. When activated, Rorα can directly regulate both Bmal and Nfil3 expression. Hence, by regulating Rorα expression, Reverbα nuclear receptors rhythmically repress the transcription of Bmal1 and Nfil3 completing the loop. Separately, Nfil3 together with D-box binding protein (DBP), as well as CLOCK and Bmal1, form a feedback loop to rhythmically regulate Reverbα nuclear receptors [17]. Upon activation, Bmal/Clock regulates the transcription of Per1/2 (period 1/2) and Cry1/2 (cryptochrome 1/2) genes which as protein concentrations increase will in turn inhibit Bmal/Clock. Furthermore, as Bmal is downregulated, Per/Cry complexes undergo ubiquitination/proteolysis downregulating expression and renewing the cycle. Finally, recent data also support cyclic regulation of Bmal by Differentiated Embryonic Chondrocyte 1/2 (DEC1/2, encoded by *Bhlhe40/41*) which also regulate expression of each other forming an additional feedback loop [20].

Circadian rhythms play a prominent role in cholestatic liver diseases. In mice, caloric restriction during the nighttime feeding phase alters bile acid metabolism [21]. In bile duct ligation models, prolonged exposure to darkness improved liver pathology, reduced biochemical markers of liver injury and increased serum melatonin concentrations [22]. In recent studies, administration of exogenous melatonin, which is normally produced in the pineal gland, was shown to reduce expression of Bmal and reduce 28-day BDL induced liver injury [23]. Furthermore, deletion of melatonin receptor 1a ameliorated, whereas deletion of melatonin receptor 1b enhanced, BDL injury supporting circadian regulation in cholestatic liver disease [24]. At the genetic level, deletion of both Per1 and Per2 increased serum bile acid levels and ALT supporting the contribution of circadian rhythms in bile acid homeostasis and liver injury [25, 26]. Focusing on PNAC, PN administration in the absence of intestinal injury has been shown to alter the rhythmicity of both the central and the peripheral circadian machinery [27, 28]. Furthermore, diurnal PN administration reverses the rhythmic expression of Dbp and Per2 [27].

Proinflammatory cytokines have been shown to be important regulators of and are regulated by the mammalian circadian regulatory system. In cell culture and in mice, TNFα has been shown to suppress expression of Dbp, Per1 and Per2 [29]. Increased expression of RORα inhibits TNFα downstream effects [30]. IL-1b production by the Nlrp3 inflammasome has been shown to be regulated by Reverbα [31]. Furthermore, Il-1β has been shown to suppress Bmal expression [32]. In opposition, modulation of circadian transcription factor activity has been shown to alter proinflammatory cytokine production. Bmal is a metabolic sensor of IL-1β and modulation of Bmal expression by activation of the Bmal upstream regulator Reverbα suppresses IL-1β expression [33, 34].

The effects of continuous PN in the presence of intestinal injury on the circadian machinery has not been examined. Herein, we determined that expression of transcription factors that regulate circadian rhythms are dysregulated in murine PNAC. Furthermore, intraperitoneal injection of IL-1β or TNFα replicated suppression and genetic inhibition of either Il-1β or TNFα signaling restored normal circadian expression. We propose that the combination of parenteral nutrition and increased production of proinflammatory cytokines contributes to dysregulation of circadian rhythms in PNAC, which may further alter normal bile acid homeostasis and thus contribute to the pathogenesis of PNAC. These data suggest that targeting circadian rhythms should be explored for its therapeutic potential in infants with PNAC.

## Materials and methods

### Murine sample procurement

In male C57BL/6 mice (10 weeks old), the PNAC mouse model was employed using a combined oral DSS therapy for 4 days followed by 14 days of total PN through a central venous catheter (2). To examine the impact of inhibition of IL-1β or TNF signaling, male mice with deletion of IL1 (IL1r^KO^ C57BL/6; #003245 Jackson Laboratories, Bar Harbor, ME) or both TNFR1 and TNFR2 (TNFR^KO^ C57BL/6; #003243 Jackson Laboratories) were treated with either Chow or DSS-PN as described above (Chow/TNFR^KO^, DSS-PN/TNFR^KO^, Chow IL1^KO^ or DSS-PN IL1^KO^) [4]. A select subset of male C57BL/6 wild-type (WT) mice were administered recombinant IL-1β (intraperitoneal [i.p.] 8 mg/kg in phosphate buffered saline (PBS), ∼200 ng/mouse, [#401-010CF; R&D Systems, Minneapolis, MN]) or TNFα (intraperitoneal [i.p.] 8 mg/kg in PBS, ∼200 ng/mouse, [#410-MT/CF; R&D Systems, Minneapolis, MN]) and euthanized 4 h later (Z+5). A separate set of Chow-fed only mice were injected with vehicle and sacrificed at the same time. Unless otherwise stated, all mice were sacrificed at Zeitgeber+1. On the day of euthanasia, mice were anesthetized with i.p. pentobarbital and blood collected from the retro-orbital plexus and liver removed, placed in formalin or snap frozen in liquid nitrogen and subsequently stored at –80°C until analyzed. To ensure rigor and reproducibility, all samples were randomly coded and blindly analyzed. All animal care and procedures were performed and approved under the University of Colorado Anschutz Medical Campus Institutional Animal Care and Use Committee (protocol number 00000879). All studies involving animal experiments conformed with the Animal Research: Reporting of In Vivo Experiments (ARRIVE) guidelines [35].

### Immunohistochemical evaluation

Formalin fixed slides were analyzed for hepatic macrophages using antibodies directed against F4/80 (1:250, T-2028, Rat anti-mouse monoclonal, BMA Biomedicals, Rheinstrasse Switzerland), CK7 (1:500, ab181598, Rabbit monoclonal, Abcam, Boston, MA), and Ki67 (1:100, M7249 TEC3, Rat anti-mouse monoclonal, Dako, Carpinteria, CA) as previously described [36]. Heat induced antigen retrieval was performed in Dako target Antigen Retrieval Solution (Dako, Carpinteria, CA). Following incubation with primary antibodies overnight, slides were washed 3X5min in tris-buffered saline 1% tween and incubated in HRP-conjugated a goat anti-rat, goat anti-rabbit (MP-7444, MP7401, Vector Laboratories, Newark, CA) secondary antibody for 30 minutes. The peroxidase substrate used was IMMPACT-DAB (SK-4105, Vector Laboratories). Histologic images were captured on an Olympus BX51 microscope equipped with a four-megapixel Macrofire digital camera (Optronics; Goleta, CA) using the Picture Frame Application 2.3 (Optronics). All images were cropped and assembled using Adobe Photoshop Elements (Adobe Systems, Inc.; Mountain View, CA). For quantification of IHC staining, 10 images per animal were obtained at 100X magnification (tiling). All images were imported into SlideBook 6.0 (Intelligent Imaging Innovations, Denver, Colorado; Colin Monks) and pixels per image quantified.

### Quantitative PCR

qRT-PCR to examine mRNA expression was performed using TaqMan probes from Applied Biosystems (Foster City, CA) as previously described (**Table 1**) [4].

**Table 1:** Primers used for qRT-PCR in this study.

Gene	Primer
Nr1d1	Mm00520708
Cry1	Mm00514392
Nfil3	Mm00600292
Npas2	Mm00500848
Bhlhe40	Mm00478593
Arntl	Mm00500223
Cry2	Mm01331539
Clock	Mm00455950
Dbp	Mm00497539
Per2	Mm00478099
Nr1f1	Mm01173766
Bhlhe41	Mm00470512
Per1	Mm00501813
Hprt	Mm03024075

### Western blotting

Western blots were performed liver homogenate using 30μg of protein per lane as previously described [37, 38]. The antibodies used in this study are listed in **Table 2**. With the exception of Gapdh, which was used at 1:20,000, all other antibodies were used at a dilution of 1:1,000. The secondary antibodies were obtained from Cell Signaling and used at a dilution of 1:10,000.

**Table 2:** Antibodies used for Western analysis in this study.

Protein	Source	Cat#
Mtnr1a	Novus	NBP1-28912
Mtnr1b	Novus	NLS932
Per2	Novus	NB100-125
Reverba	Protein Tech	14506-1-ap
Dbp	Protein Tech	12662-1-ap
RORa	Biolegend	683102
Bmal	Novus	NB100-2288
GAPDH	Millipore	MAB374
Dec2	Novus	NBP1-19613
Dec1	Novus	NB100-1800

### Statistical analysis

The data are presented as means ± Standard Error of the Mean (SEM). Comparisons between WT and DSS-PN tissue was accomplished by Student’s T-tests or by 1-way analysis of variance (ANOVA). Comparisons between genotypes was accomplished by 1-way ANOVA. Statistical significance was set at P<0.05. Prism 5 for Windows (GraphPad Software, San Diego, CA) was used to perform all statistical tests.

## Results

### The expression of circadian rhythm regulatory transcription factors is altered in murine PNAC

**Fig 2:**
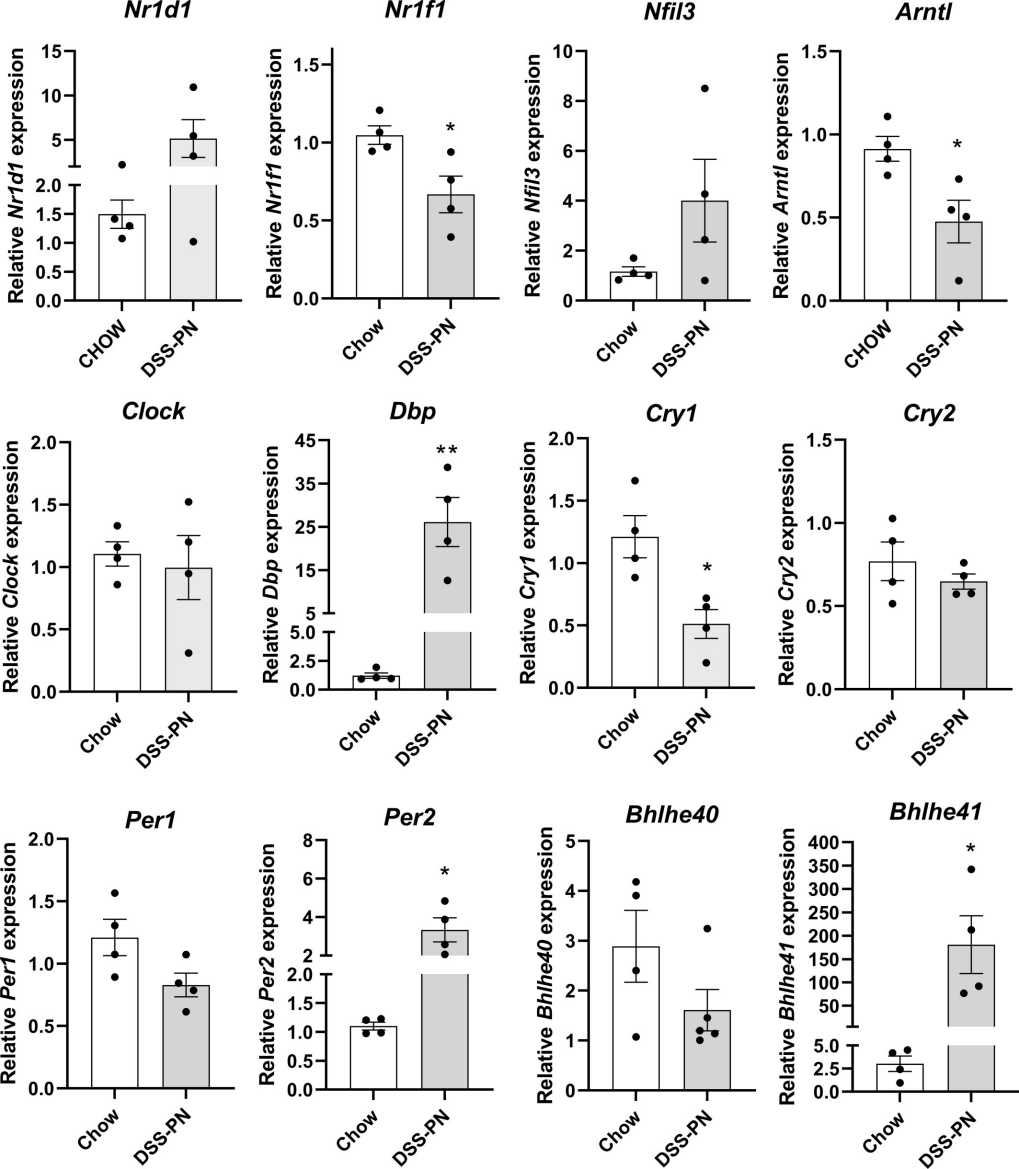
Parenteral nutrition dysregulates circadian regulatory expression in mice. **mRNA expression of circadian** transcription factors in hepatic tissue isolated from Chow and DSS-PN mice (Z+2/Z+3). Expression was normalized against HPRT. N = 4 per condition. Values are Mean± SEM. *p<0.05. **p<0.01.

Mice were subjected to 4D DSS followed by 14D administration of continuous TPN [11]. Using qRT-PCR, mRNA expression of the circadian machinery was examined in hepatic tissue extracted from Chow and 14d DSS-PN mice sacrificed between Z+2 and Z+3 (**Fig 2**). In the DSS-PN group, mRNA expression of *Arntl1* and *Cry1* was suppressed whereas *Per2*, *Bhlhe41* and *Dbp* was significantly increased. No significant changes were evident in the expression of *Nr1d1*, *Clock*, *Per1*, *Cry2*, *Nfil3* and *Bhlhe40*.

### Short term exposure of IL-1β or TNFα impacts hepatic circadian regulatory transcription factors mRNA expression

**Fig 3:**
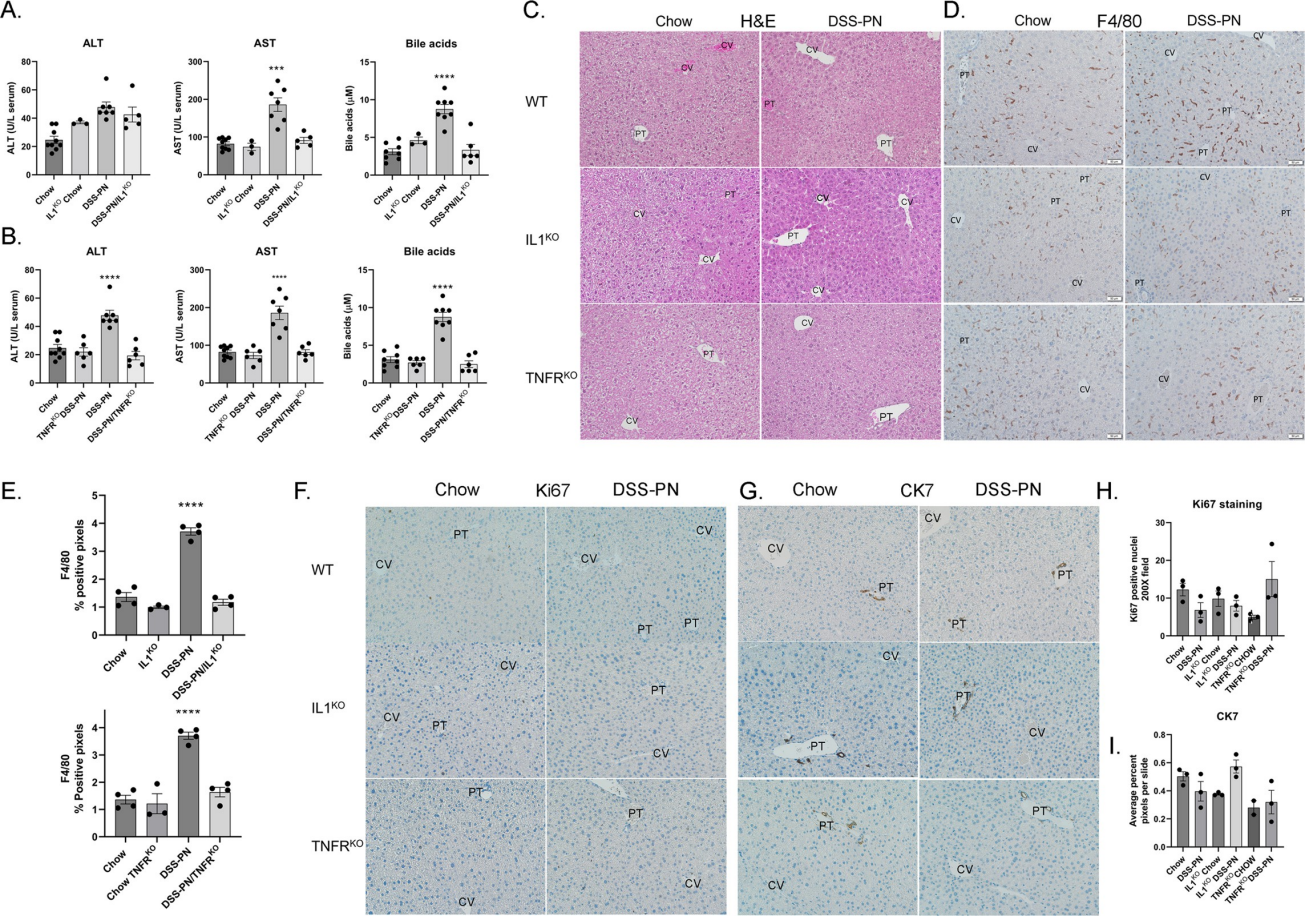
Genetic inhibition of IL-1β or TNFα signaling ameliorates DSS-PN induced liver injury. Wild-type (WT) or (**A**) IL1^KO^ or (**B**)TNFR^KO^ mice were treated with chow or DSS-PN and euthanized after 14 days [4, 11]. Liver injury was assessed using serum (Alanine aminotransferase (ALT), Aspartate aminotransferase (AST), Total serum bile acids). N = 3–9 per condition. Values are Mean± SEM, ***p<0.001. ****p<0.0001. **C.** Hematoxylin and Eosin (H&E) staining of liver sections isolated from indicated conditions. **D**. Immunohistochemical analysis of F4/80+ macrophages in liver sections isolated from indicated conditions, N = 3 per condition, 200X, PT, Portal triad; CV, Central vein. **E**. Quantification of F4/80 positive staining. Values are Mean± SEM, ****p<0.0001. **F**. Immunohistochemical analysis of Ki67 positive nuclei in liver sections isolated from indicated conditions, N = 3 per condition, 200X. **G**. Immunohistochemical analysis of Cytokeratin 7 (CK7) staining liver sections isolated from indicated conditions, N = 2–3 per condition, 200X. **H.** Quantification of Ki67 positive staining/100X field, values are Mean± SEM. **I.** Quantification of CK7 staining, values are Mean± SEM.

**Fig 4:**
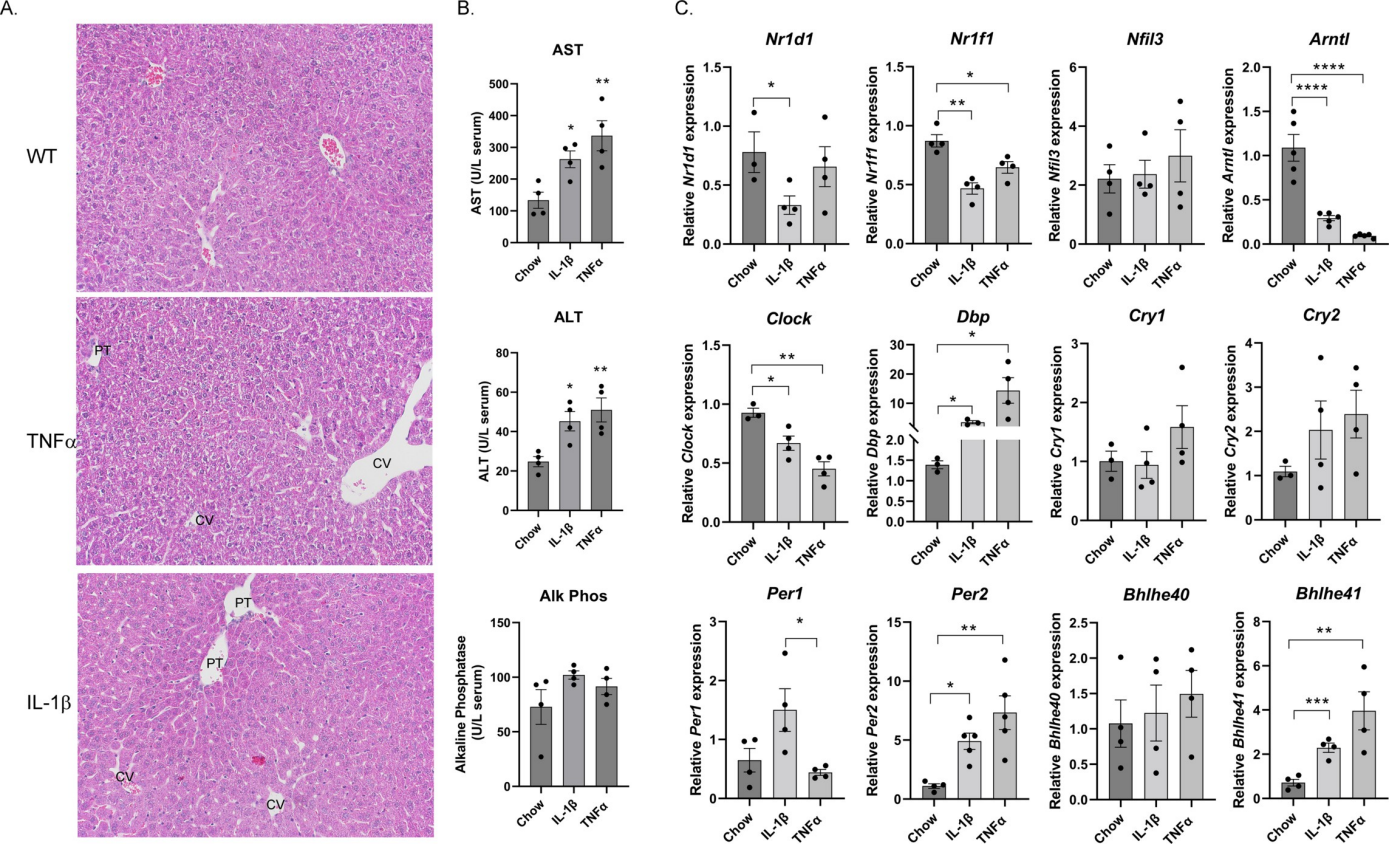
Administration of recombinant IL-1β and TNFα increases liver injury and rapidly alters transcription of the circadian machinery. 8–10 week old C57BL6 mice were injected with recombinant IL-1β (200ng/mouse/i.p.) or TNFα (200ng/mouse/i.p.) and sacrificed after 4hr. Serum and liver tissue was harvested, and **A**. Hematoxylin and Eosin staining of liver sections isolated from each condition. **B**. Liver injury assessed by serum ALT, AST and alkaline phosphatase. **C**. mRNA expression of transcription factors regulating hepatic CR analyzed by qRT-PCR. Expression was normalized against HPRT. Values are Mean± SEM. *p<0.05. **p<0.01, ***p<0.001, ****p<0.0001, N = 3–4 per condition.

We have previously shown that increased expression of the proinflammatory cytokines IL-1β and TNFα are important factors in the pathogenesis of PNAC [8, 11]. Importantly, inhibition of either IL-1β or TNFα signaling ameliorated hepatic inflammation and injury (**Fig 3A–3E**), [4, 11]). The impact of DSS-PN on cellular proliferation has not been examined. Immunohistochemical analysis of Ki67 was performed using tissue sections isolated from WT, TNFR^KO^ or IL1^KO^ Chow or DSS-PN and quantified. Treatment with DSS-PN no inhibition of IL-1β or TNFα signaling had no significant effect on cellular or ductular proliferation as evidenced by quantification of Ki67 positive cells and CK7 staining (**Fig 3F–3I**).

To determine the effect of increased IL-1β and TNFα on circadian rhythms, mice were injected with recombinant IL-1β (200/ng/mouse i.p.) or recombinant TNFα (200ng/mouse i.p.), sacrificed 4 hours later and liver injury and mRNA expression of circadian regulatory genes assessed (**Fig 4A–4C**). Compared to Chow controls, intraperitoneal injection (i.p.) of IL-1β and TNFα significantly increased serum AST and ALT but had no effect on alkaline phosphatase. Examining pathology, H&E staining revealed no differences between the groups. IL-1β and TNFα both markedly suppressed mRNA expression of circadian transcription factors *Arntl*, *Clock* and *Nr1f1*. IL-1β and TNFα both increased expression of *Dbp*, *Per2* and *Bhlhe41* but did not significantly affect *Cry1*, *Cry2* or *Bhlhe40*. Interestingly, suppression of *Nr1d1* was only present in the IL-1β injected group.

### Inhibition of IL-1β signaling restores expression of circadian rhythm regulatory transcription factors during murine PNAC

**Fig 5:**
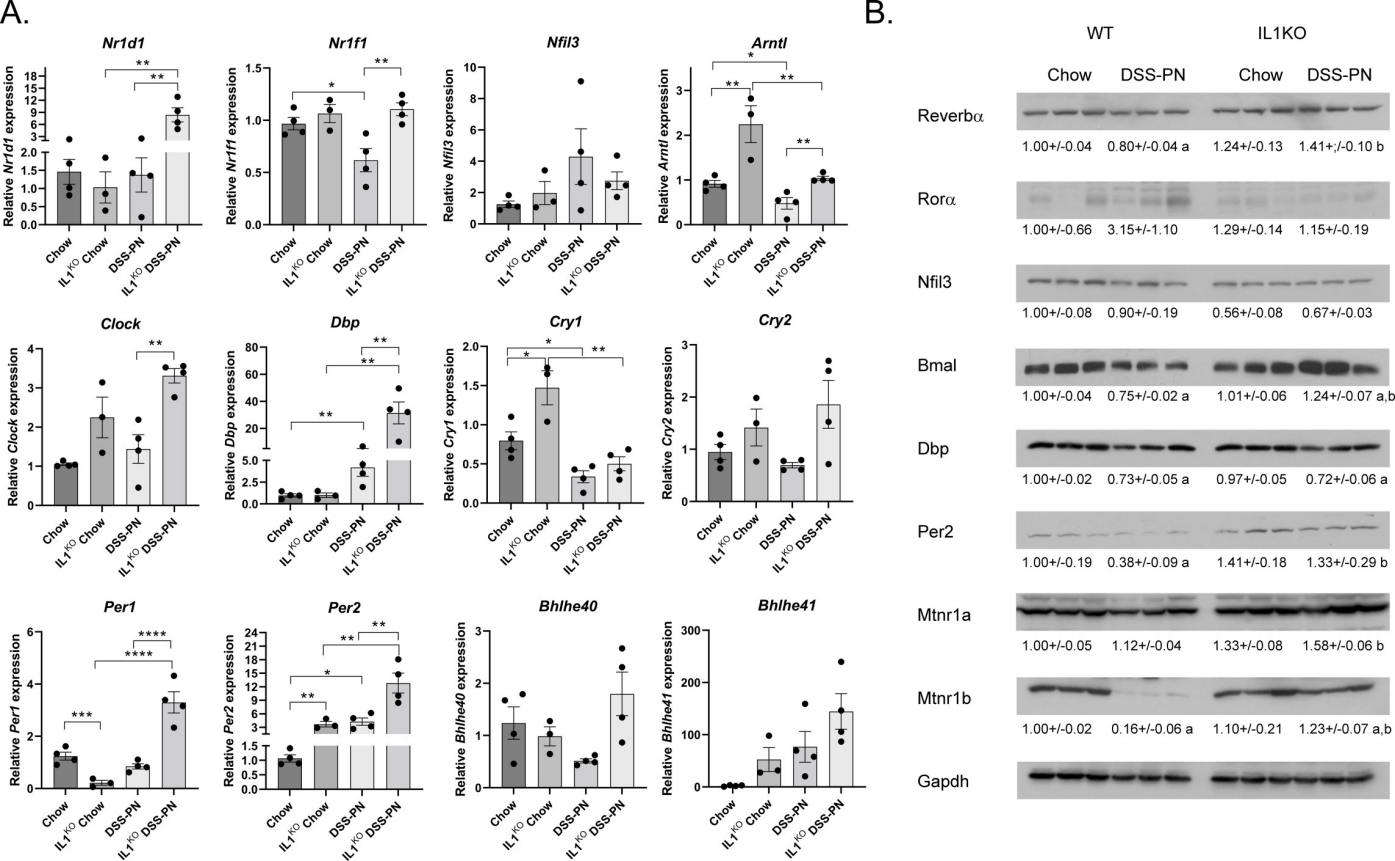
Deletion of IL-1β signaling ameliorates expression of hepatic circadian transcription factors following DSS-PN. 8–10 week old IL1^KO^ mice were subjected to Chow feeding or DSS-PN. **A**. Liver tissue was harvested, and mRNA expression of transcription factors regulating hepatic CR analyzed by qRT-PCR. Expression was normalized against HPRT. Values are Mean± SEM, N = at least 3/condition. *p<0.05. **p<0.01, ***P<0.001, ****P<0.0001. **B**. Western analysis of circadian regulatory proteins. Expression was normalized using Gapdh expression for each blot. Values are Mean± SEM, N = 3/condition, a = significantly different from respective chow control, b = significantly different compared to WT DSS-PN.

To determine if IL-1β signaling plays a role in the observed PNAC dependent dysregulation of expression of circadian regulatory genes, mRNA analysis of the circadian regulatory machinery was performed using Chow and DSS-PN treated IL1^KO^ mice (**Fig 5A**). Compared to the WT DSS-PN group, IL1^KO^ mice, either increased or restored expression of *Nr1d1*, *Nr1f1*, *Arntl*, *Clock*, *Dbp*, *Per1* and *Per2*. No significant differences were evident in *Nfil3*, *Cry1*, *Cry2*, *Bhlhe40*, *Bhlhe41* expression. Interestingly, comparing WT Chow and IL1^KO^ Chow, expression of *Arntl*, *Cry1*, and *Per2* increased whereas *Per1* was suppressed.

We next sought to determine if DSS-PN in combination with suppression of IL1β signaling impacted protein expression using Western analysis of Reverbα, Rorα, Nfil3, Bmal, Dbp, Per2, and the Melatonin receptors 1a and 1b (Mtnr1a, Mtnr1b). In WT mice, DSS-PN significantly suppressed Reverbα, Bmal, Dbp, Per2 and Mtnr1b but had no significant effect on Rorα, Nfil3, or Mtnr1a (**Fig 5B**). Inhibition of IL1β signaling ameliorated DSS-PN mediated suppression of Reverbα, Bmal, Per2and Mtnr1b. Interestingly, compared to WT DSS-PN, IL1^KO^ significantly increased Mtnr1a expression. No effect was evident on Dbp expression when comparing genotypes.

### Inhibition of TNFα signaling restores expression of circadian rhythm regulatory transcription factors during murine PNAC

**Fig 6:**
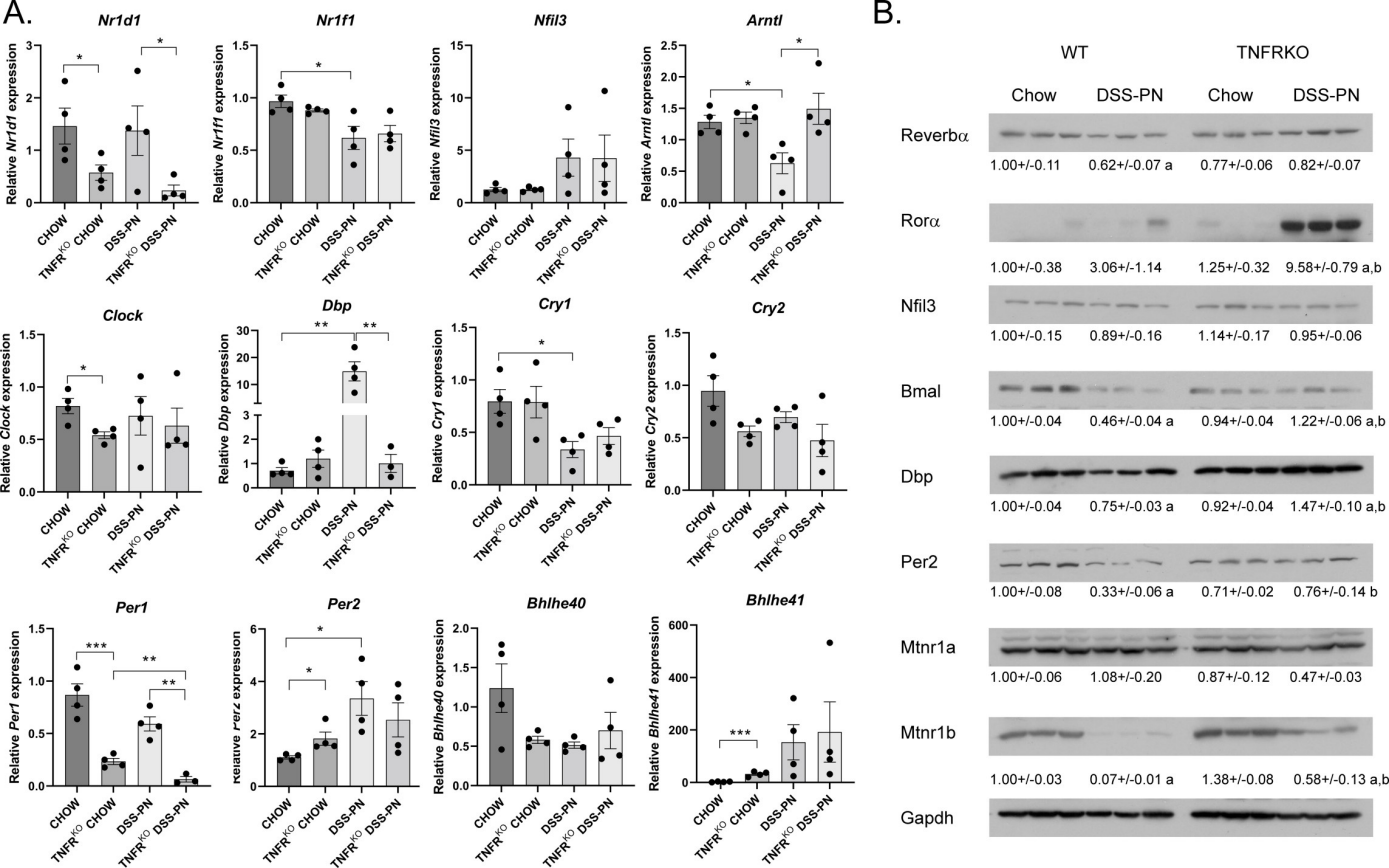
Genetic inhibition of TNFα signaling normalizes expression of hepatic circadian transcription factors following DSS-PN. 8–10 week old TNFR^KO^ mice were subjected to Chow feeding or DSS-PN. **A.** Liver tissue was harvested, and mRNA expression of transcription factors regulating hepatic CR analyzed by qRT-PCR. Expression was normalized against HPRT. N = 4 per condition. Values are Mean± SEM. *p<0.05. **p<0.01, ***p<0.001. **B.** Western analysis of circadian regulatory proteins. Expression was normalized using Gapdh expression for each blot. Values are Mean± SEM, N = 3/condition, a = significantly different from respective chow control, b = significantly different compared to WT DSS-PN.

The proinflammatory cytokine TNFα plays an important role in PNAC pathogenesis [6]. Pharmacological and genetic inhibition of TNFα signaling is protective in murine PNAC [4]. To determine if TNFα signaling plays a role in DSS-PN dependent dysregulation of circadian regulatory genes, mRNA analysis of the circadian machinery was performed using Chow and DSS-PN treated TNFR^KO^ mice [4]. Compared to WT Chow, inhibition of TNFα signaling suppressed *Nr1d1*, *Clock*, *Per1* and increased *Per2* and *Bhlhe41* expression (**Fig 6A**). Compared to WT DSS-PN, inhibition of TNFα signaling suppressed *Nr1d1*, *Dbp* and *Per1* and increased *Arntl* expression. No significant difference in expression was evident in *Nr1f1*, *Nfil3*, *Clock*, *Cry1*, *Cry2*, *Per2*, and *Bhlhe40*.

We next sought to determine if DSS-PN in combination with suppression of TNFα signaling impacted protein expression using Western analysis of Reverbα, Rorα, Nfil3, Bmal, Dbp, Per2, and the Melatonin receptors 1a and 1b (Mtnr1a, Mtnr1b). In WT mice, DSS-PN significantly suppressed Reverbα, Bmal, Dbp, Per2 and Mtnr1b but had no significant effect on Rorα, Nfil3, or Mtnr1a (**Fig 6B**). Inhibition of TNFα signaling ameliorated DSS-PN mediated suppression of Bmal, Dbp, Per2 and increased Mtnr1b albeit not to normal Chow levels. Surprisingly, RORα expression was significantly increased in TNFR^KO^ DSS-PN fed mice.

## Discussion

In this study, we utilized an established murine PNAC model that combines intestinal injury, increased permeability, and absorption of bacterial products with infusion of a soybean-oil based PN solution and demonstrate that expression of circadian regulatory genes is significantly altered in murine PNAC. Mechanistically, administration of recombinant TNFα or IL-1β similarly altered normal hepatic circadian gene expression and genetic disruption of IL-1β or TNFα signaling normalized mRNA and protein expression of some but not all circadian regulatory genes following DSS-PN administration. Overall, increased expression of the proinflammatory cytokines TNFα and IL-1β play an important role in the dysregulation of circadian regulatory genes and hepatic injury during PNAC and targeting proinflammatory cytokines may be an effective therapeutic strategy.

Total parenteral nutrition bypasses signaling originating from the normal gut-liver axis in response to feedings and results in an altered rhythm of the peripheral circadian clock. We and others have shown that in PNAC, bile acid homeostasis is disrupted resulting in increased serum and hepatic bile acid accumulation [15, 25, 39, 40]. Significant changes in the transcript level of the clock components *Arntl*, *Dbp*, *Per2*, *Cry1* and *Bhlhe41* were observed in DSS-PN treated compared to Chow-fed mice supporting an effect of DSS-PN on clock genes. Although PN administration was continuous in the present study, data herein are supported by previous evidence demonstrating that expression of Per2 and Dbp are dysregulated following diurnal administration compared to nocturnal PN administration [27].

Proinflammatory cytokines play a critical role in the development of PNAC [4, 5, 8, 11, 41]. Both the response to and the response from proinflammatory cytokines are rhythmically regulated. Hepatocyte specific deletion of Bmal1 increases hepatic LPS sensitivity [42]. In PNAC, increased intestinal derived LPS contributes to hepatic macrophage activation and production of proinflammatory cytokines including IL-1β and TNFα [8]. In cell culture, increased LPS suppresses Bmal expression corresponding to inflammasome activation and IL-1β production [33]. Furthermore, exposure to IL-1β or TNFα also suppresses Bmal expression supporting LPS downstream effects [32]. In mice, chronic exposure to high concentrations of TNFα (1.5μg/day) suppresses Dbp and Per2 expression [29]. In the current study, i.p. injection of a moderate dose of IL-1β (200ng/mouse) or TNFα (200ng/mouse) rapidly suppressed expression of key circadian transcription factors (*Arntl*, *Npas2*, *Clock*) while increasing *Per2* and *Dbp*. Interestingly, Per1 mRNA expression was increased by IL-1β but TNFα had no effect suggesting cytokine specific effects.

Data herein demonstrates that suppression of Mtnr1b by DSS-PN was ameliorated by inhibition of cytokine signaling. Previous research has shown that suppression of Mtnr1b has been shown to enhance cholestatic injury in the Mdr2^KO^ mice supporting the Mtnr1b as a contributor during DSS-PN mediated hepatic injury [24]. Concurrently, these data also support a role for proinflammatory cytokines in mediating Mtnr1b suppression. In acute cholestatic injury, administration of Melatonin and activation of melatonin-dependent downstream signaling has been shown to reduce both inflammation and oxidative stress both of which contribute to hepatic injury following DSS-PN [8, 11, 23, 43]. Overall, data support the contribution of Mtnr1b in hepatic injury during DSS-PN and that suppression of Mtnr1b is mediated in part through proinflammatory cytokine signaling.

Proinflammatory cytokine i.p. injection data herein support data obtained from DSS-PN fed mice, where concentrations of IL-1β and TNFα have been previously shown to be increased [4, 11]. Furthermore, the present data show that in DSS-PN mice, IL1^KO^ as well as TNFR^KO^ restores expression of some but not all circadian regulatory transcription factors supporting the role of proinflammatory cytokines in the dysregulation of hepatic circadian transcription factors during PNAC. The peripheral hepatic circadian transcription network is regulated in part by signals originating from the gut following feeding. Of critical importance, while mice are on TPN, they are NPO which bypasses normal gut-liver circadian regulation. This suggests that NPO is not playing a significant role in modulating the peripheral hepatic circadian transcription factor network. Instead, our data support proinflammatory cytokines as significant contributors in the dysregulation of circadian transcription factor expression. Furthermore, DSS-PN administration suppressed Dbp as well as Nr1d1 in TNFR^KO^ but increased Dbp and Nr1d1 expression in IL1^KO^ suggesting inhibition of specific cytokine signaling pathways may have differential effects on circadian transcription factor expression. The ramification of these differences in cytokine-dependent regulation during DSS-PN remains to be explored.

In conclusion, in the present study, we show that peripheral expression of the circadian transcriptional machinery is altered in a murine model of PNAC. Genetic inhibition of either IL-1β or TNFα signaling restored expression of some but not all circadian regulators, supporting a role of proinflammatory cytokines in the regulation of hepatic CR during PN. Noteworthy, Mtnr1b was significantly suppressed by DSS-PN and restored by proinflammatory cytokine inhibition. There are some limitations to this study. For example, we examined gene and protein expression at a single timepoint during the day (Z+2/Z+3). It is unknown if DSS-PN is impacting the amplitude of circadian expression or if it is impacting the rhythm as a whole. Concurrently, this study used whole liver extracts. Cell-specific (e.g. macrophages, hepatocytes, cholangiocytes) effects of PN on circadian transcription factor expression have not been examined. Furthermore, continuous PN feeding was employed for this experiment. The effects of nocturnal or diurnal PN administration, which is used in humans, on hepatic circadian transcription factor expression and activation have not been fully elucidated. Thus, an in-depth examination of circadian regulation of both bile acid and lipid synthesis and metabolism during PN with respect to hepatic injury is indicated. We propose that the combination of PN and increased production of proinflammatory cytokines contributes to dysregulation of circadian rhythms in PNAC and that targeting expression of circadian transcription factors and therefore their downstream targets should be further explored as a potential therapeutic intervention for PNAC.

## Supporting information

S1 Raw images (PDF)

## Abbreviations

ALT: alanine aminotransferase
Arntl: brain and muscle Arnt-like 1
AST: aspartate aminotransferase
Bmal1: brain and muscle Arnt-like 1
CLOCK: circadian locomotor output cycles kaput
CR: Circadian Rhythm
Cry1/2: cryptochrome ½
Dbp: D-site binding protein
DEC1/2: differentiated embryonic chondrocyte
DSS: dextran sulfate sodium
FXR: Farnesoid X Receptor
IF: intestinal failure
IFALD: Intestinal failure associated liver disease
Il-1β: interleukin 1 beta
i.p: Intraperitoneal
LPS: lipopolysaccharide
Nfil3, E4bp4: nuclear factor, interleukin 3 regulated
Mtnr1a: Melatonin receptor 1a
Mtnr1b: Melatonin receptor 1b
Per1/2: period ½
PN: parenteral nutrition
PNAC: parenteral nutrition associated cholestasis
Reverbα: receptor reverses erythroblastosis virus α
Rorα: retinoid-related orphan receptor α
SBS: short bowel syndrome
TNFα: tumor necrosis factor alpha
TNFR: tumor necrosis factor receptor
WT: wild type
