# Supplementary material for: Expression of circadian regulatory genes is dysregulated by increased cytokine production in mice subjected to concomitant intestinal injury and parenteral nutrition
Source: PLoS One. 2023 Aug 30;18(8):e0290385. doi: 10.1371/journal.pone.0290385 (PMC10468060; doi:10.1371/journal.pone.0290385)
Supplement: S1 Raw images — (PDF) [file pone.0290385.s001.pdf]

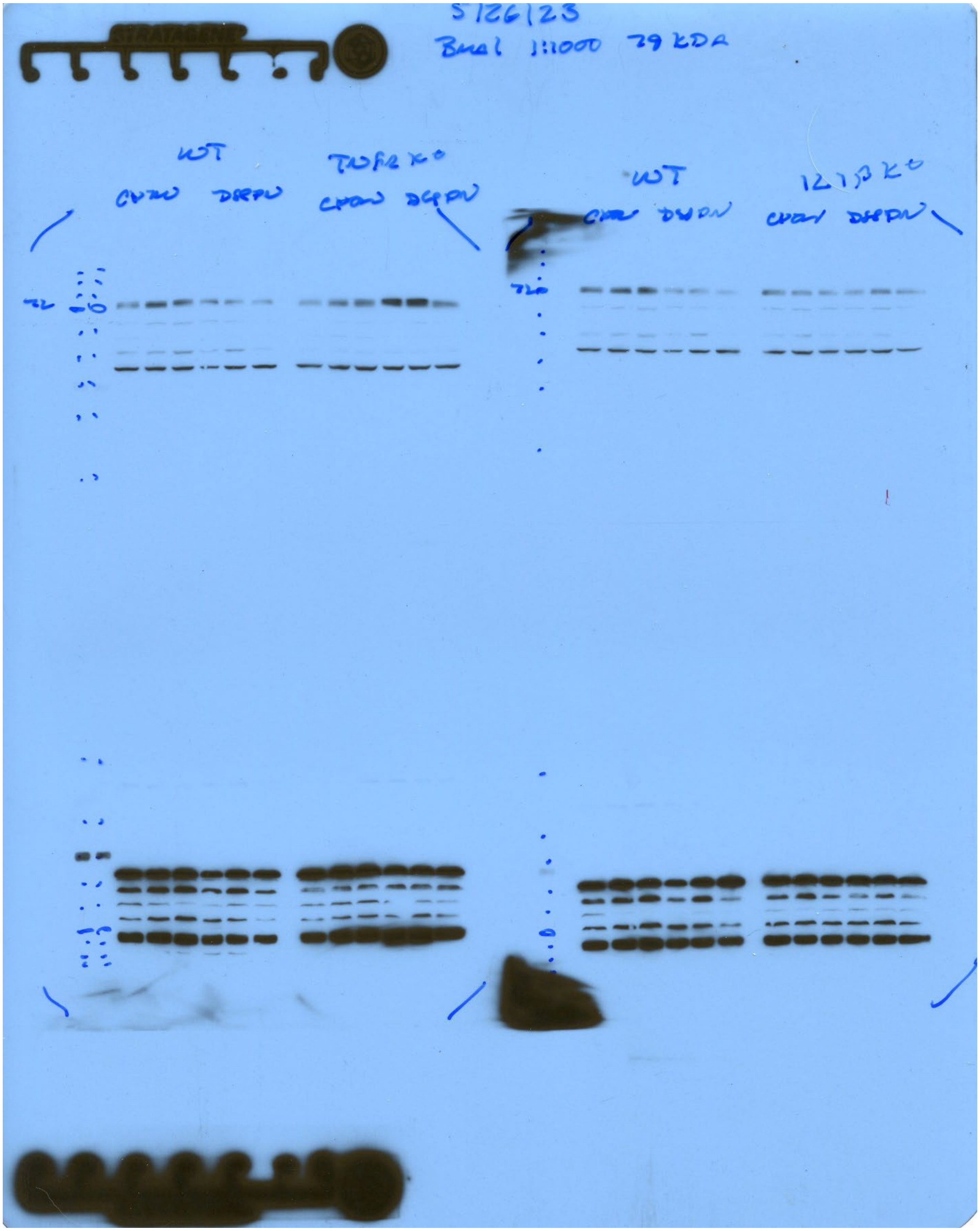
5/26/23
Bmal 1:1000 79 kDA
WT
WT
STRATAGENE

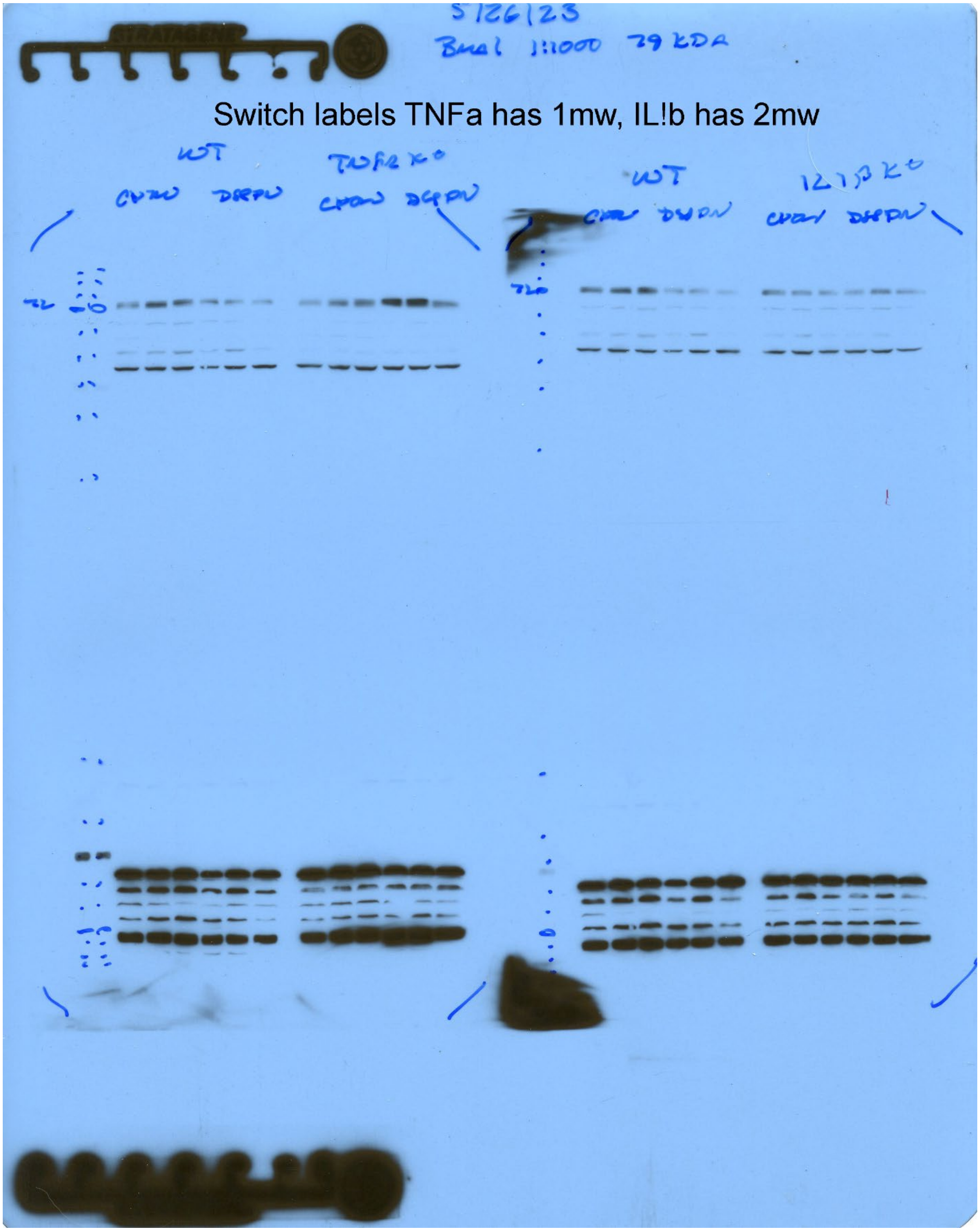
5/26/23
Switch labels TNFa has 1mw, IL!b has 2mw
WT
WT

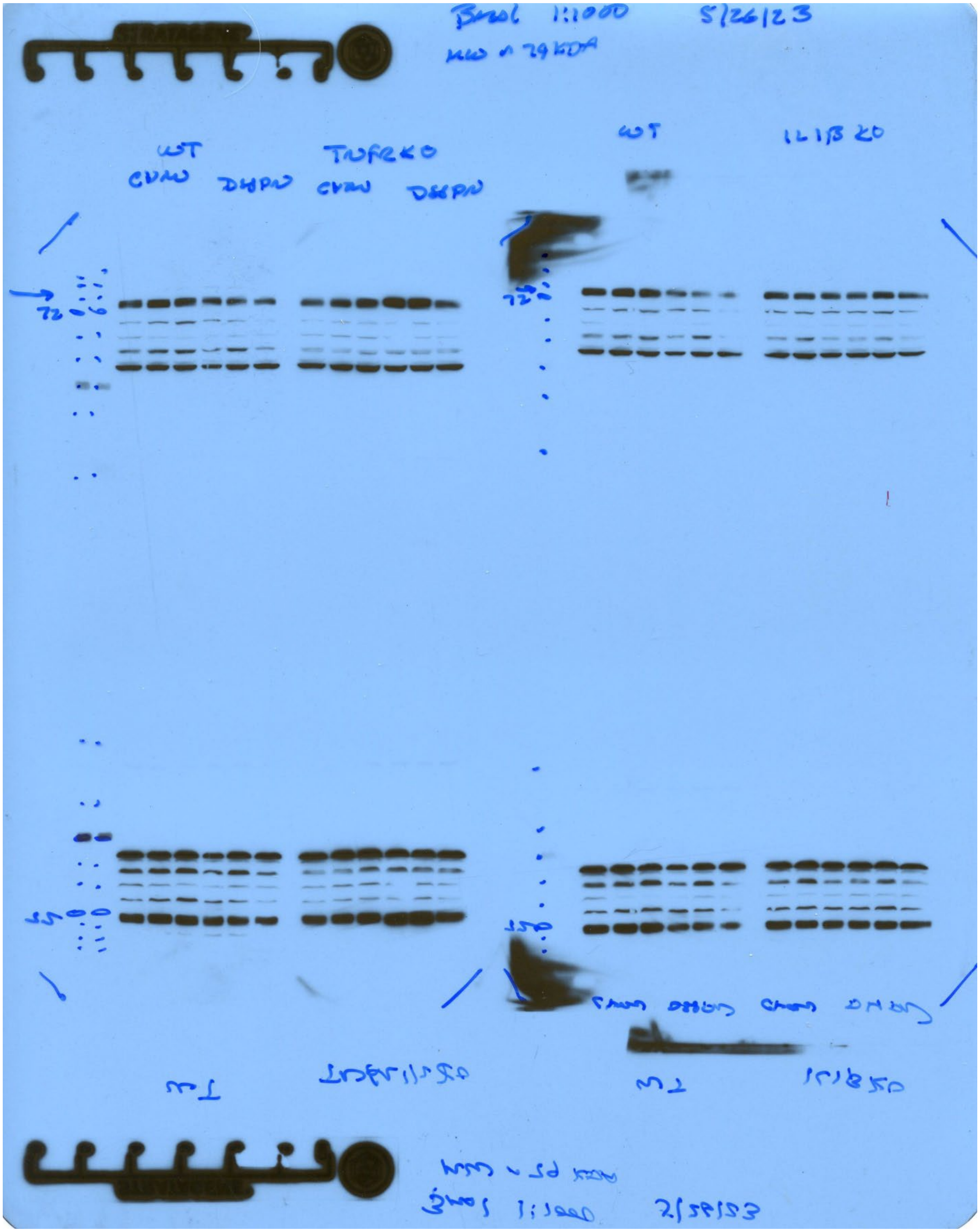
1:1000
5/26/23
WT
TNFRKO
WT
IL1β KO
72
72
55
150

Switch TNFa has 1mw, IL1r has 2mw

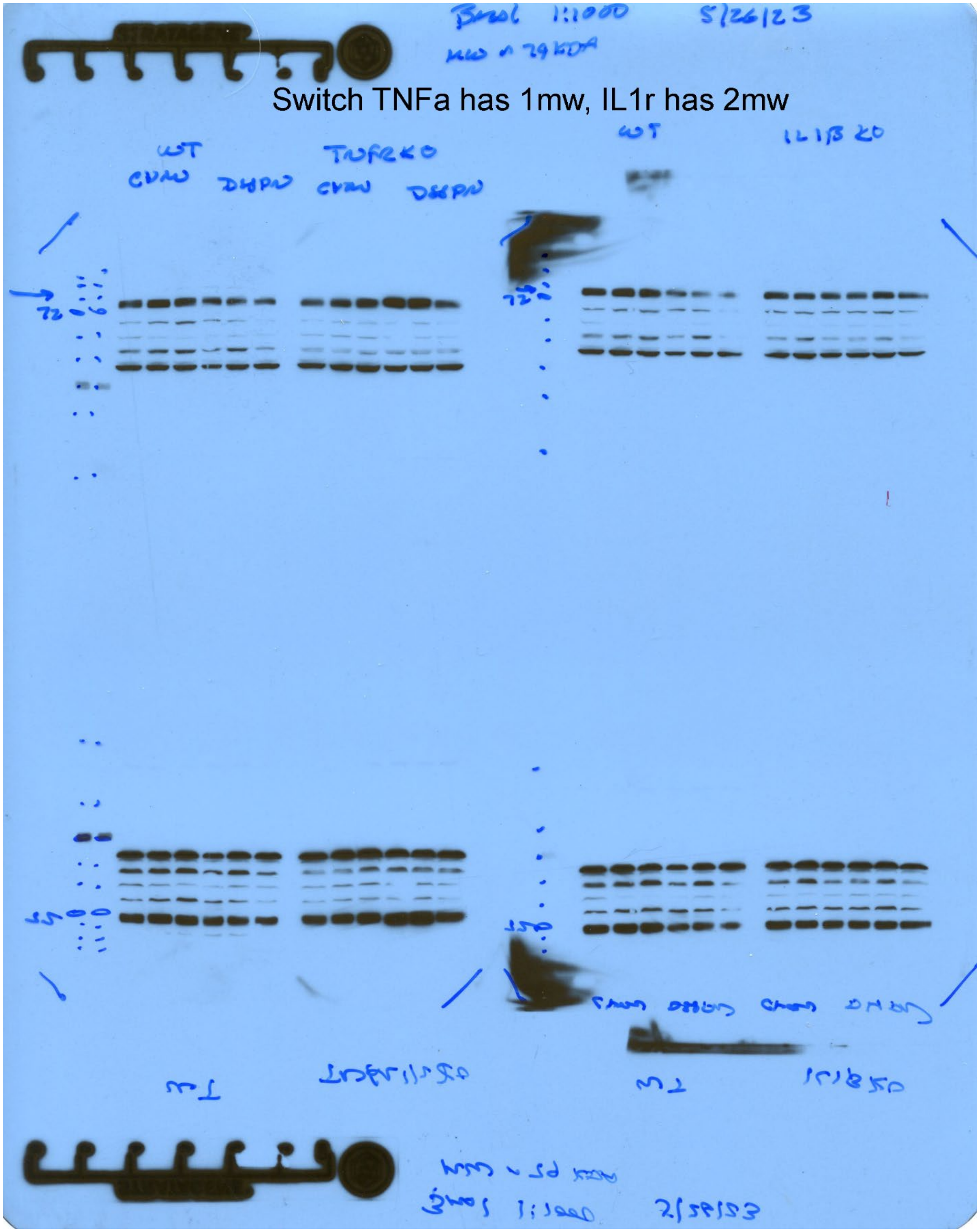

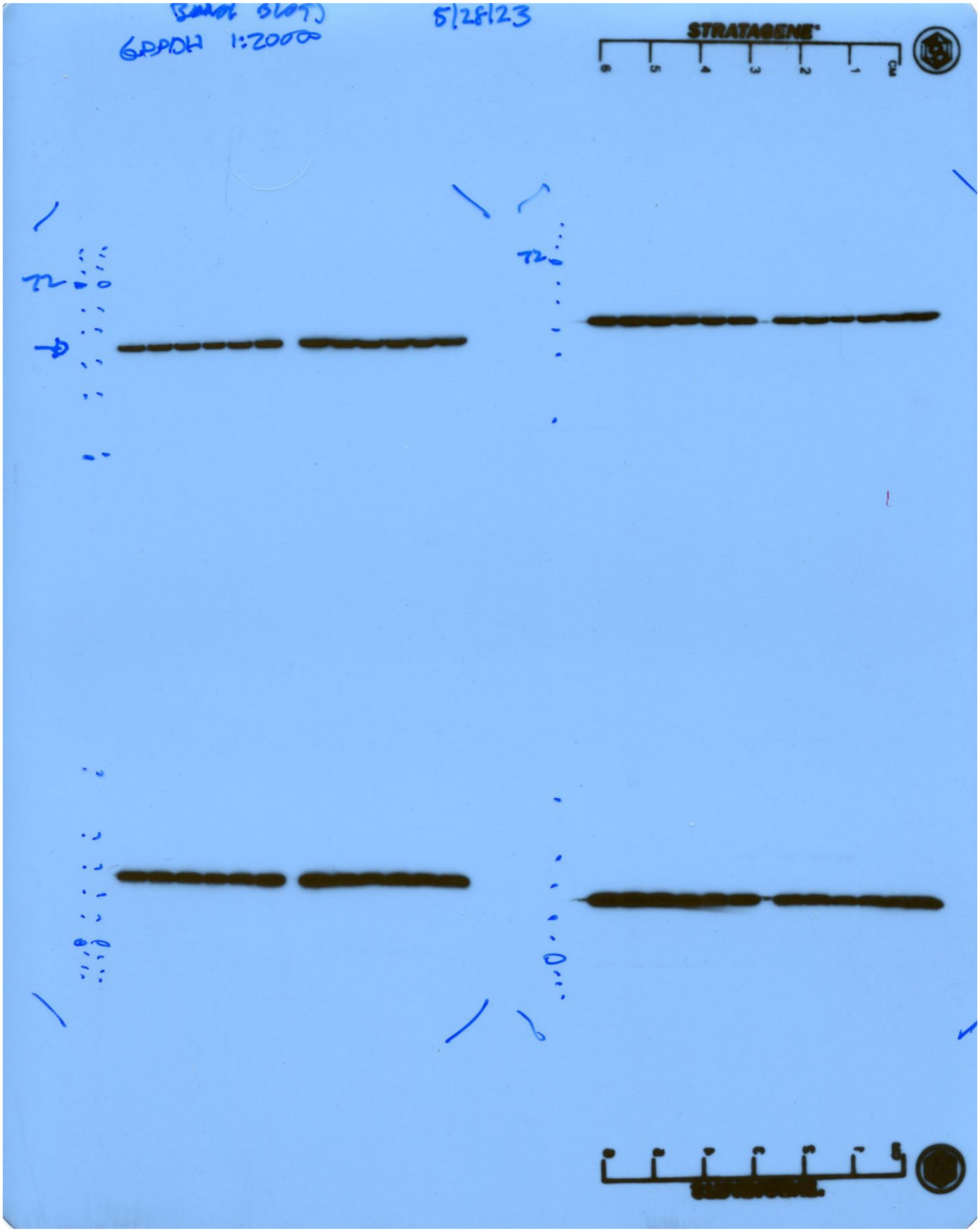
8/28/23
GAPDH 1:20000
STRATAGENE
6 5 4 3 2 1 CM
72
72

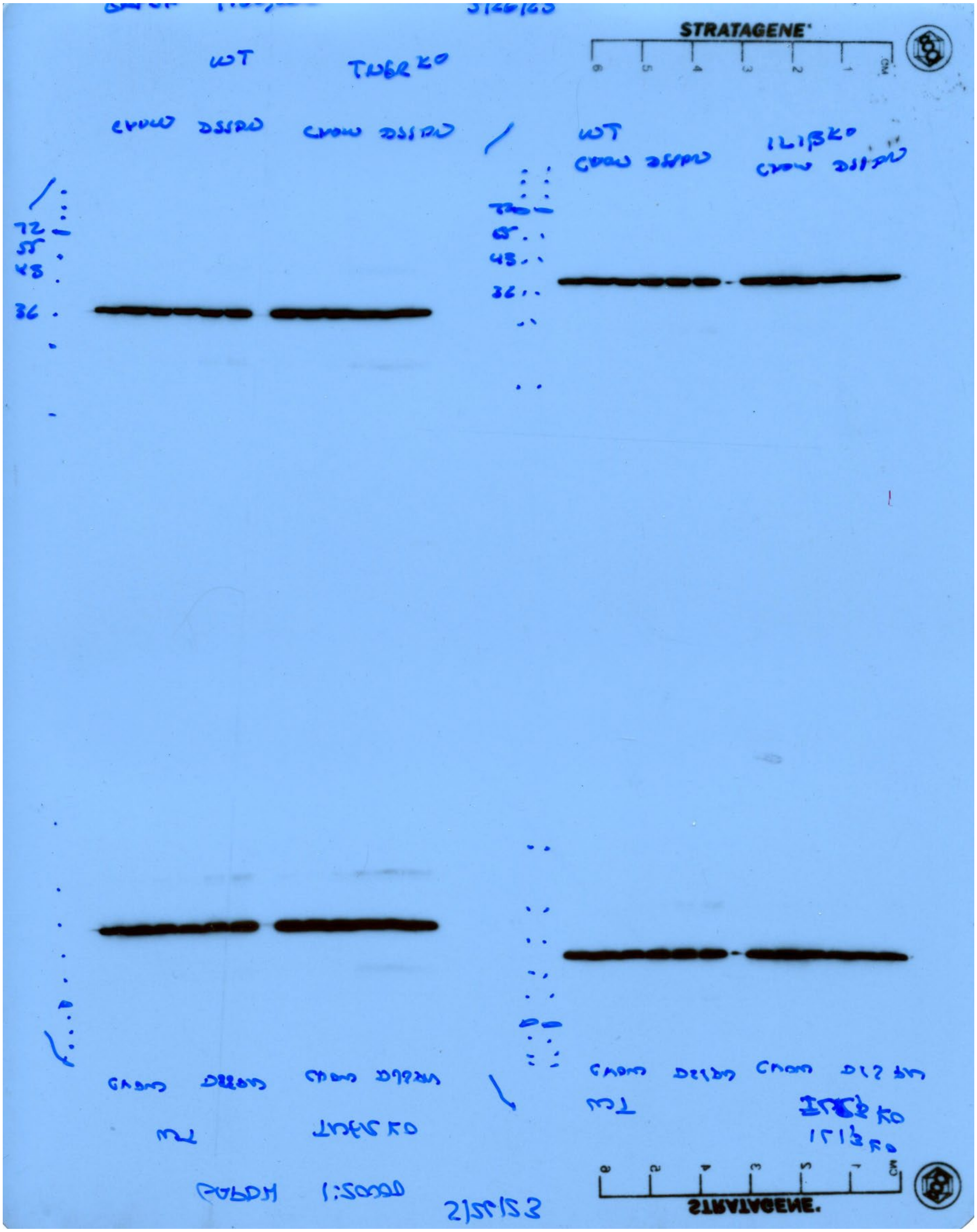
STRATAGENE
WT
TNFR KO
WT
IL1β KO
72
55
45
36
36
GAPDH 1:5000

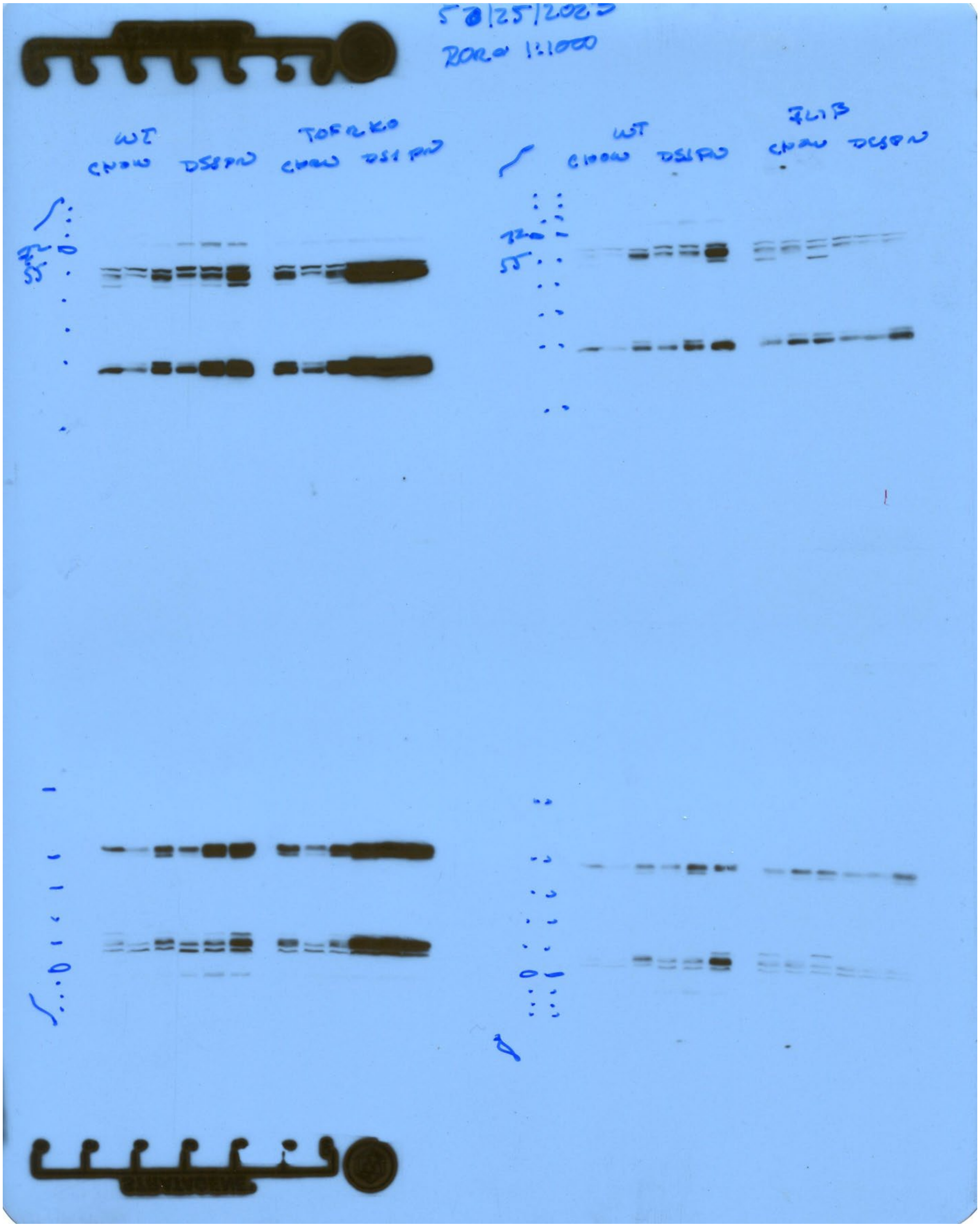
RORα 1:1000
WT
CHOW
WT
55

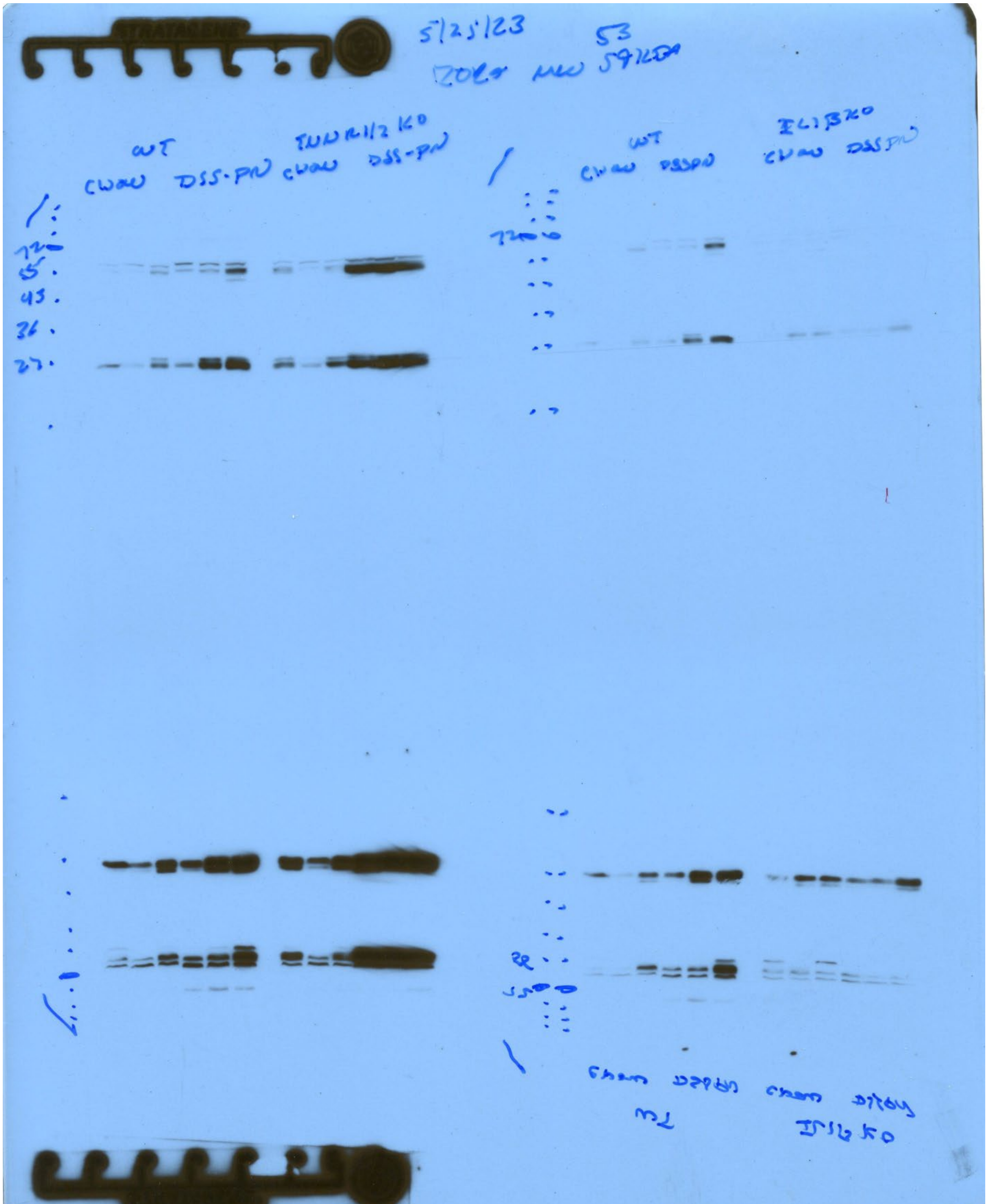
5/25/23
53
WT
chow
DSS-PN
72
55
45
36
27

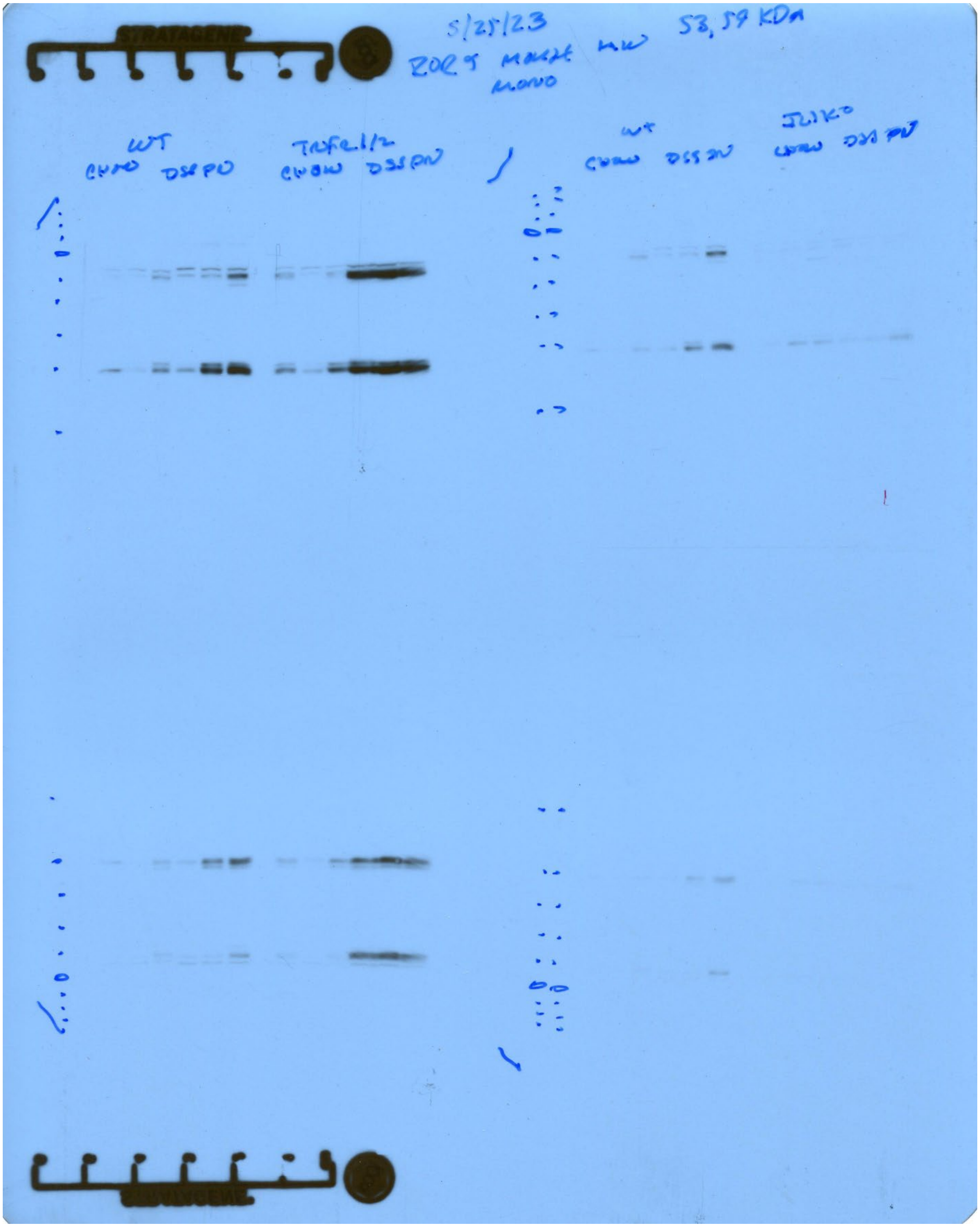
5/25/23
ZOR9 Marker MW 53, 59 KDa
Mono
WT
Chow DSS PD
Tnfr1/2
Chow DSS PD
wt
Chow DSS PD
JLKO
Chow DSS PD

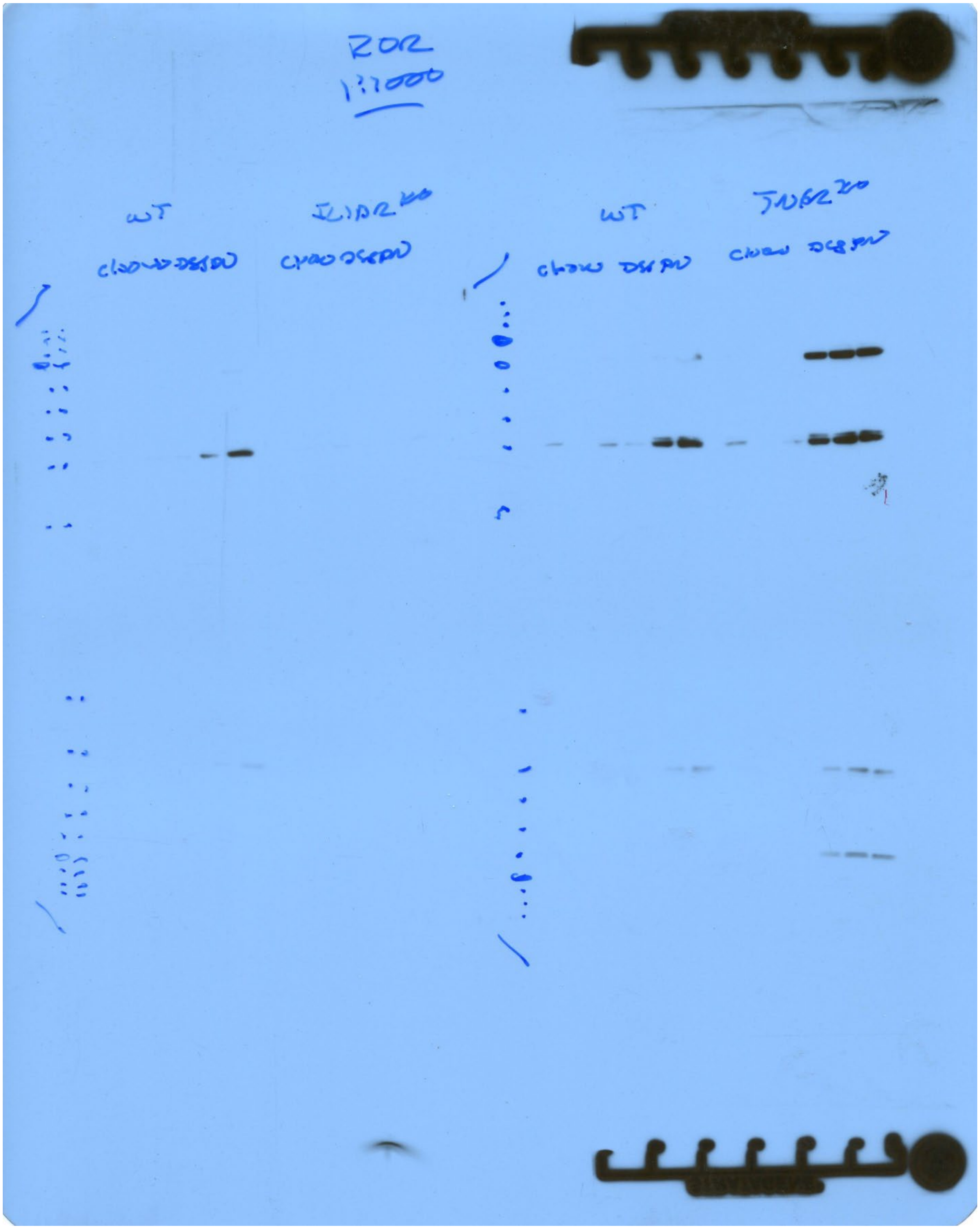

ROR
WT
WT

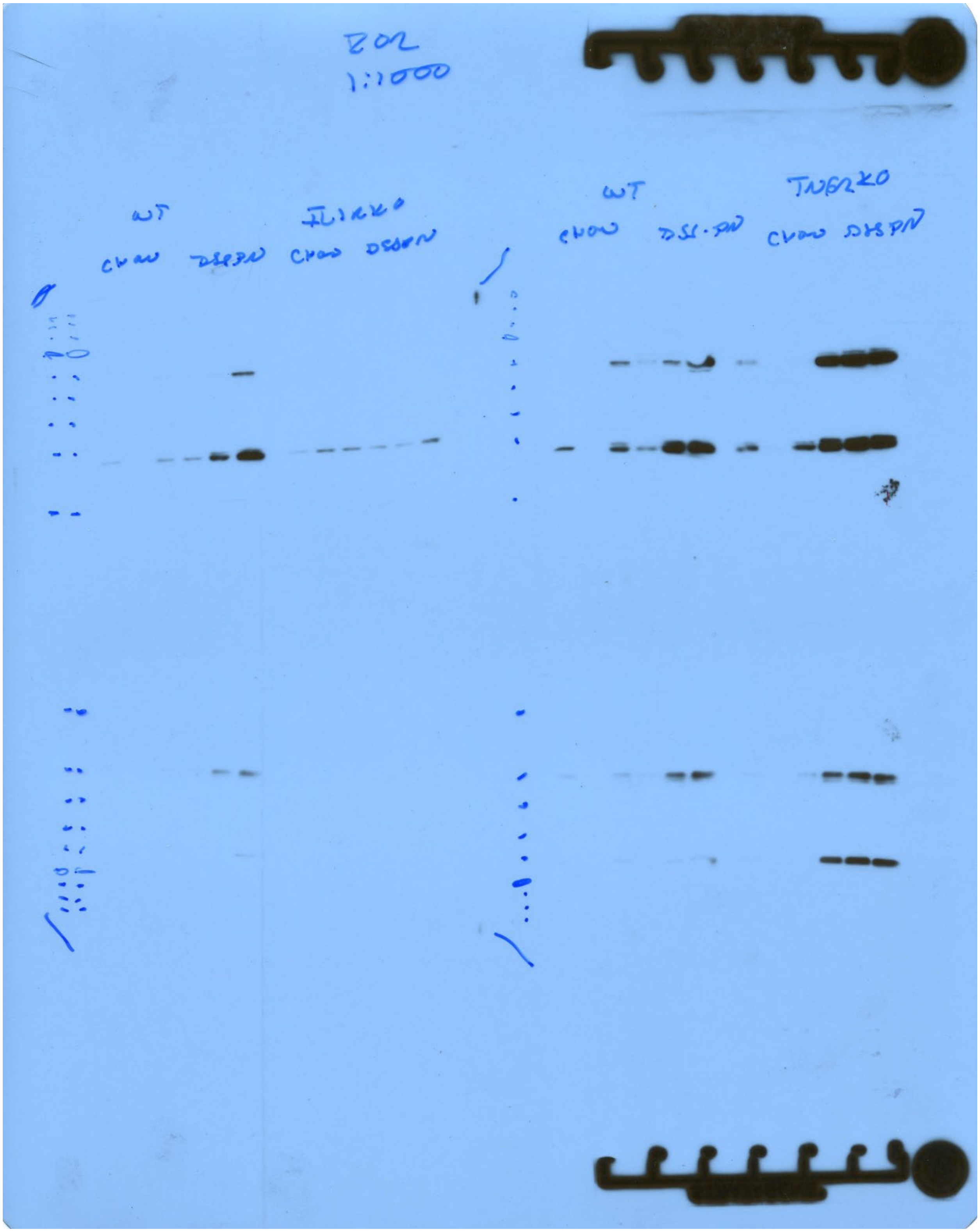
1:1000
WT
CHOW
WT
CHOW

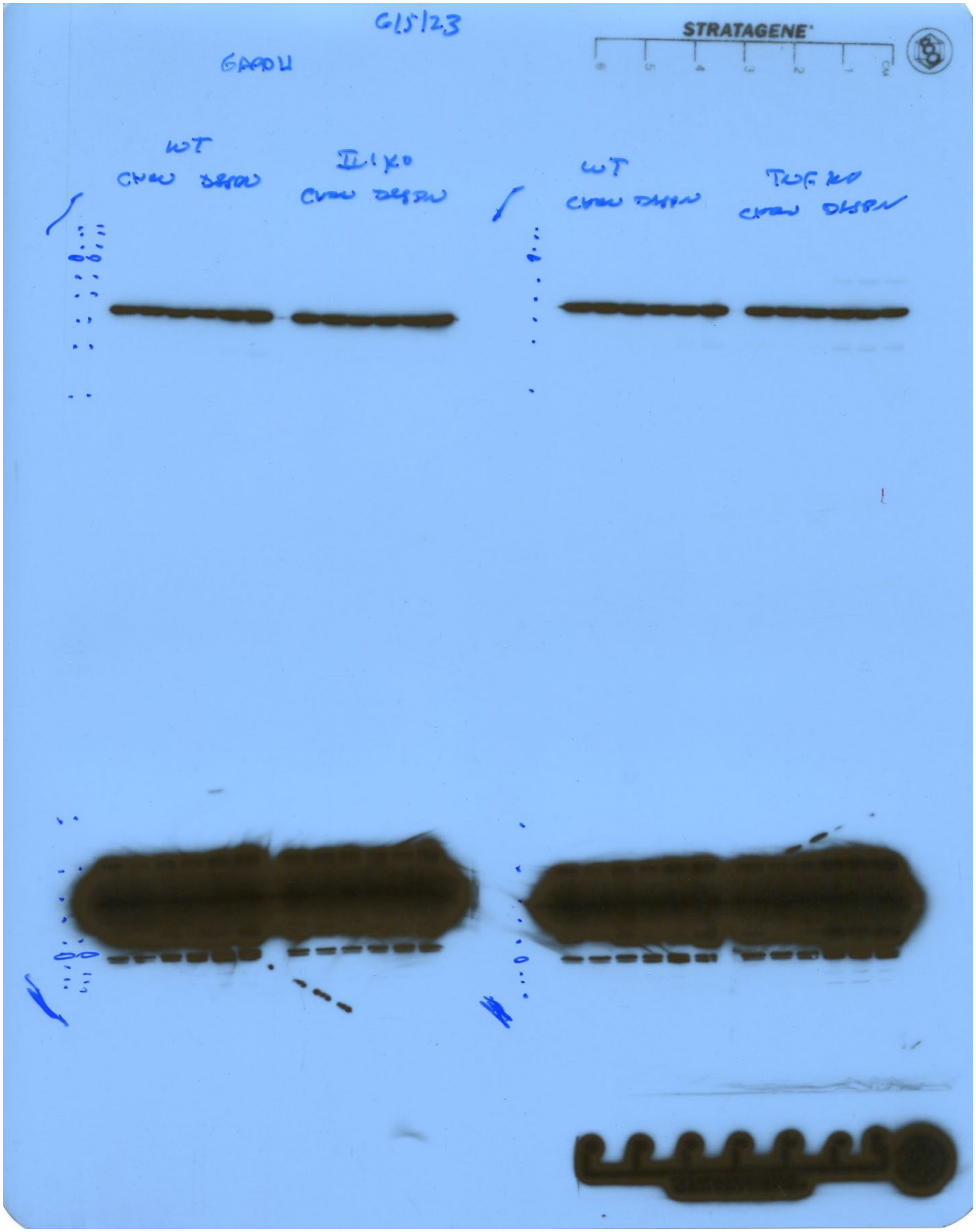
6/5/23
GAPDH
STRATAGENE
WT
WT

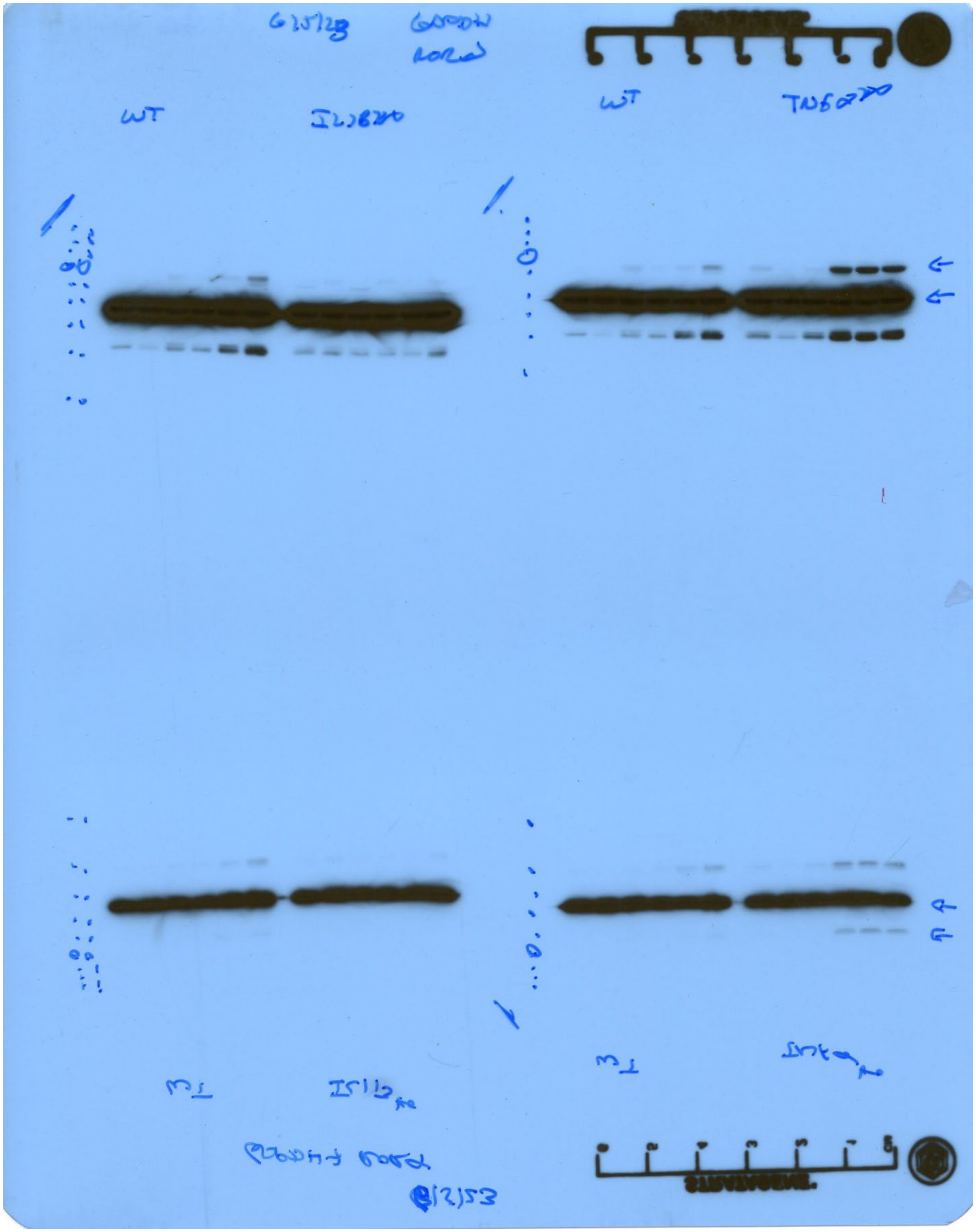
WT
WT

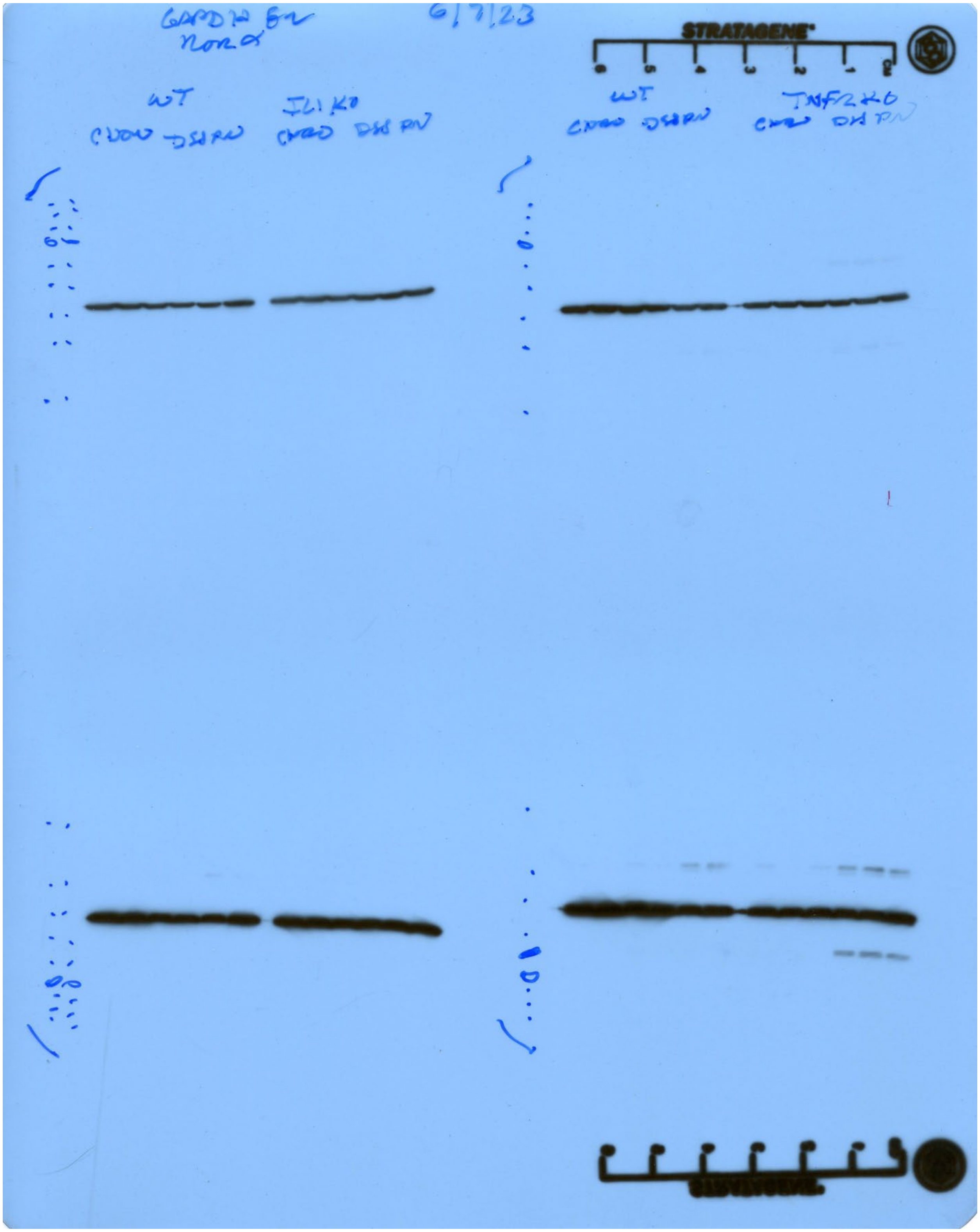
6/7/23
STRATAGENE
WT
WT
TNFR2KO

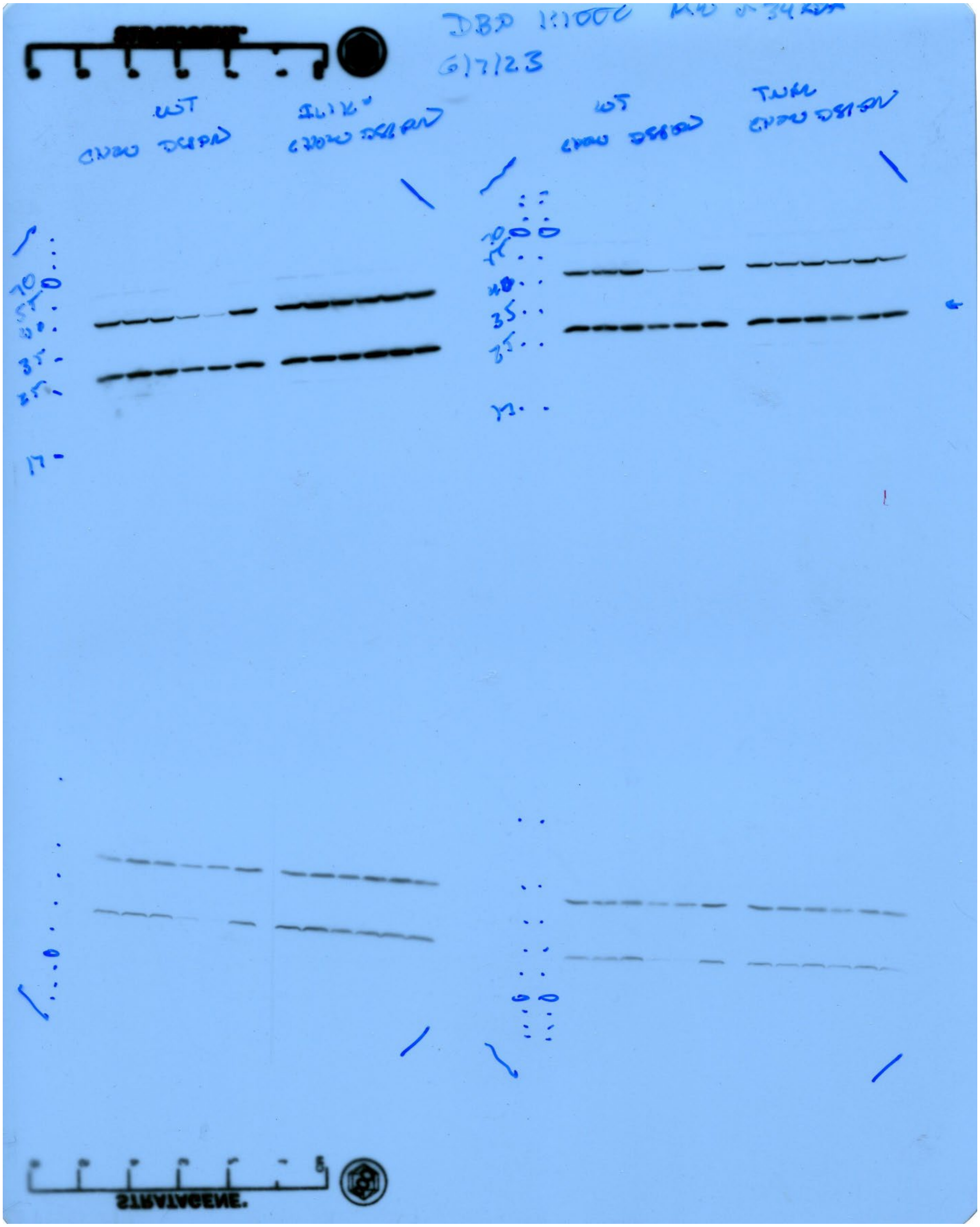
6/7/23
WT
WT
STRATAGENE.

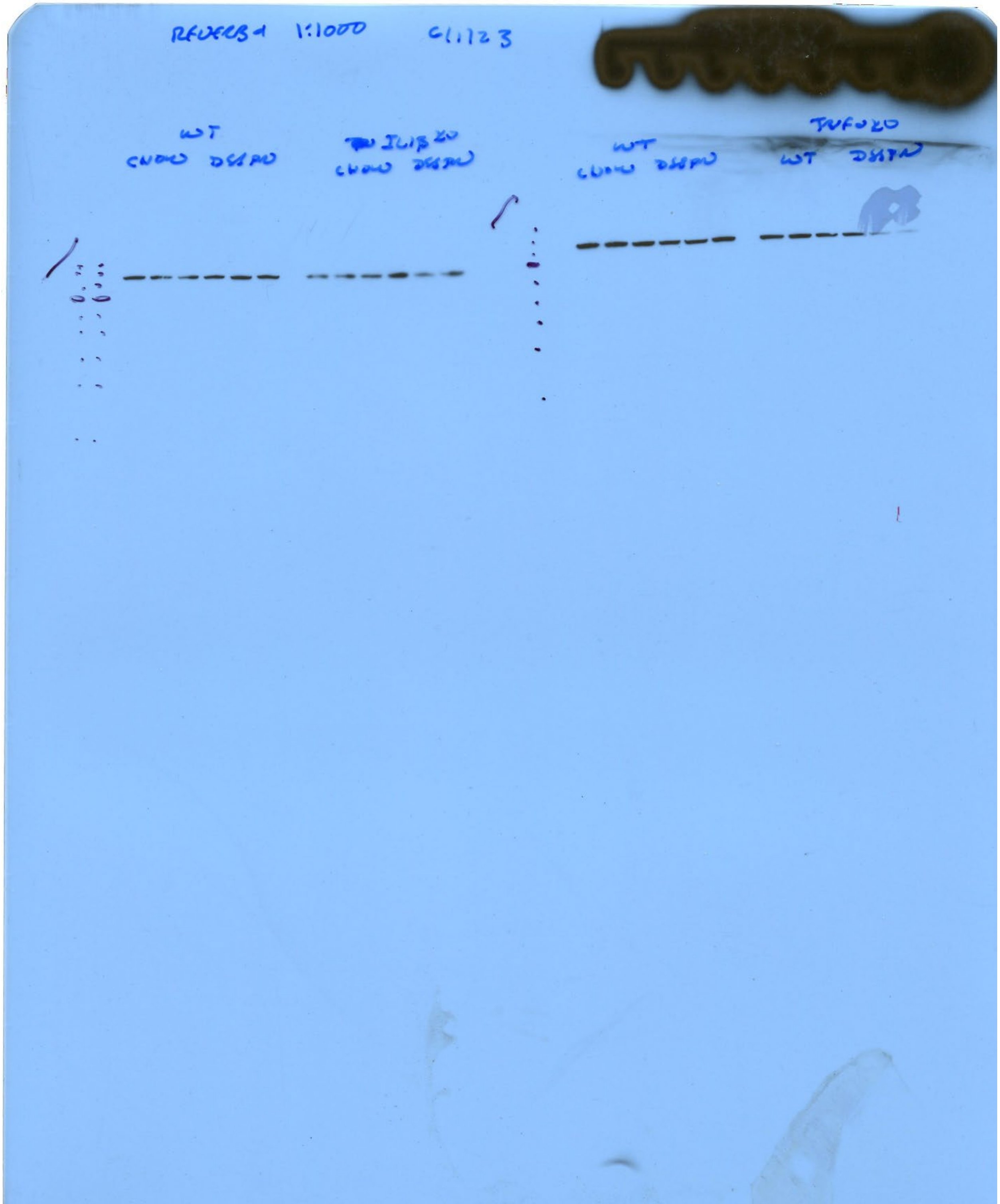
REVERBα 1:1000 6/1/23
WT
CHOW
IL1β KO
CHOW
WT
CHOW
TNFα KO
WT

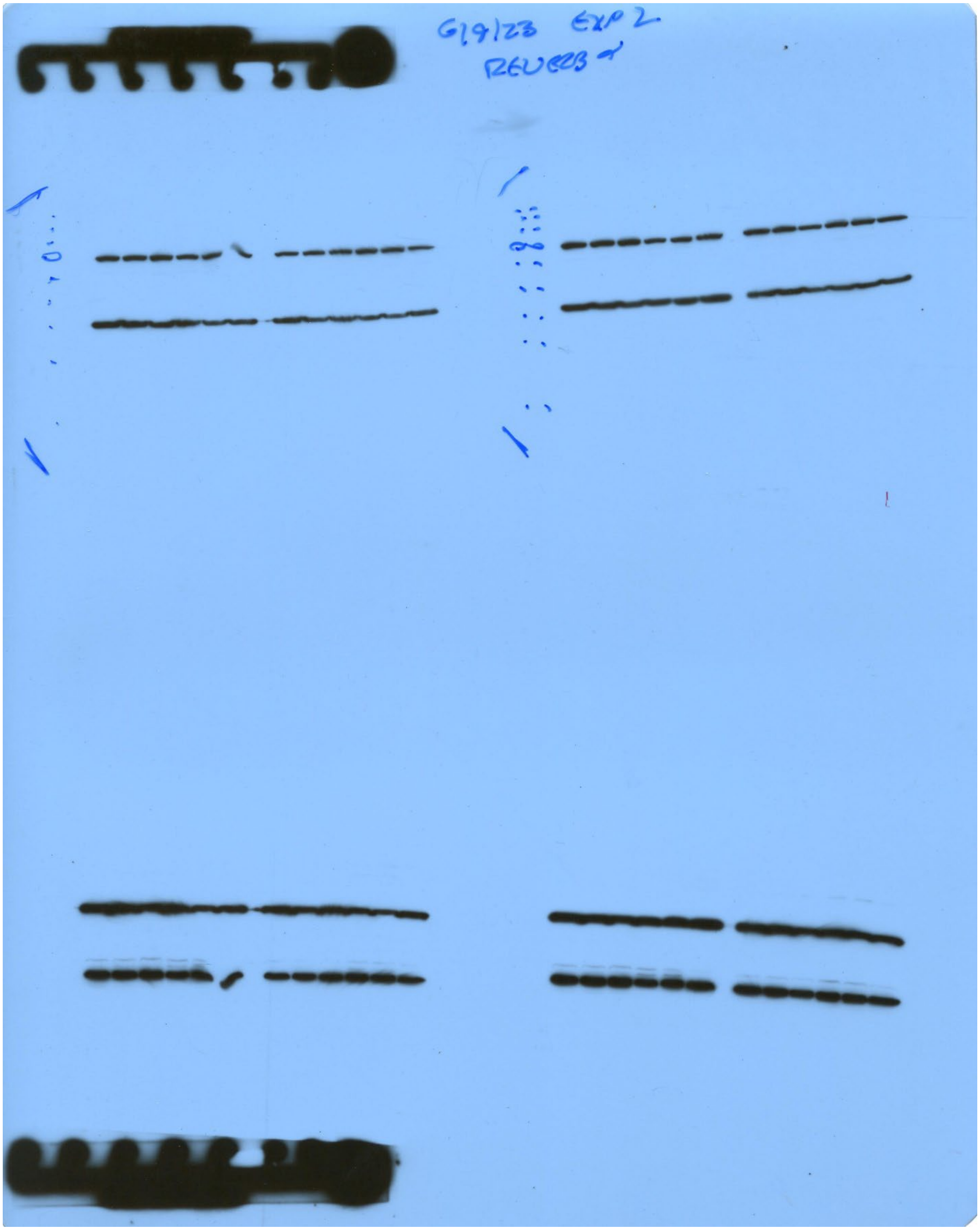
6/9/23 EXP 2
REVERB α

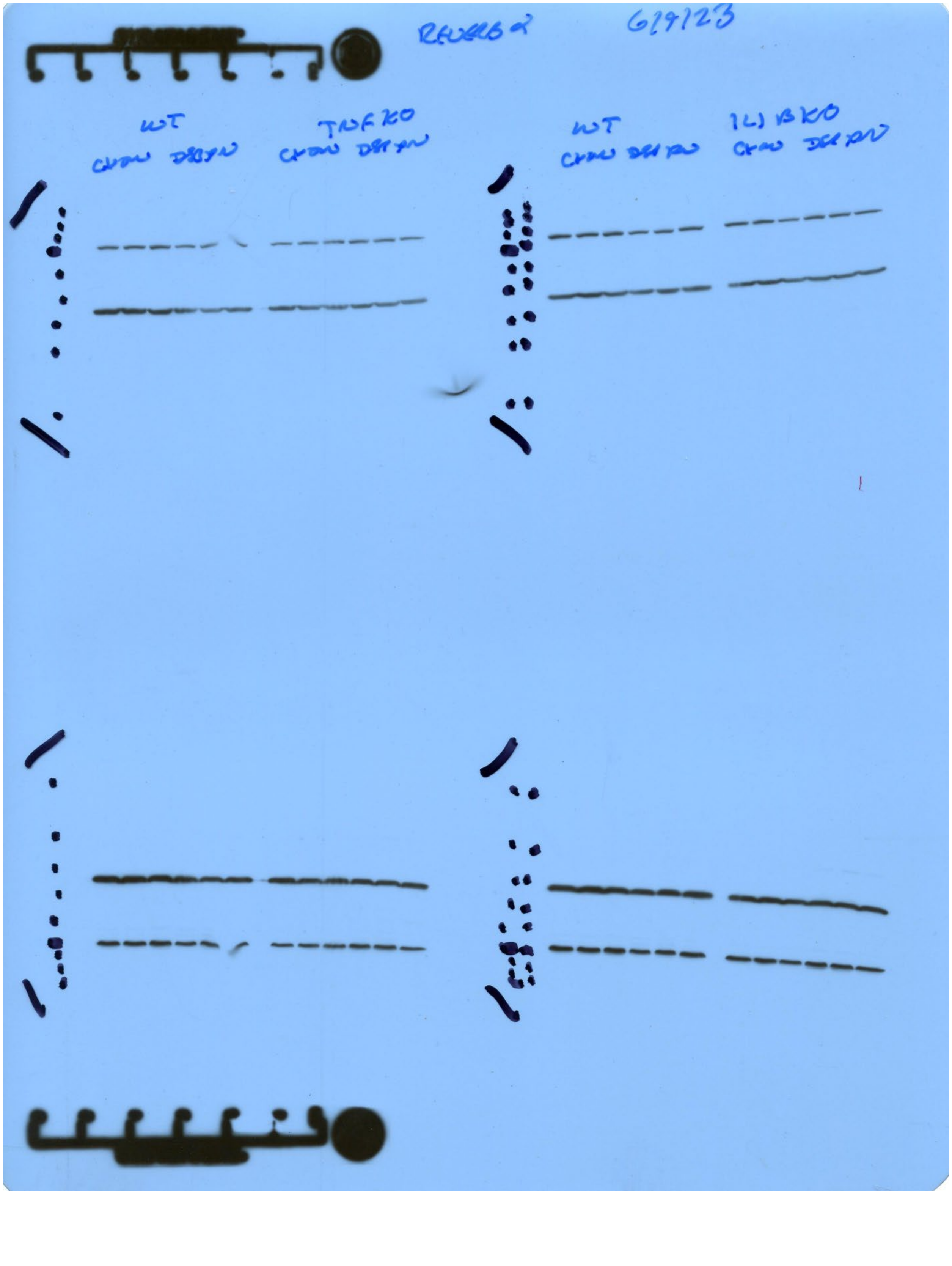
REVERBα
6/9/23
WT
TNF KO
WT
IL1B KO

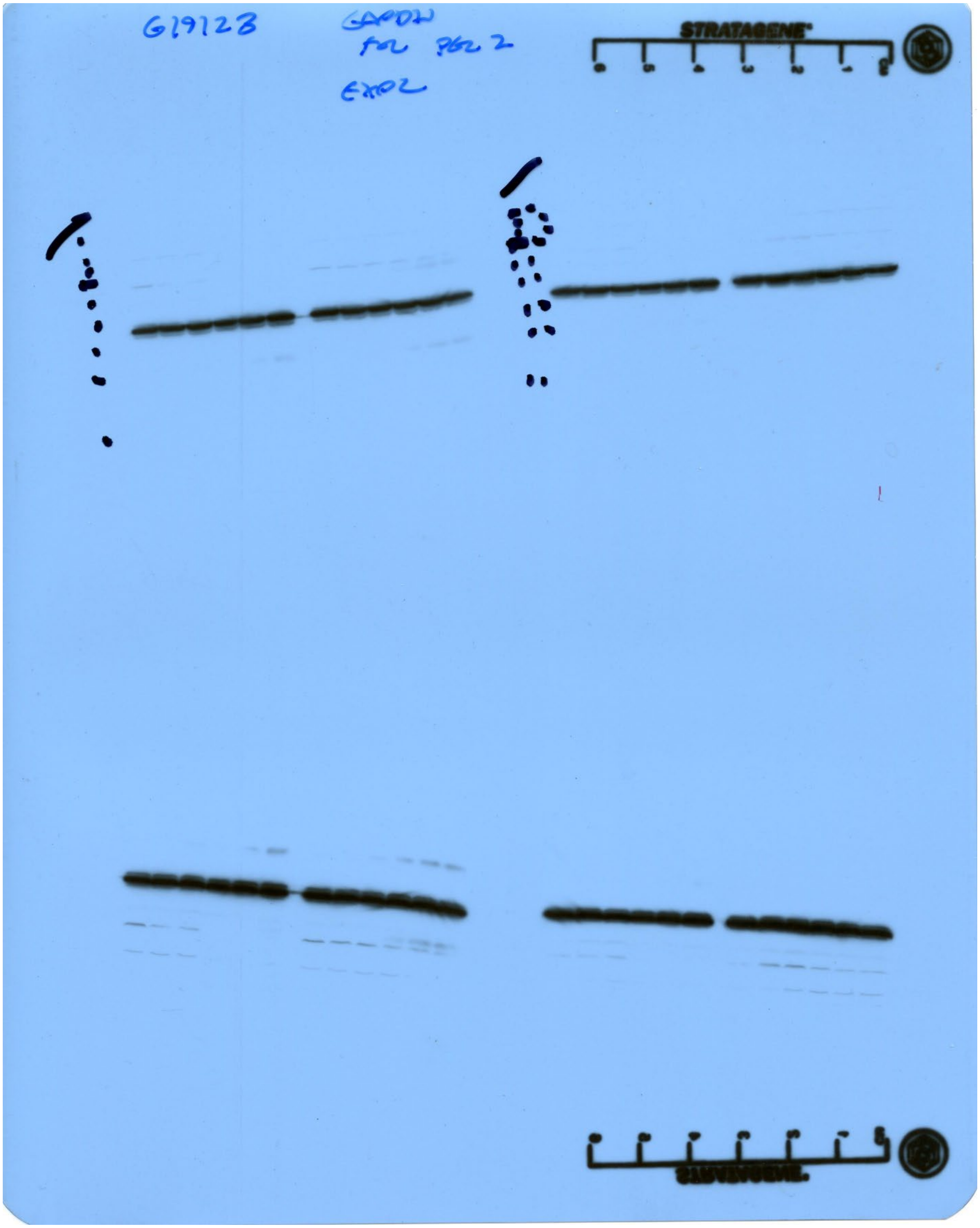
GAPDH
EXP2
STRATAGENE

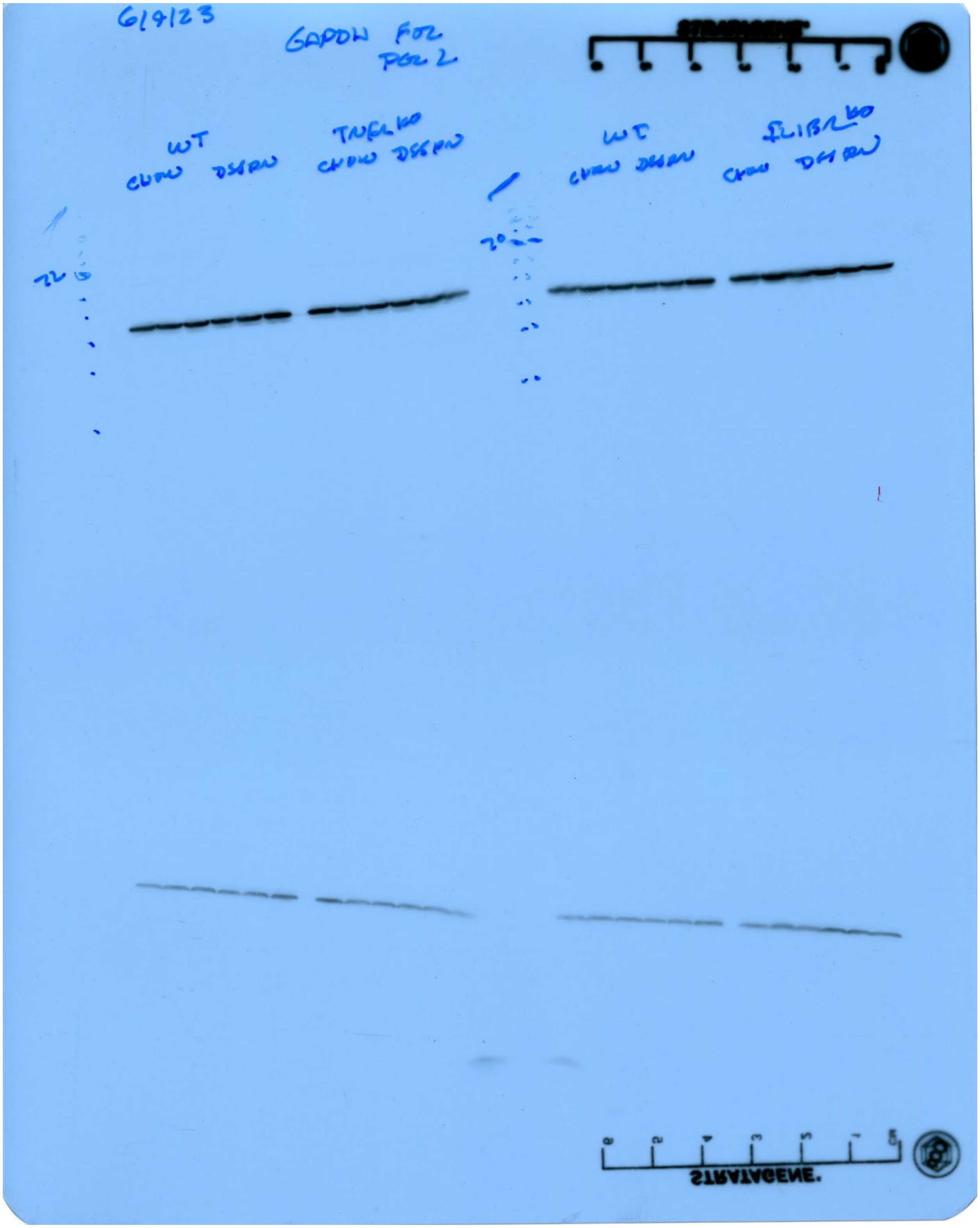
6/9/23
WT
WT
STRATAGENE.

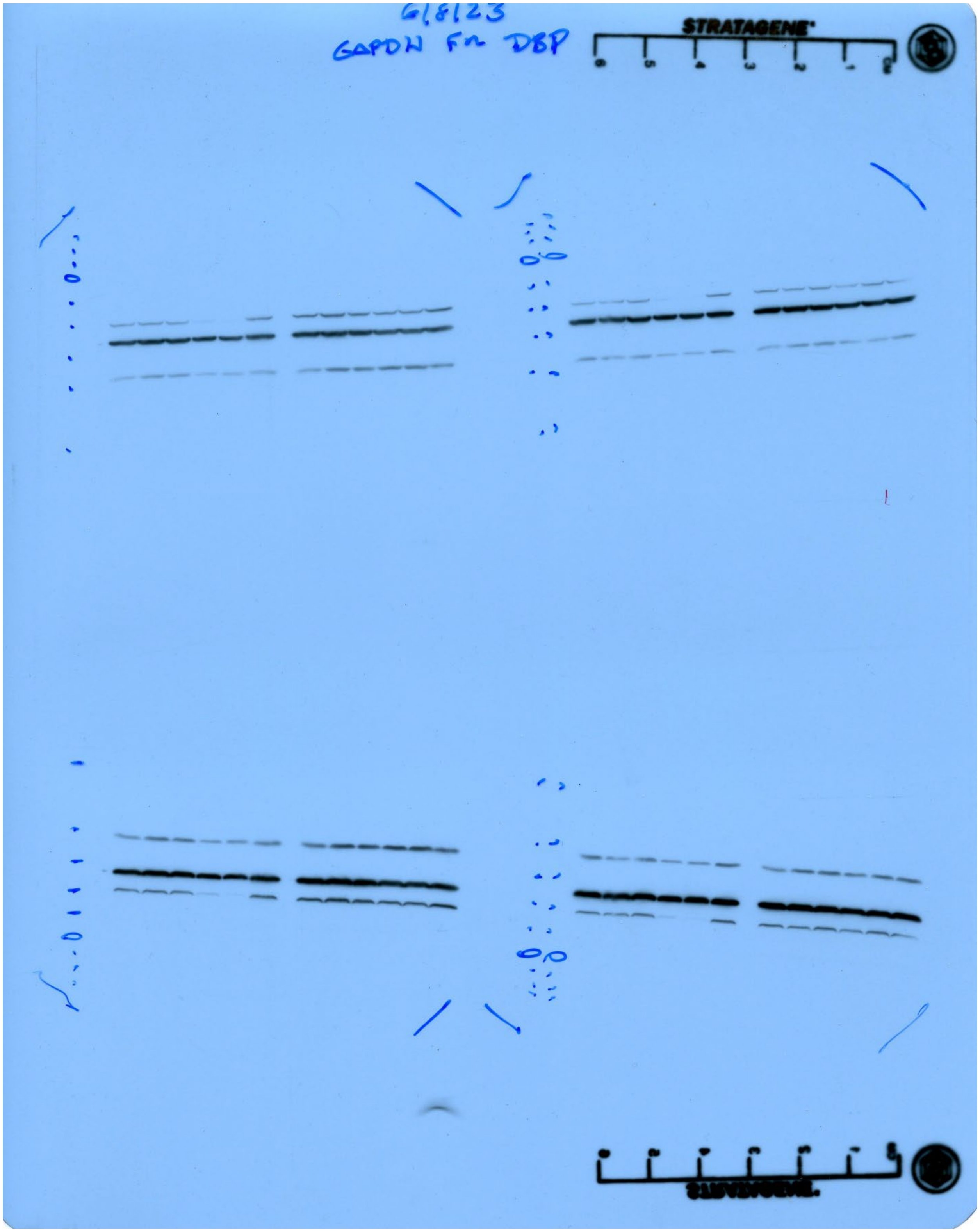
6/8/23
GAPDH Fr DBP
STRATAGENE

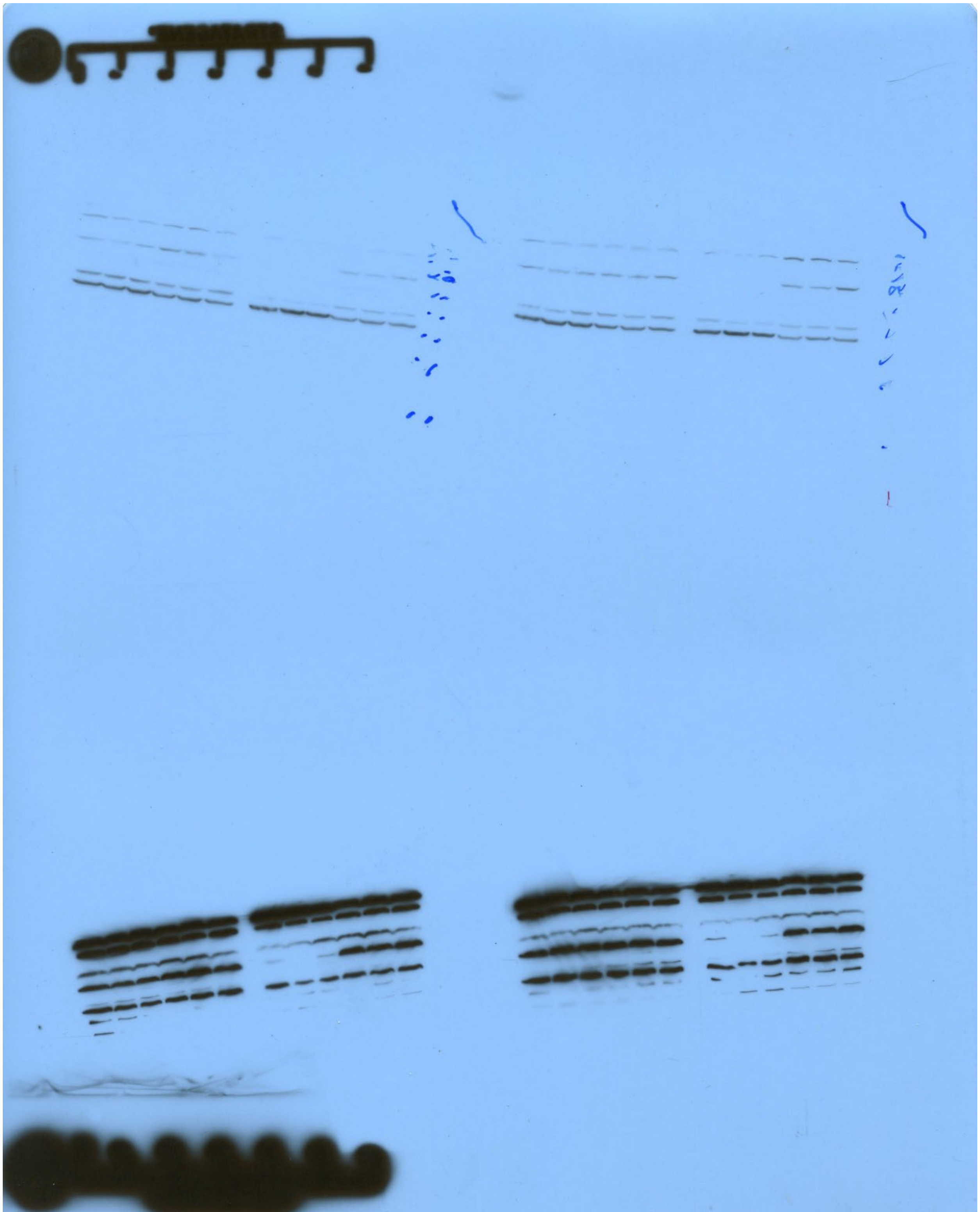

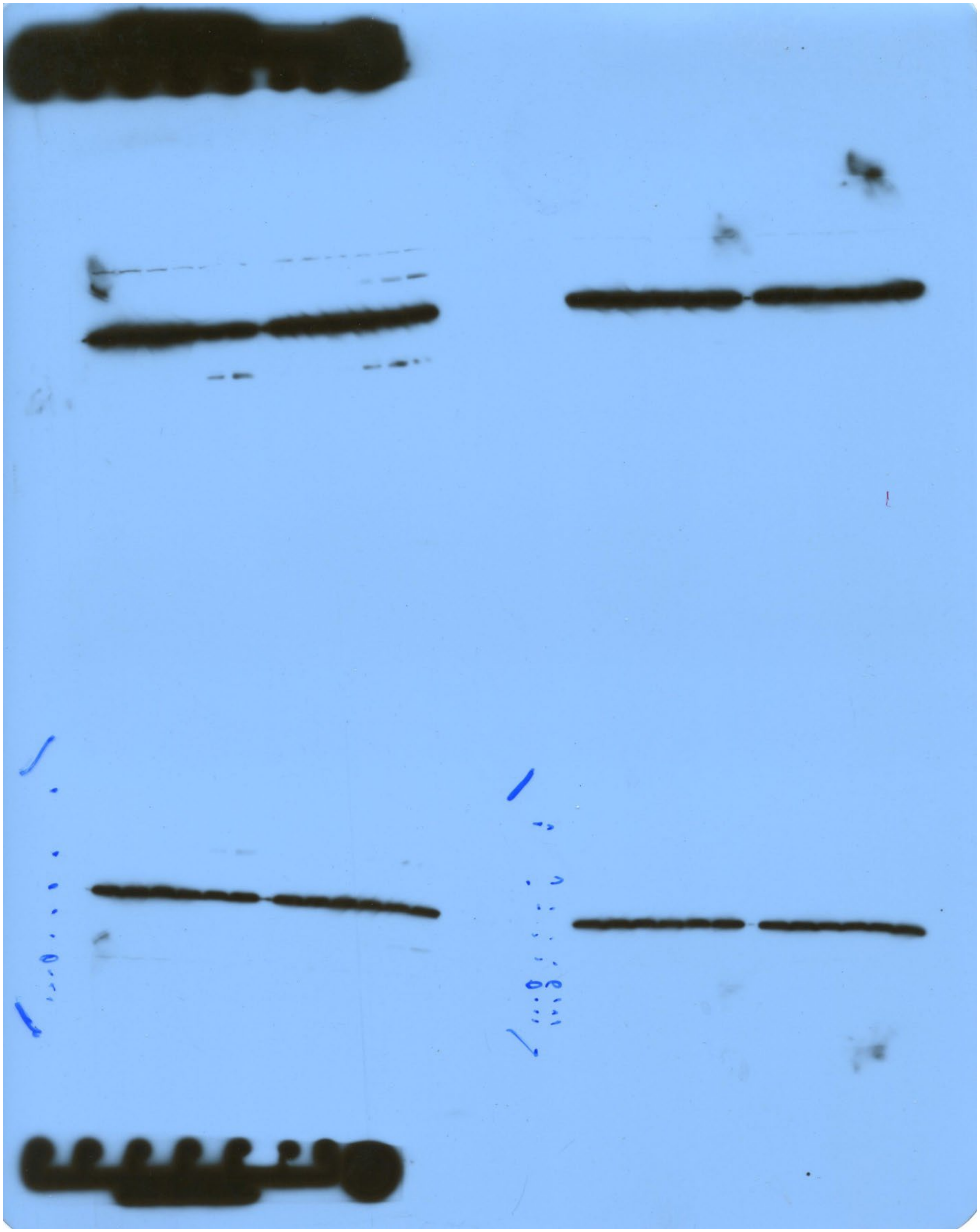

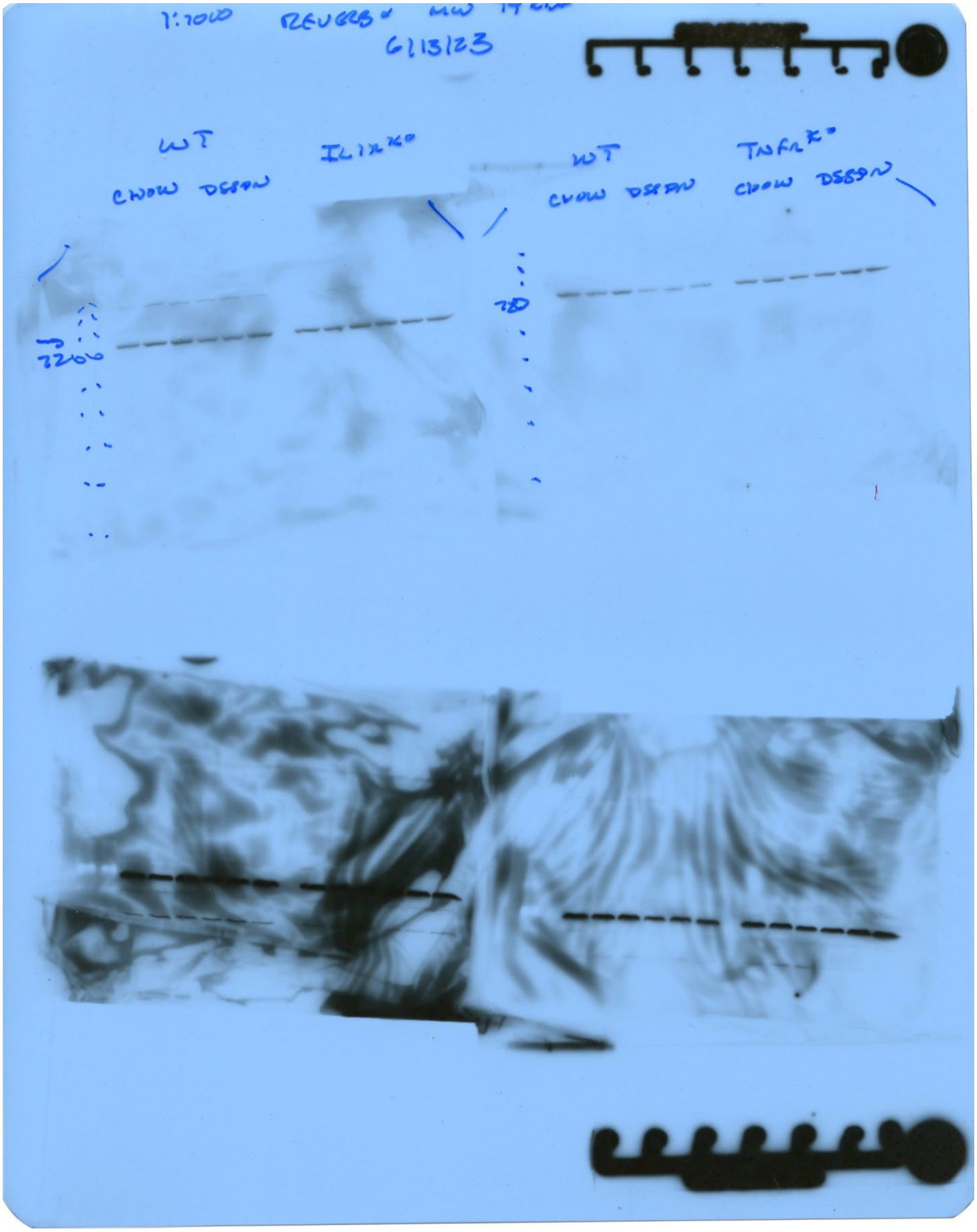
6/13/23
WT
WT

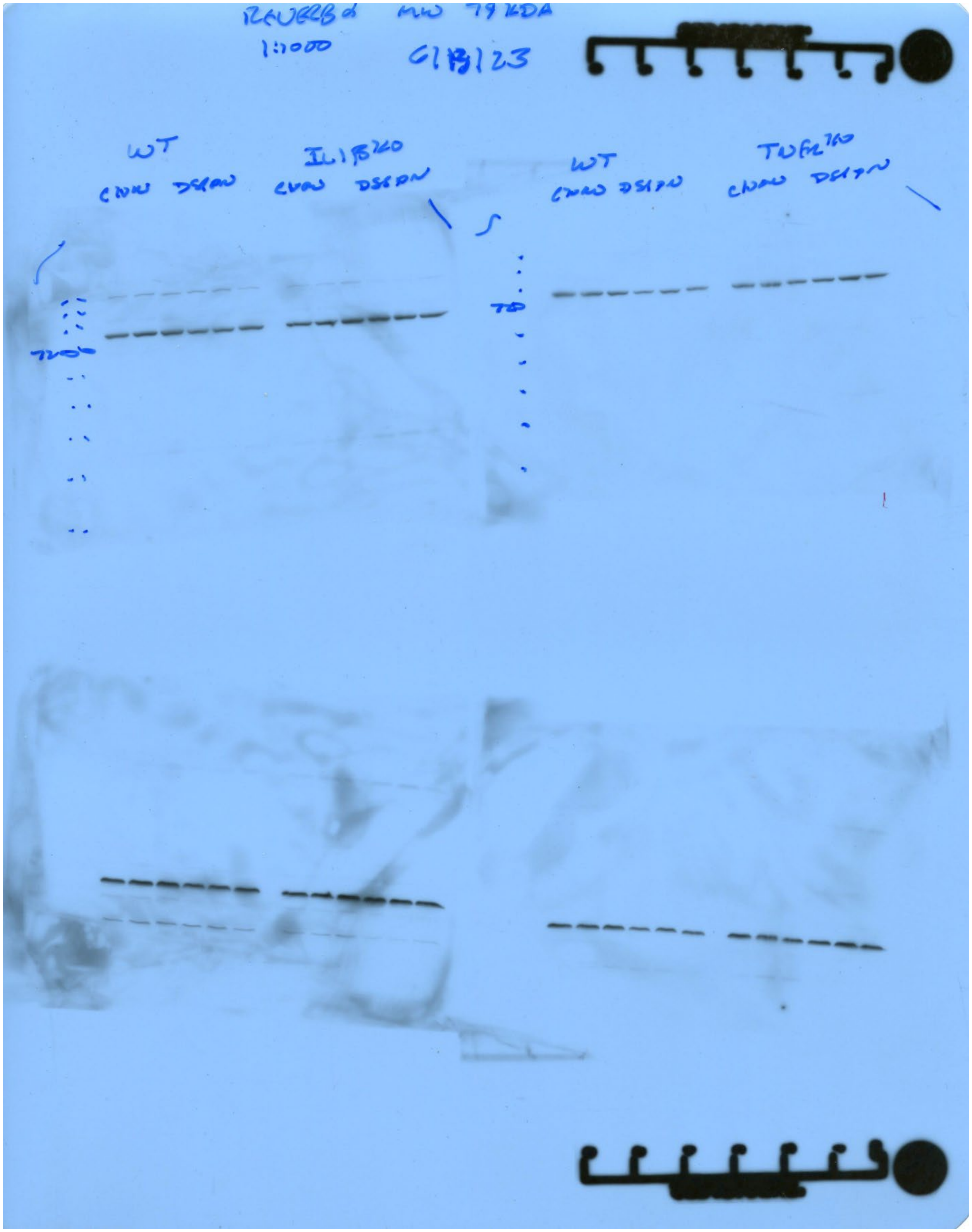
MW 79 KDA
1:1000
6/13/23
WT
WT

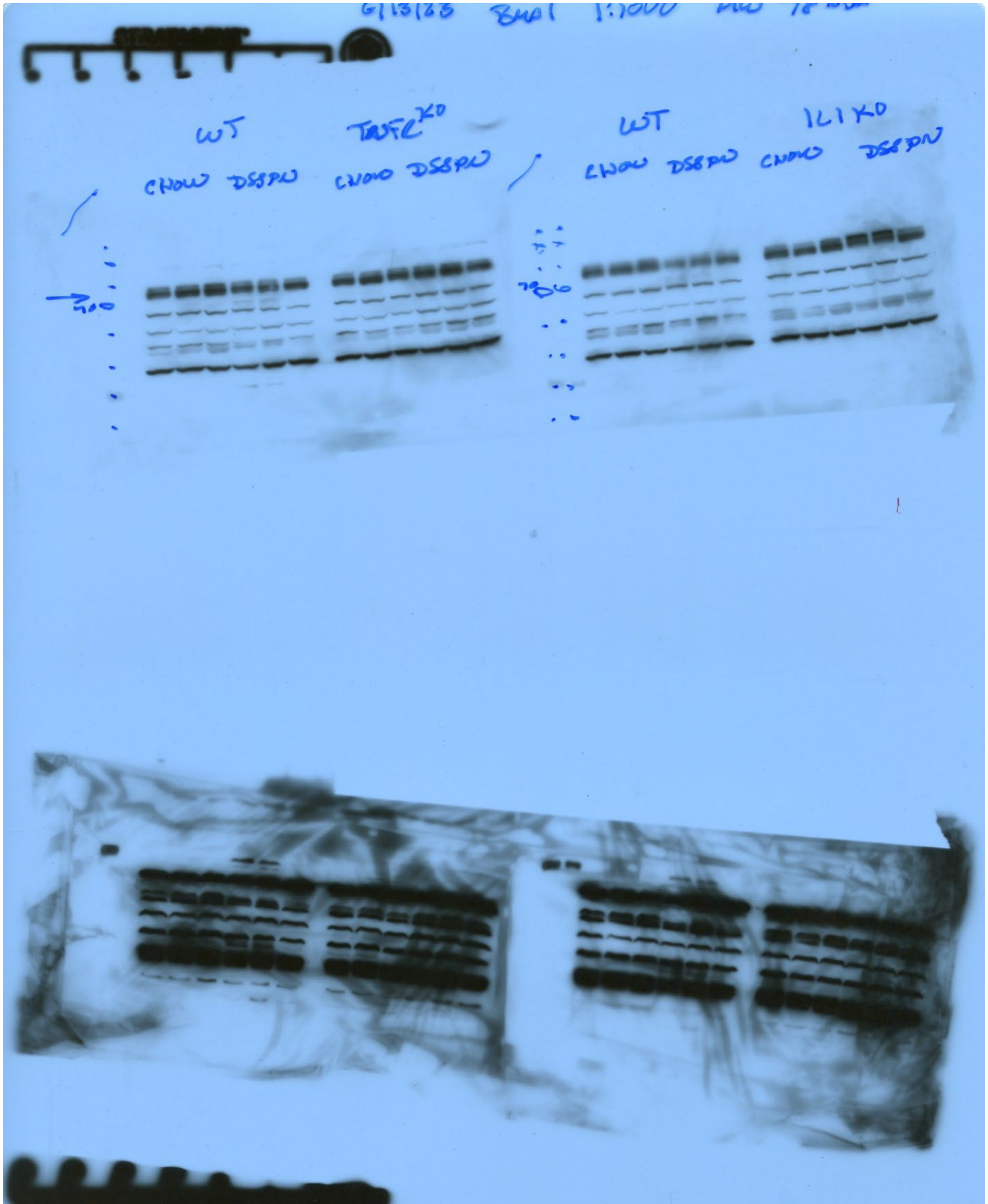
WT
CHOW
WT
IL1 KO

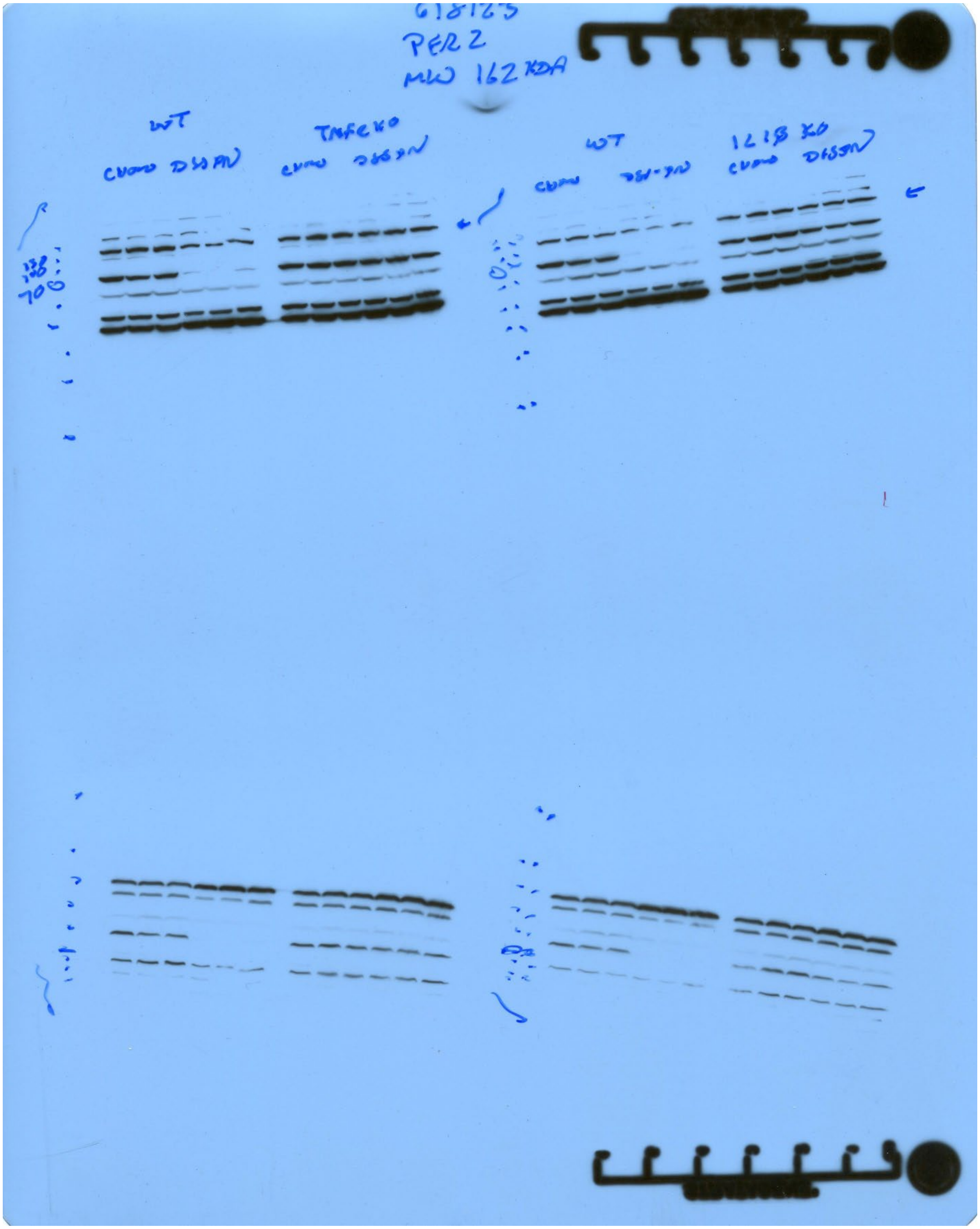
PER2
MW 162 KDA
WT
WT

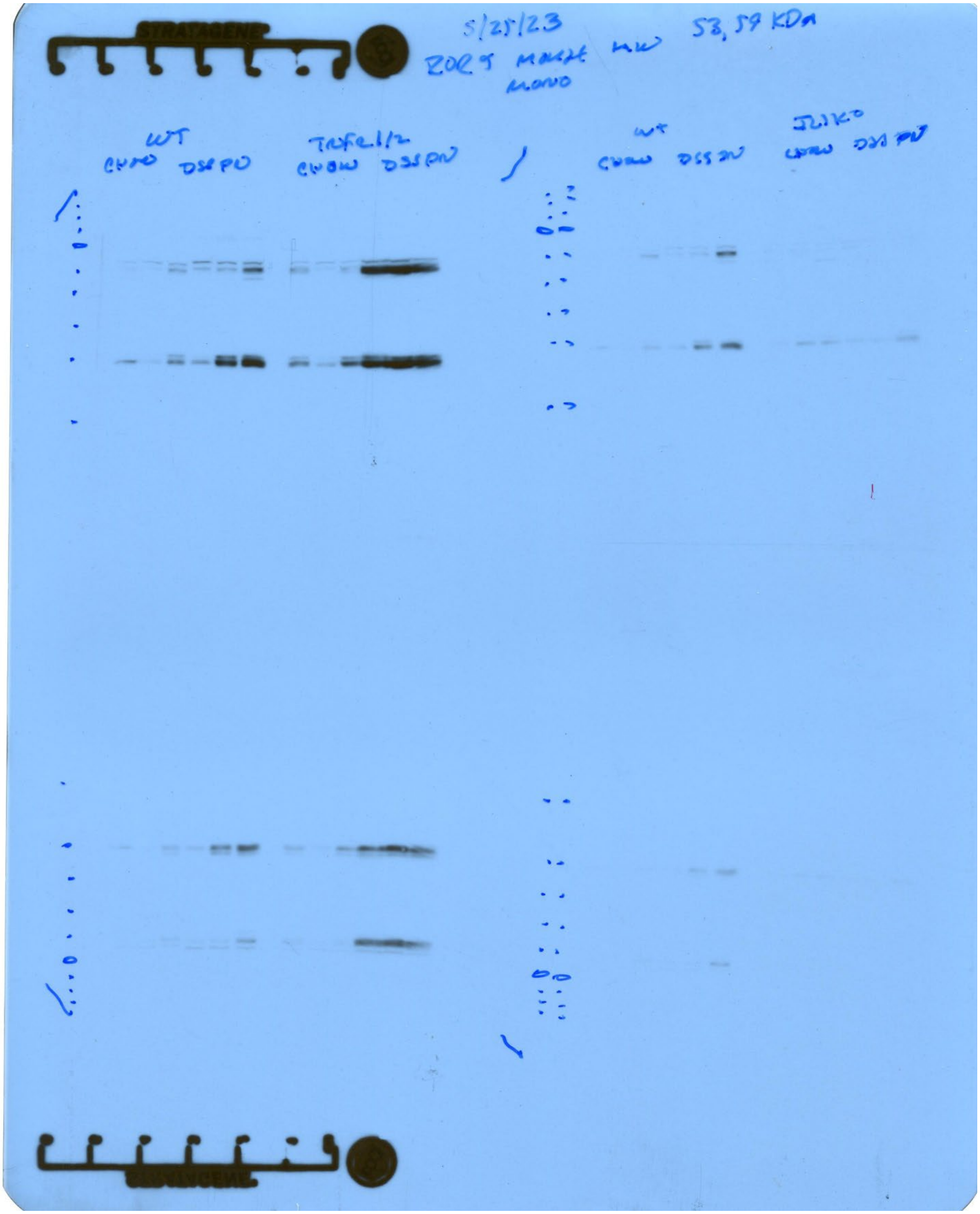
STRATAGENE
5/25/23
RORγ Mouse mono
MW 58, 59 KDa
WT
Chow DSS PW
TNFR1/2
Chow DSS PW
WT
Chow DSS PW
IL1KO
Chow DSS PW
STRATAGENE

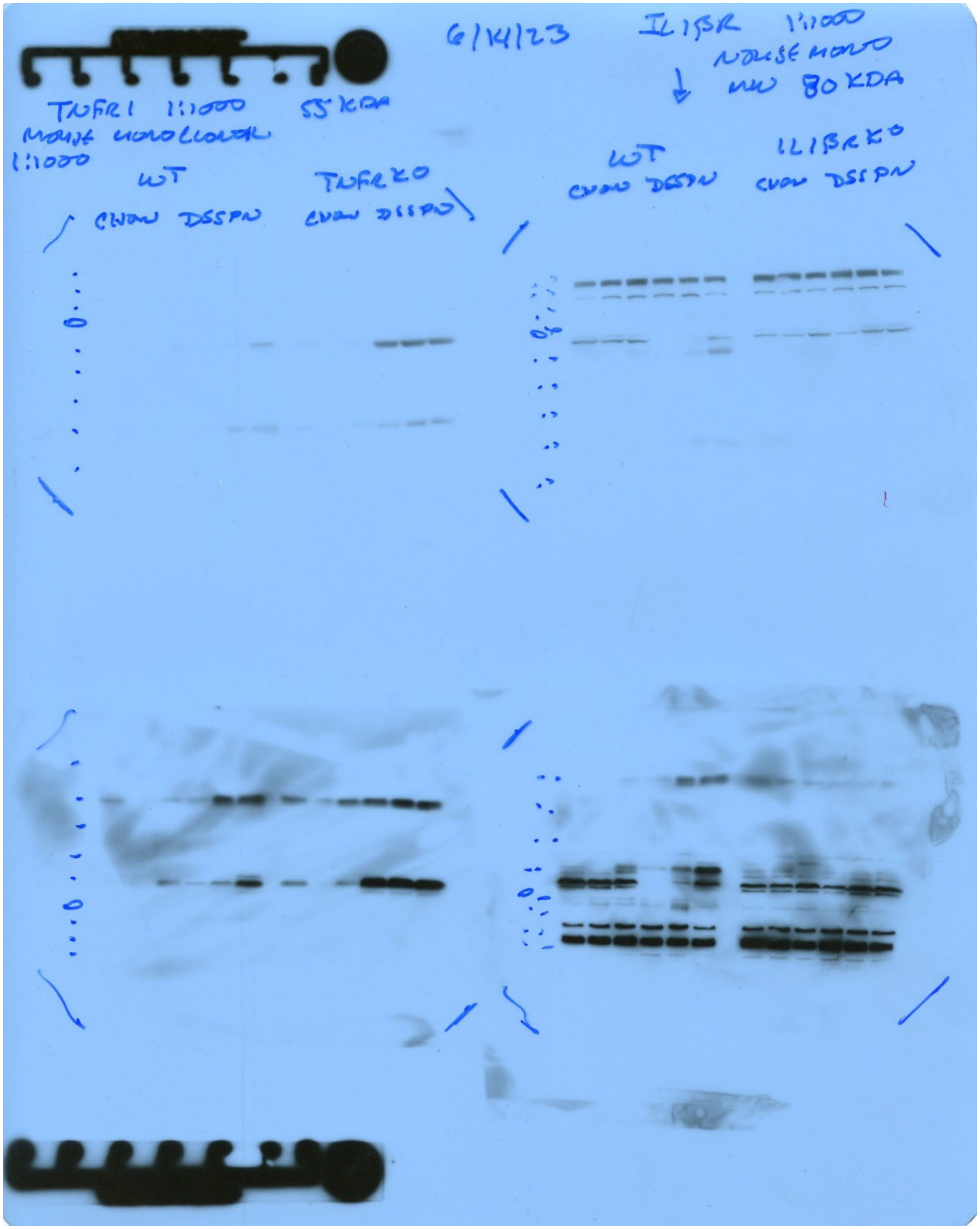
6/14/23
IL1βR 1:1000
MOUSE MONO
MW 80 KDA
TNFR1 1:1000 55 KDA
MOUSE MONOCLONAL
1:1000
WT
TNFRKO
CHOW DSSPN
CHOW DSSPN
WT
IL1βRKO
CHOW DSSPN
CHOW DSSPN

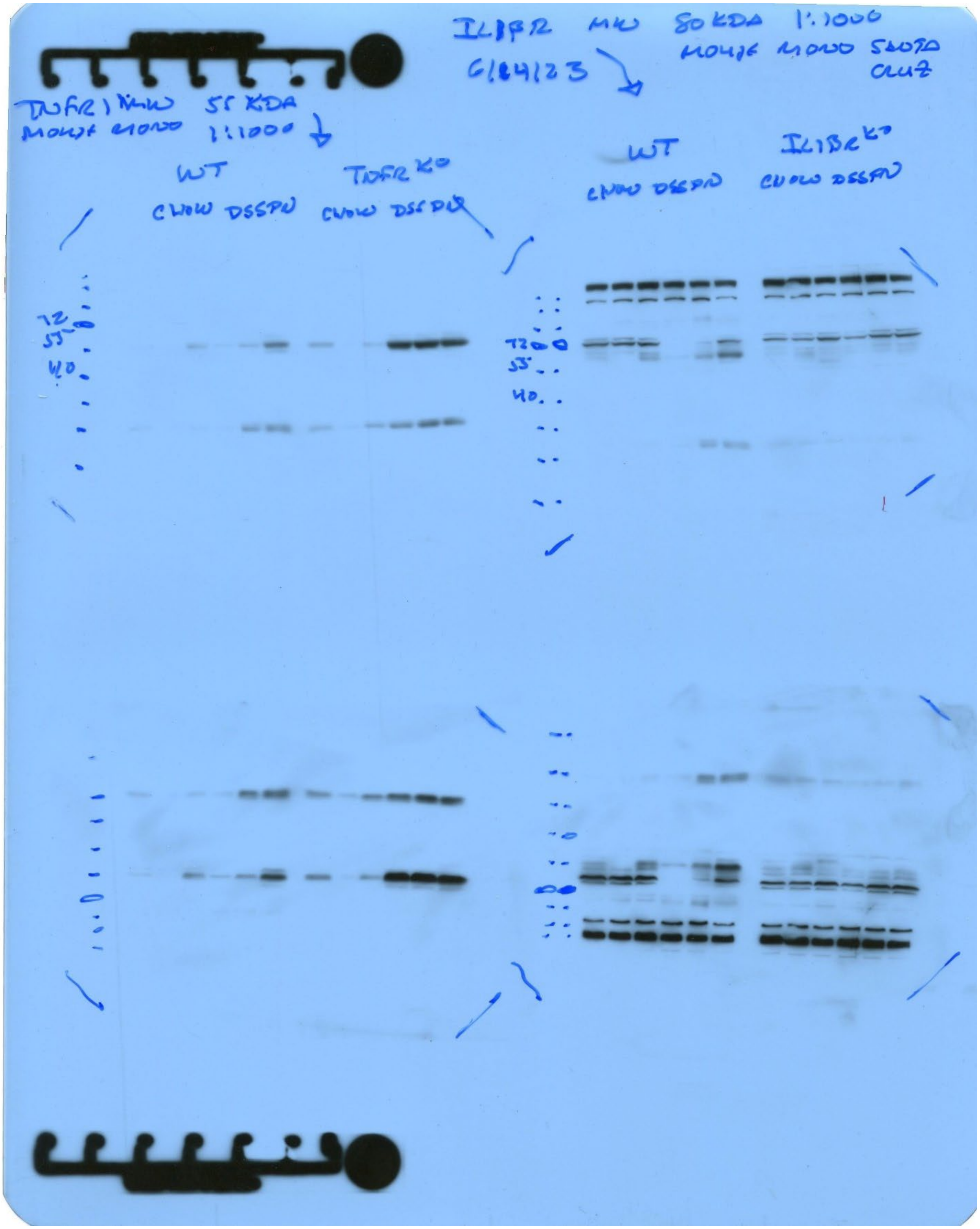

TNFR1 MW 55 KDA
Mouse mono 1:1000
WT
TNFR KO
CHOW DSSPD
CHOW DSSPD
IL1βR MW 80 KDA 1:1000
Mouse mono Santa Cruz
WT
IL1βR KO
CHOW DSSPD
CHOW DSSPD
72
55
40

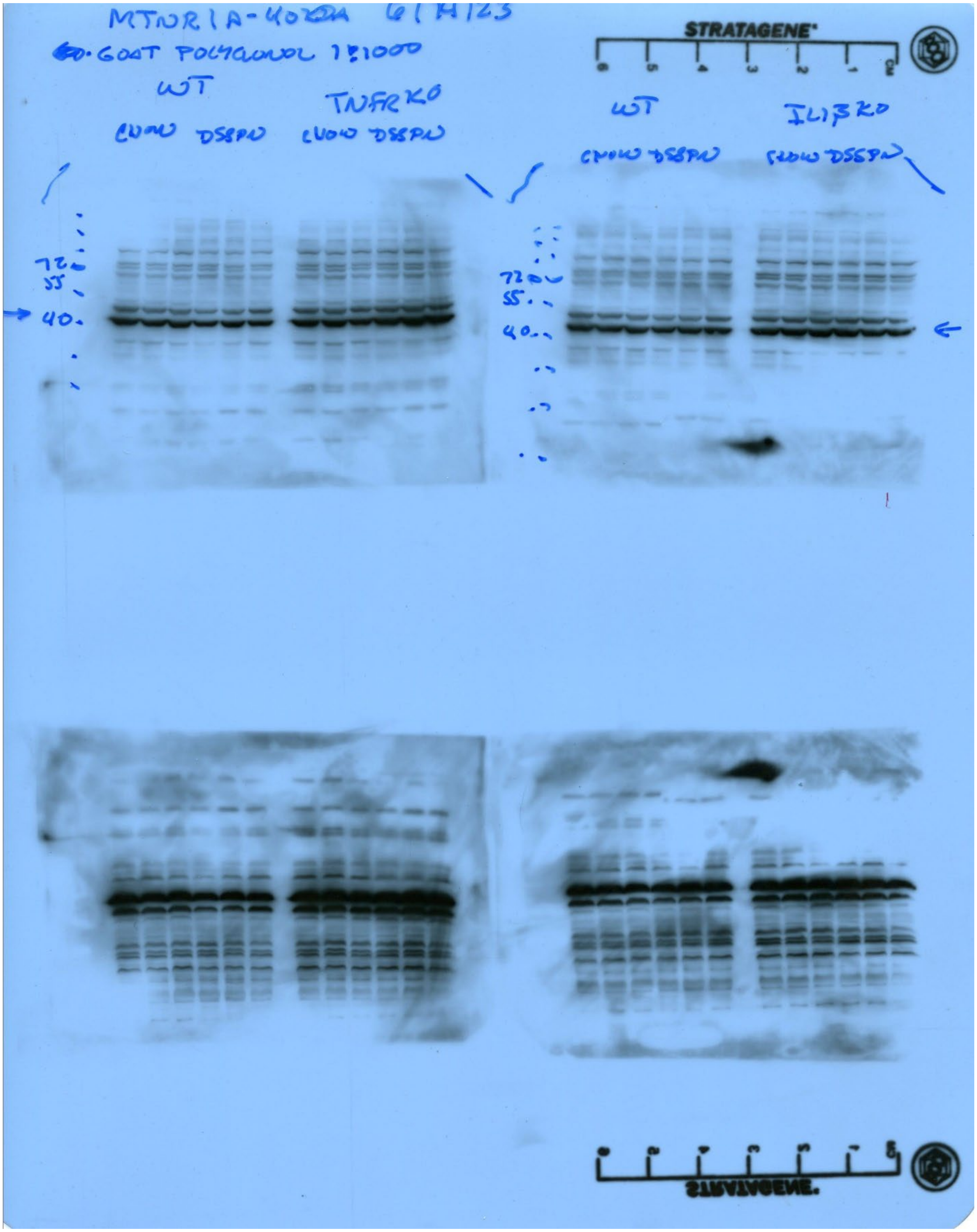
MTNR1A-40kDa 6/14/23
GOAT POLYCLONAL 1:1000
WT
TNFR KO
CHOW DSSPN CHOW DSSPN
72
55
40
STRATAGENE
WT
IL1β KO
CHOW DSSPN CHOW DSSPN
72
55
40
STRATAGENE

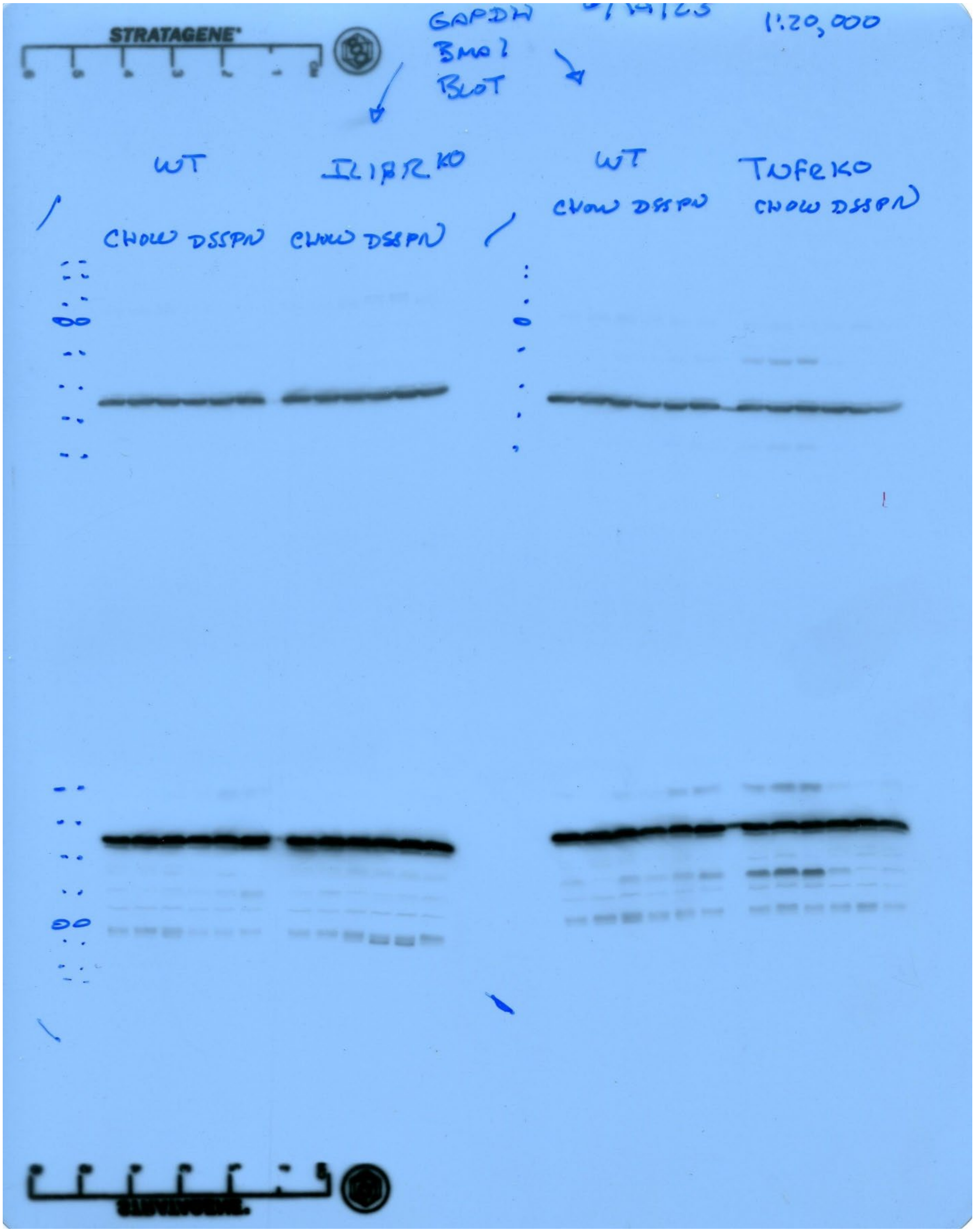
STRATAGENE
GAPDH
Bmal
Blot
1:20,000
WT
IL1βR KO
CHOW DSSPN
CHOW DSSPN
WT
TNFR KO
CHOW DSSPN
CHOW DSSPN

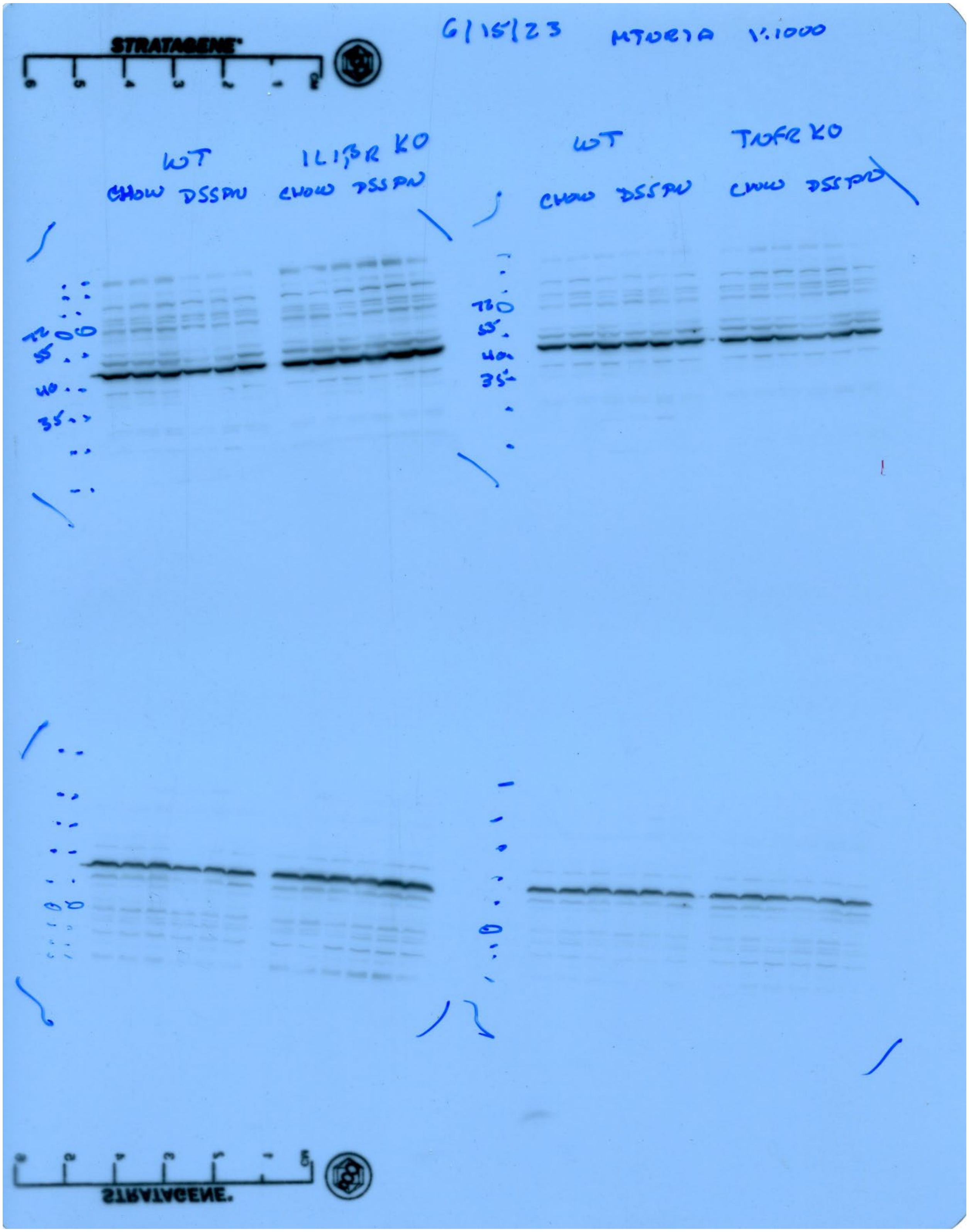
6/15/23
MTOR1A 1:1000
STRATAGENE
WT
IL1βR KO
CHOW DSSPN
CHOW DSSPN
WT
TNFR KO
CHOW DSSPN
CHOW DSSPN
70
55
40
35
STRATAGENE

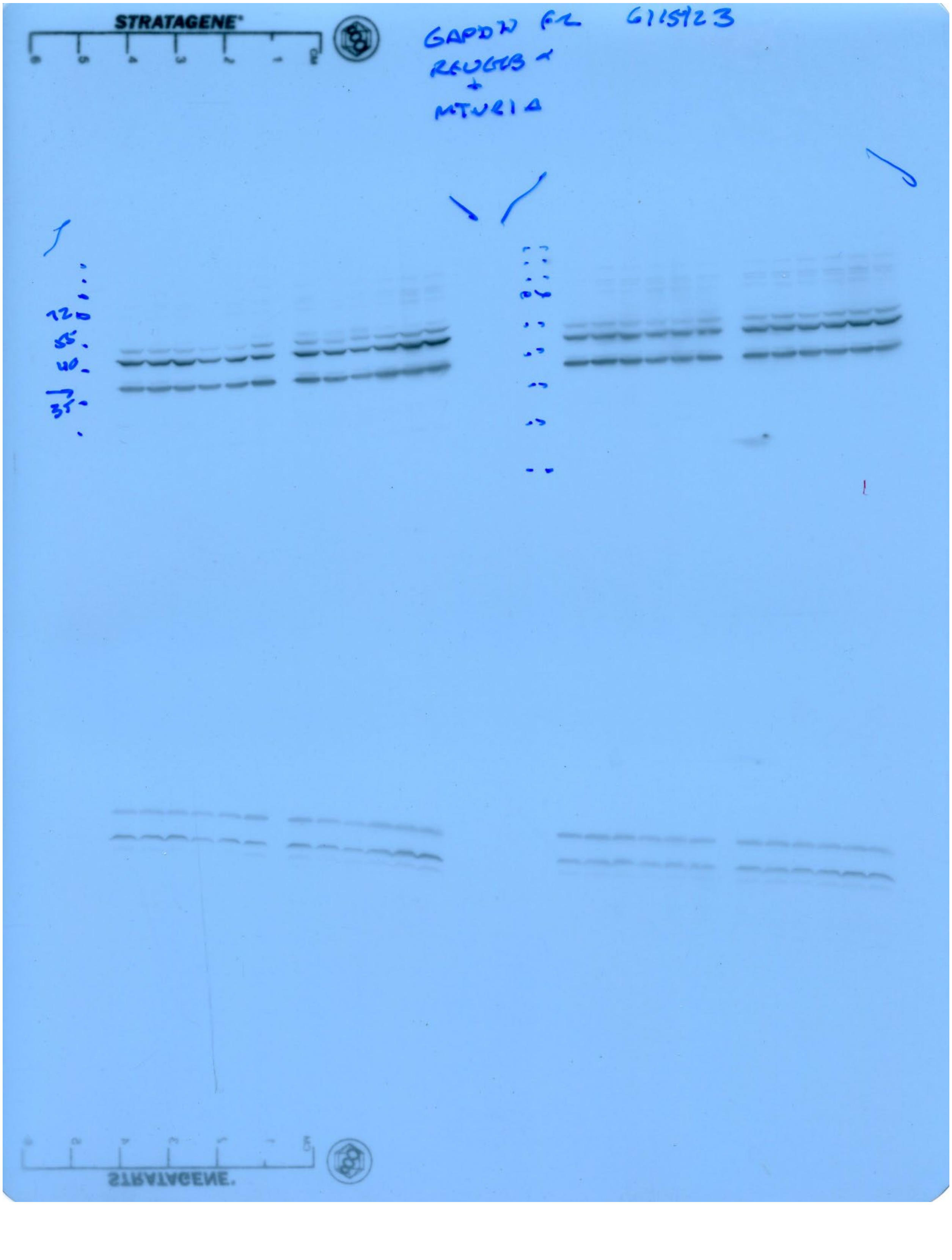
STRATAGENE
GAPDH
72
55
40
35

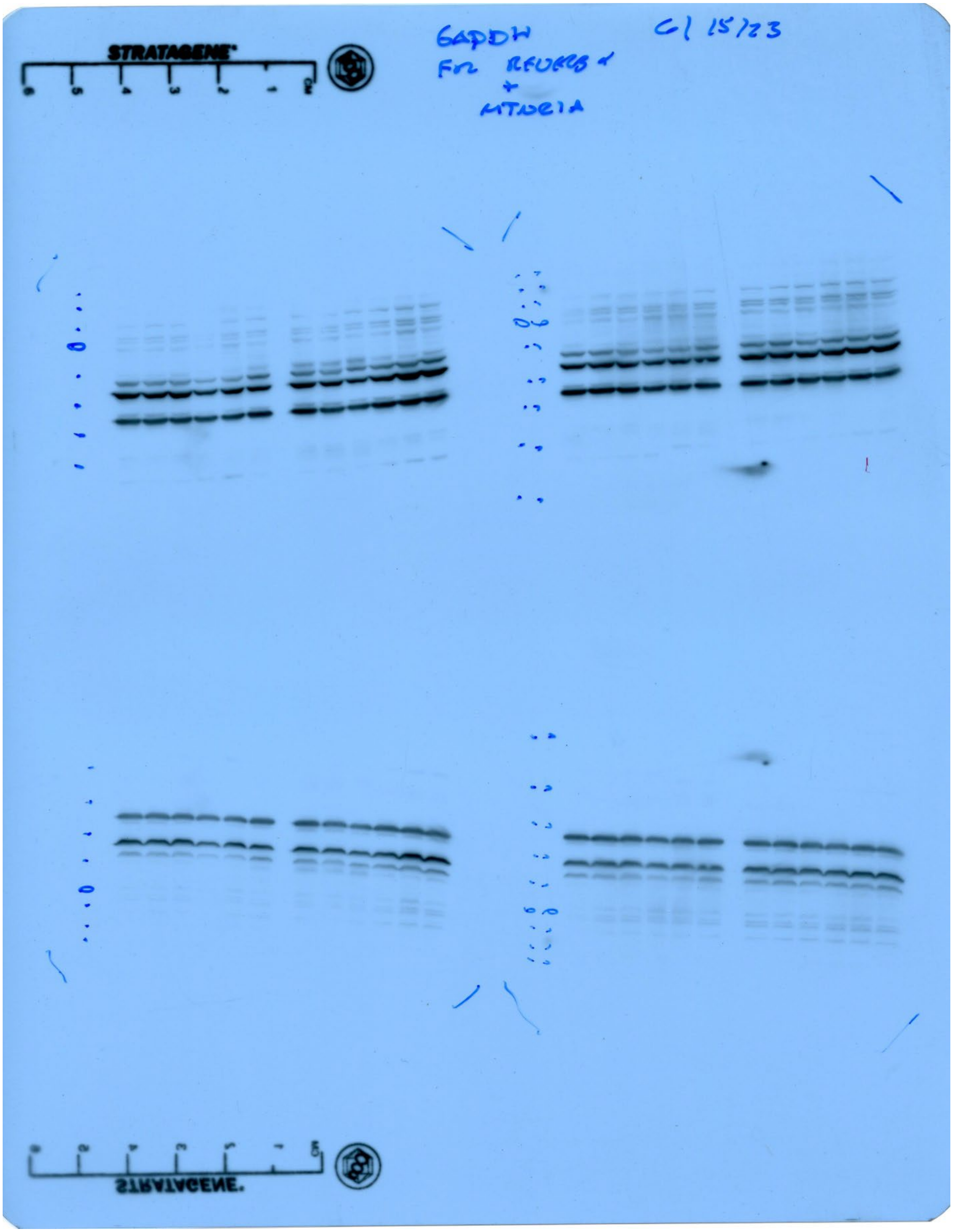
STRATAGENE
GAPDH
For REVERB α
+
MTNR1A
C1 15/23

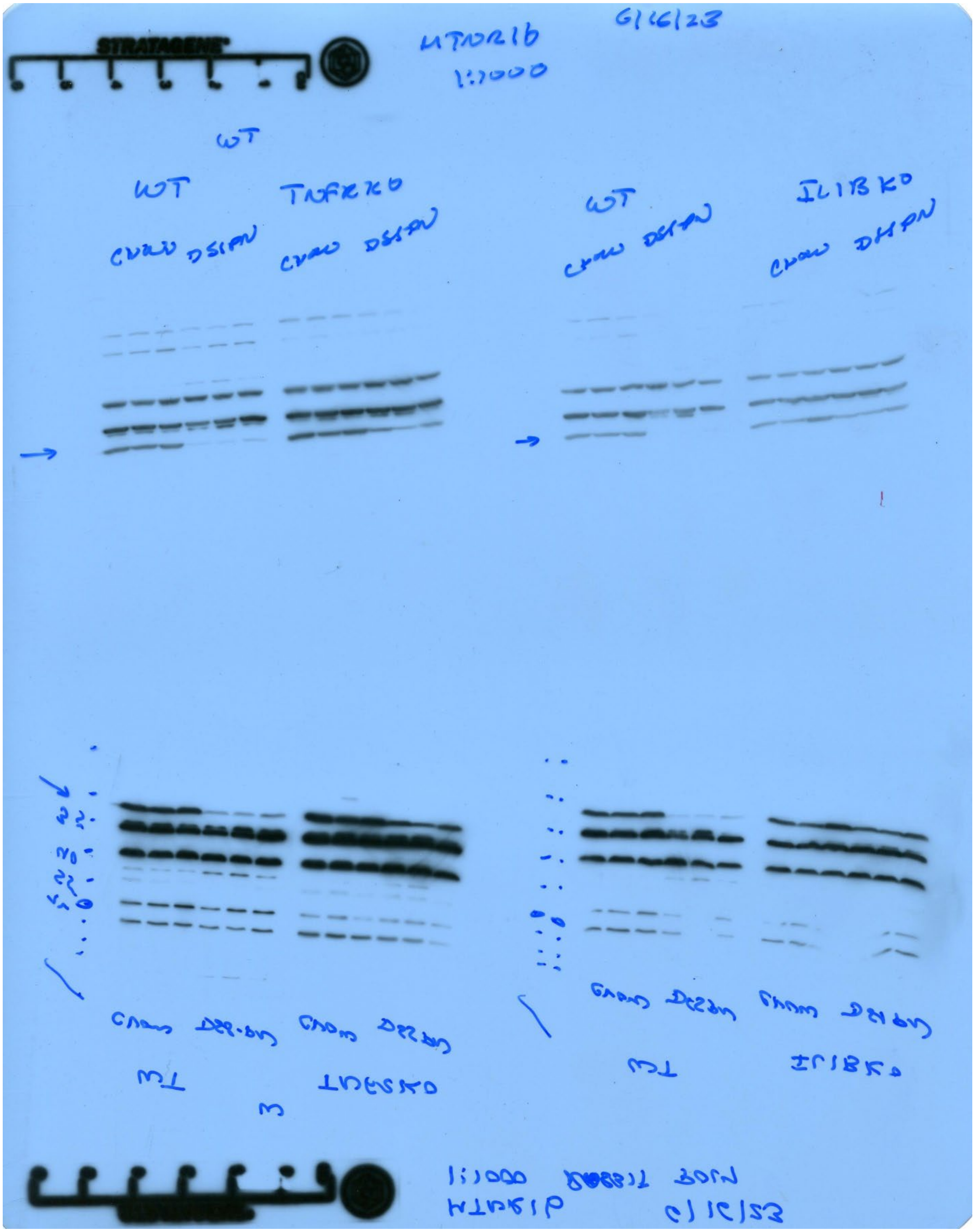

6/16/23
1:1000
WT
WT
TNFRKO
WT
IL1BKO

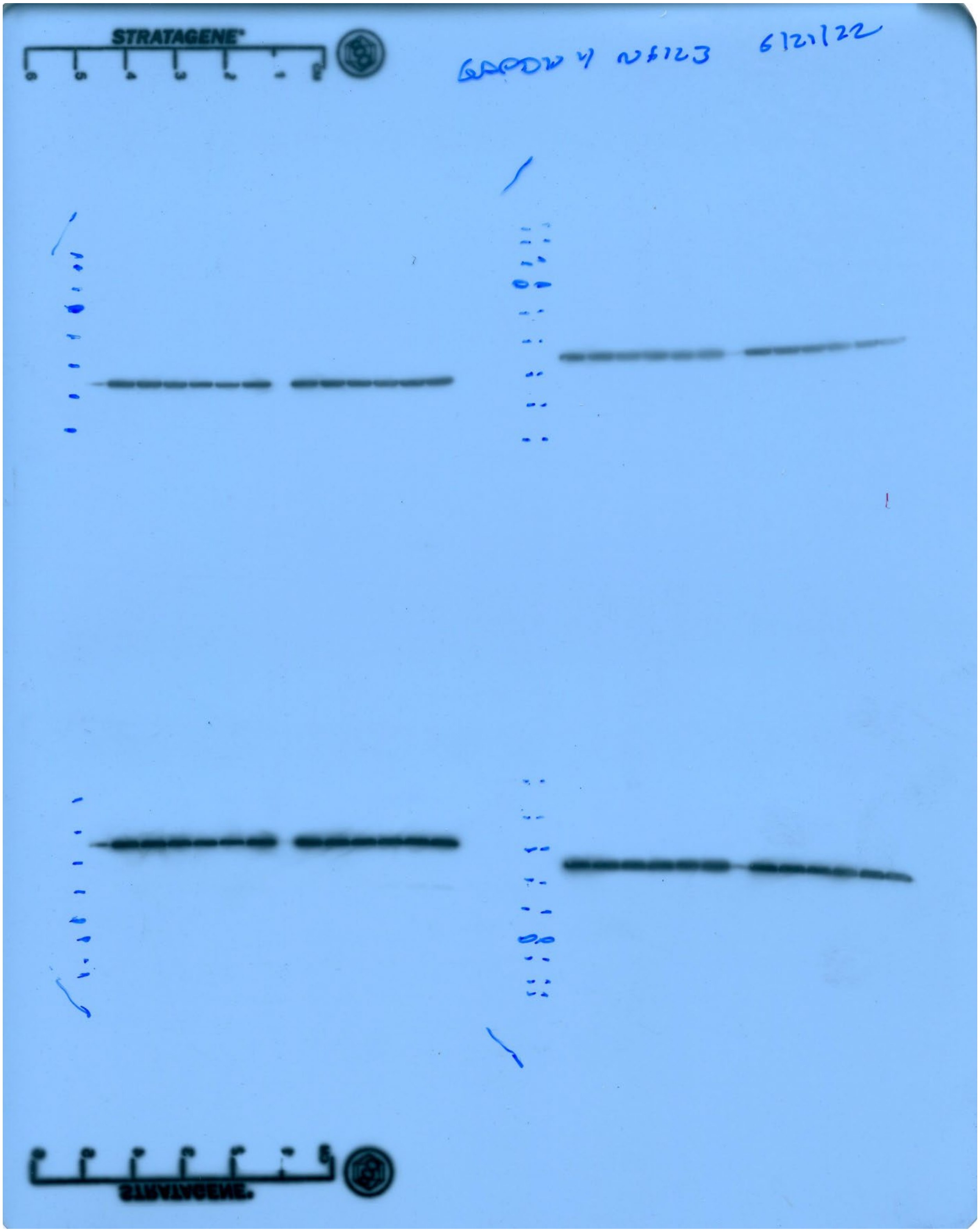
STRATAGENE
GAPDH 4 N#123 6/21/22
STRATAGENE

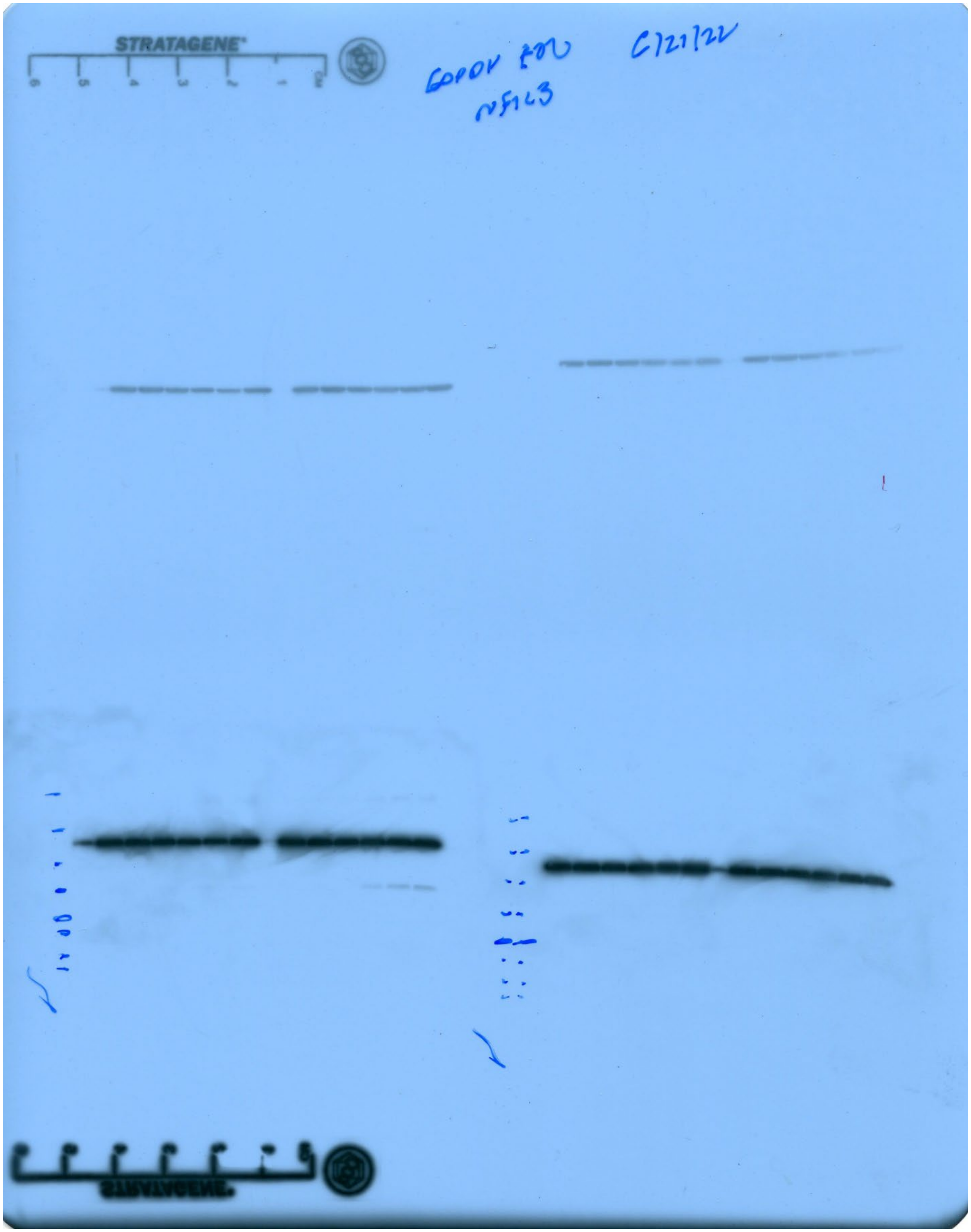
STRATAGENE
GAPDH for
NFIL3
6/21/22

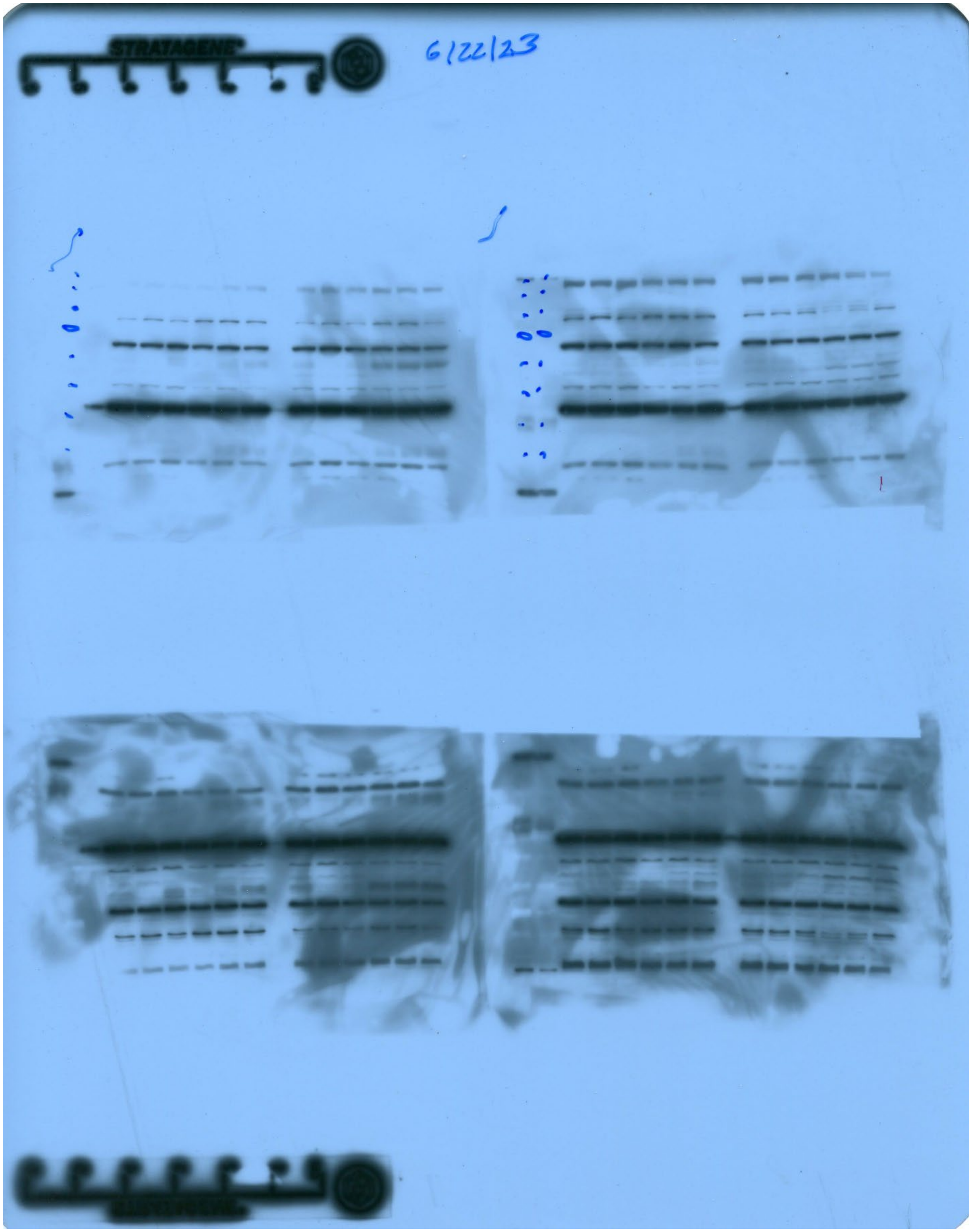
STRATAGENE
6/22/23

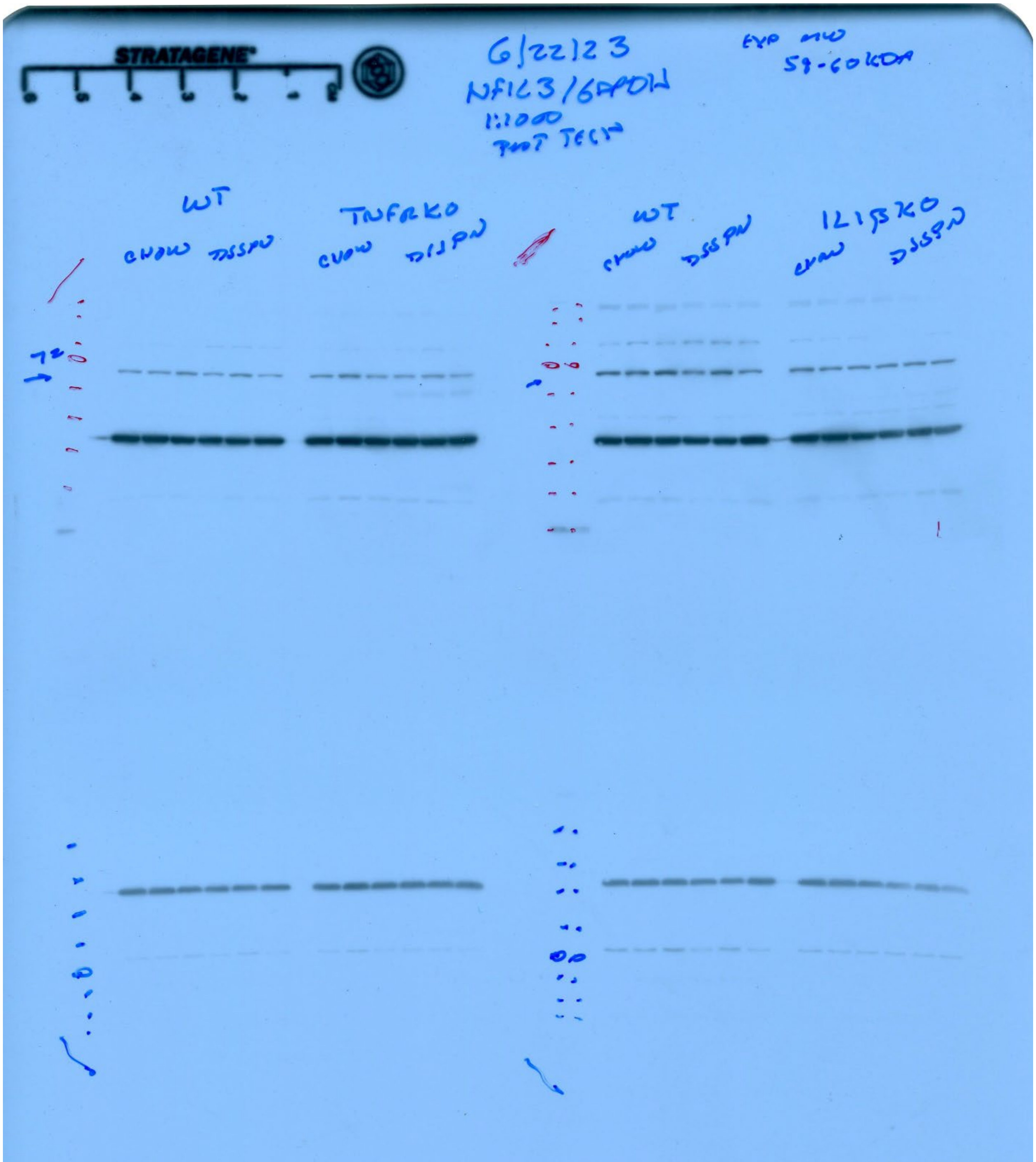
6/22/23
NFIL3/GAPDH
1:1000
EXP MW
WT
TNFRKO
chow

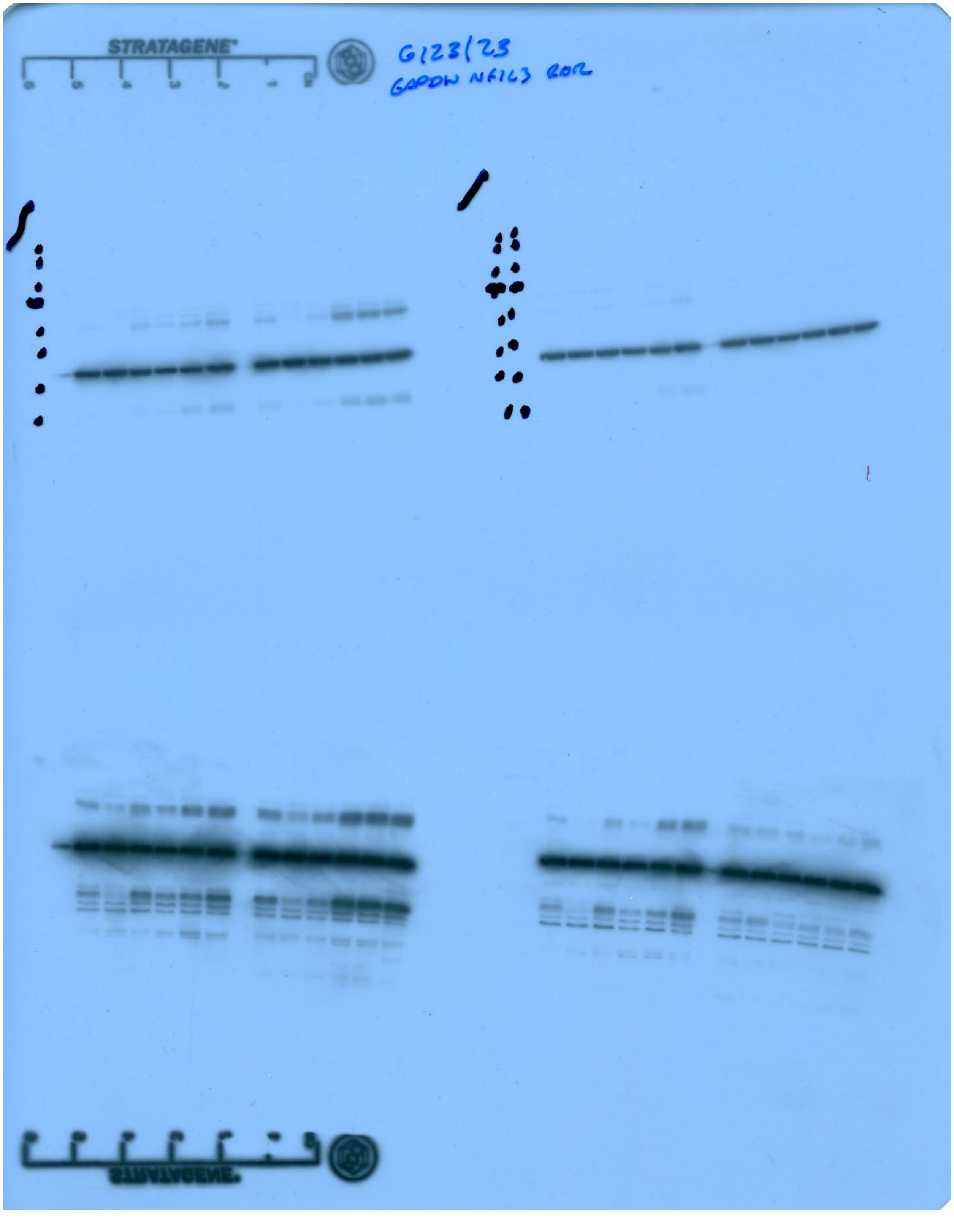
STRATAGENE
6/23/23
GAPDH NFIL3 ROR

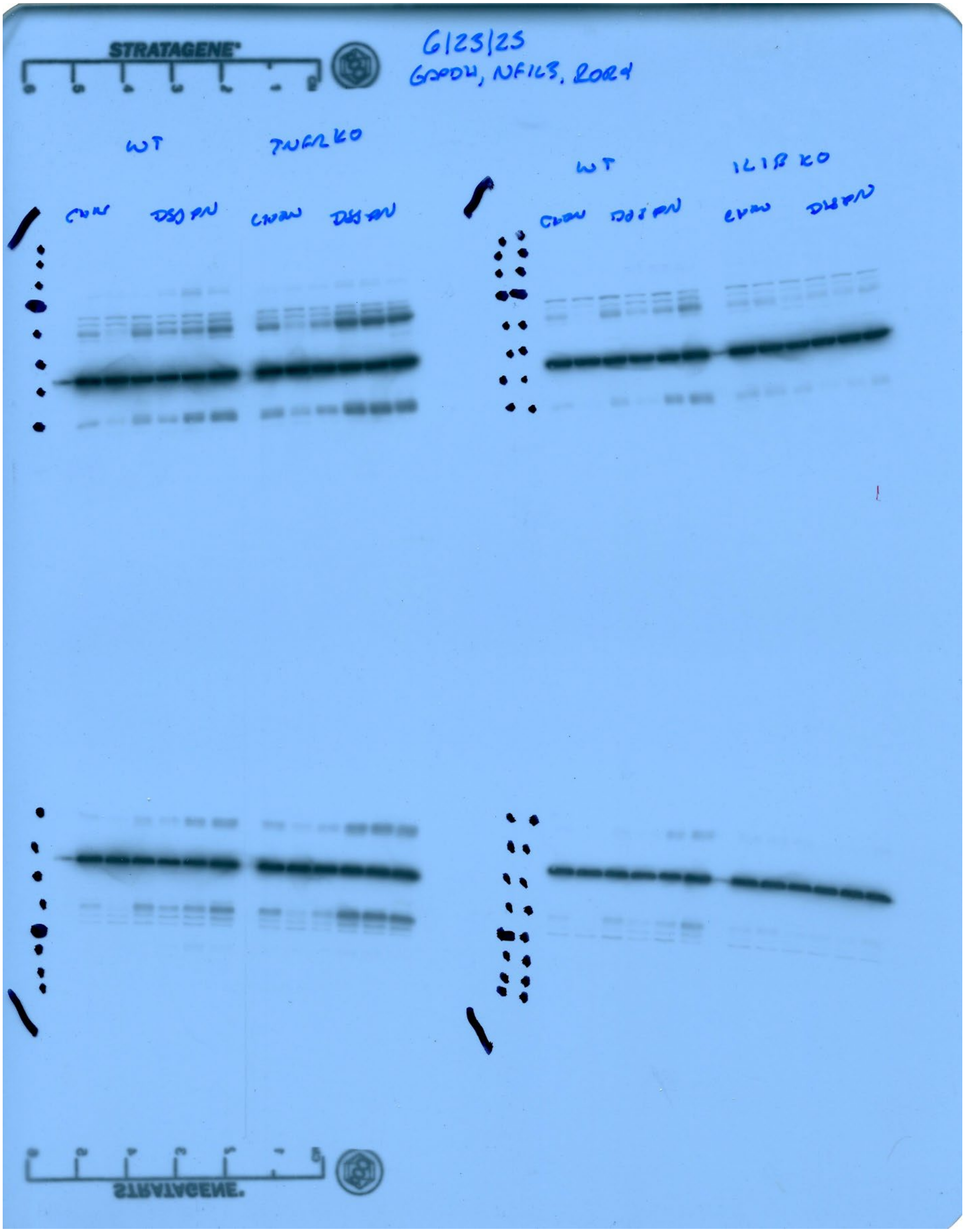
STRATAGENE
6/23/25
GAPDH, NFIL3, RORγ
WT
WT

06/29/2023 Shearn CK7 quantification

method: shoot ten 100x images in a tiling fashion across your CK7 IHC stained slide with all camera settin

		Image ID	total pixels
1	Slide: Shearn 06-28-2023 all	Image: 604 CK7 100x 1.tif	**1920000**
1	Slide: Shearn 06-28-2023 all	Image: 604 CK7 100x 2.tif	**1920000**
1	Slide: Shearn 06-28-2023 all	Image: 604 CK7 100x 3.tif	**1920000**
1	Slide: Shearn 06-28-2023 all	Image: 604 CK7 100x 4.tif	**1920000**
1	Slide: Shearn 06-28-2023 all	Image: 604 CK7 100x 5.tif	**1920000**
1	Slide: Shearn 06-28-2023 all	Image: 604 CK7 100x 6.tif	**1920000**
1	Slide: Shearn 06-28-2023 all	Image: 604 CK7 100x 7.tif	**1920000**
1	Slide: Shearn 06-28-2023 all	Image: 604 CK7 100x 8.tif	**1920000**
1	Slide: Shearn 06-28-2023 all	Image: 604 CK7 100x 9.tif	**1920000**
1	Slide: Shearn 06-28-2023 all	Image: 604 CK7 100x 10.tif	**1920000**
1	Slide: Shearn 06-28-2023 all	Image: 605 CK7 100x 1.tif	**1920000**
1	Slide: Shearn 06-28-2023 all	Image: 605 CK7 100x 2.tif	**1920000**
1	Slide: Shearn 06-28-2023 all	Image: 605 CK7 100x 3.tif	**1920000**
1	Slide: Shearn 06-28-2023 all	Image: 605 CK7 100x 4.tif	**1920000**
1	Slide: Shearn 06-28-2023 all	Image: 605 CK7 100x 5.tif	**1920000**
1	Slide: Shearn 06-28-2023 all	Image: 605 CK7 100x 6.tif	**1920000**
1	Slide: Shearn 06-28-2023 all	Image: 605 CK7 100x 7.tif	**1920000**
1	Slide: Shearn 06-28-2023 all	Image: 605 CK7 100x 8.tif	**1920000**
1	Slide: Shearn 06-28-2023 all	Image: 605 CK7 100x 9.tif	**1920000**
1	Slide: Shearn 06-28-2023 all	Image: 605 CK7 100x 10.tif	**1920000**
1	Slide: Shearn 06-28-2023 all	Image: 606 CK7 100x 1.tif	**1920000**
1	Slide: Shearn 06-28-2023 all	Image: 606 CK7 100x 2.tif	**1920000**
1	Slide: Shearn 06-28-2023 all	Image: 606 CK7 100x 3.tif	**1920000**
1	Slide: Shearn 06-28-2023 all	Image: 606 CK7 100x 4.tif	**1920000**
1	Slide: Shearn 06-28-2023 all	Image: 606 CK7 100x 5.tif	**1920000**
1	Slide: Shearn 06-28-2023 all	Image: 606 CK7 100x 6.tif	**1920000**
1	Slide: Shearn 06-28-2023 all	Image: 606 CK7 100x 7.tif	**1920000**
1	Slide: Shearn 06-28-2023 all	Image: 606 CK7 100x 8.tif	**1920000**
1	Slide: Shearn 06-28-2023 all	Image: 606 CK7 100x 9.tif	**1920000**
1	Slide: Shearn 06-28-2023 all	Image: 606 CK7 100x 10.tif	**1920000**
1	Slide: Shearn 06-28-2023 all	Image: 616 CK7 100x 1.tif	**1920000**
1	Slide: Shearn 06-28-2023 all	Image: 616 CK7 100x 2.tif	**1920000**
1	Slide: Shearn 06-28-2023 all	Image: 616 CK7 100x 3.tif	**1920000**
1	Slide: Shearn 06-28-2023 all	Image: 616 CK7 100x 4.tif	**1920000**
1	Slide: Shearn 06-28-2023 all	Image: 616 CK7 100x 5.tif	**1920000**
1	Slide: Shearn 06-28-2023 all	Image: 616 CK7 100x 6.tif	**1920000**
1	Slide: Shearn 06-28-2023 all	Image: 616 CK7 100x 7.tif	**1920000**
1	Slide: Shearn 06-28-2023 all	Image: 616 CK7 100x 8.tif	**1920000**
1	Slide: Shearn 06-28-2023 all	Image: 616 CK7 100x 9.tif	**1920000**
1	Slide: Shearn 06-28-2023 all	Image: 616 CK7 100x 10.tif	**1920000**
1	Slide: Shearn 06-28-2023 all	Image: 620 CK7 100x 1.tif	**1920000**
1	Slide: Shearn 06-28-2023 all	Image: 620 CK7 100x 2.tif	**1920000**
1	Slide: Shearn 06-28-2023 all	Image: 620 CK7 100x 3.tif	**1920000**

1 Slide: Shearn 06-28-2023 all	Image: 620 CK7 100x 4.tif	**1920000**
1 Slide: Shearn 06-28-2023 all	Image: 620 CK7 100x 5.tif	**1920000**
1 Slide: Shearn 06-28-2023 all	Image: 620 CK7 100x 6.tif	**1920000**
1 Slide: Shearn 06-28-2023 all	Image: 620 CK7 100x 7.tif	**1920000**
1 Slide: Shearn 06-28-2023 all	Image: 620 CK7 100x 8.tif	**1920000**
1 Slide: Shearn 06-28-2023 all	Image: 620 CK7 100x 9.tif	**1920000**
1 Slide: Shearn 06-28-2023 all	Image: 620 CK7 100x 10.tif	**1920000**
1 Slide: Shearn 06-28-2023 all	Image: 737 CK7 100x 1.tif	**1920000**
1 Slide: Shearn 06-28-2023 all	Image: 737 CK7 100x 2.tif	**1920000**
1 Slide: Shearn 06-28-2023 all	Image: 737 CK7 100x 3.tif	**1920000**
1 Slide: Shearn 06-28-2023 all	Image: 737 CK7 100x 4.tif	**1920000**
1 Slide: Shearn 06-28-2023 all	Image: 737 CK7 100x 5.tif	**1920000**
1 Slide: Shearn 06-28-2023 all	Image: 737 CK7 100x 6.tif	**1920000**
1 Slide: Shearn 06-28-2023 all	Image: 737 CK7 100x 7.tif	**1920000**
1 Slide: Shearn 06-28-2023 all	Image: 737 CK7 100x 8.tif	**1920000**
1 Slide: Shearn 06-28-2023 all	Image: 737 CK7 100x 9.tif	**1920000**
1 Slide: Shearn 06-28-2023 all	Image: 737 CK7 100x 10.tif	**1920000**
1 Slide: Shearn 06-28-2023 all	Image: 938 CK7 100x 1.tif	**1920000**
1 Slide: Shearn 06-28-2023 all	Image: 938 CK7 100x 2.tif	**1920000**
1 Slide: Shearn 06-28-2023 all	Image: 938 CK7 100x 3.tif	**1920000**
1 Slide: Shearn 06-28-2023 all	Image: 938 CK7 100x 4.tif	**1920000**
1 Slide: Shearn 06-28-2023 all	Image: 938 CK7 100x 5.tif	**1920000**
1 Slide: Shearn 06-28-2023 all	Image: 938 CK7 100x 6.tif	**1920000**
1 Slide: Shearn 06-28-2023 all	Image: 938 CK7 100x 7.tif	**1920000**
1 Slide: Shearn 06-28-2023 all	Image: 938 CK7 100x 8.tif	**1920000**
1 Slide: Shearn 06-28-2023 all	Image: 938 CK7 100x 9.tif	**1920000**
1 Slide: Shearn 06-28-2023 all	Image: 938 CK7 100x 10.tif	**1920000**
1 Slide: Shearn 06-28-2023 all	Image: 939 CK7 100x 1.tif	**1920000**
1 Slide: Shearn 06-28-2023 all	Image: 939 CK7 100x 2.tif	**1920000**
1 Slide: Shearn 06-28-2023 all	Image: 939 CK7 100x 3.tif	**1920000**
1 Slide: Shearn 06-28-2023 all	Image: 939 CK7 100x 4.tif	**1920000**
1 Slide: Shearn 06-28-2023 all	Image: 939 CK7 100x 5.tif	**1920000**
1 Slide: Shearn 06-28-2023 all	Image: 939 CK7 100x 6.tif	**1920000**
1 Slide: Shearn 06-28-2023 all	Image: 939 CK7 100x 7.tif	**1920000**
1 Slide: Shearn 06-28-2023 all	Image: 939 CK7 100x 8.tif	**1920000**
1 Slide: Shearn 06-28-2023 all	Image: 939 CK7 100x 9.tif	**1920000**
1 Slide: Shearn 06-28-2023 all	Image: 939 CK7 100x 10.tif	**1920000**
1 Slide: Shearn 06-28-2023 all	Image: 940 CK7 100x 1.tif	**1920000**
1 Slide: Shearn 06-28-2023 all	Image: 940 CK7 100x 2.tif	**1920000**
1 Slide: Shearn 06-28-2023 all	Image: 940 CK7 100x 3.tif	**1920000**
1 Slide: Shearn 06-28-2023 all	Image: 940 CK7 100x 4.tif	**1920000**
1 Slide: Shearn 06-28-2023 all	Image: 940 CK7 100x 5.tif	**1920000**
1 Slide: Shearn 06-28-2023 all	Image: 940 CK7 100x 6.tif	**1920000**
1 Slide: Shearn 06-28-2023 all	Image: 940 CK7 100x 7.tif	**1920000**
1 Slide: Shearn 06-28-2023 all	Image: 940 CK7 100x 8.tif	**1920000**
1 Slide: Shearn 06-28-2023 all	Image: 940 CK7 100x 9.tif	**1920000**
1 Slide: Shearn 06-28-2023 all	Image: 940 CK7 100x 10.tif	**1920000**

1	Slide: Shearn 06-28-2023 all	Image: 955 CK7 100x 1.tif	**1920000**
1	Slide: Shearn 06-28-2023 all	Image: 955 CK7 100x 2.tif	**1920000**
1	Slide: Shearn 06-28-2023 all	Image: 955 CK7 100x 3.tif	**1920000**
1	Slide: Shearn 06-28-2023 all	Image: 955 CK7 100x 4.tif	**1920000**
1	Slide: Shearn 06-28-2023 all	Image: 955 CK7 100x 5.tif	**1920000**
1	Slide: Shearn 06-28-2023 all	Image: 955 CK7 100x 6.tif	**1920000**
1	Slide: Shearn 06-28-2023 all	Image: 955 CK7 100x 7.tif	**1920000**
1	Slide: Shearn 06-28-2023 all	Image: 955 CK7 100x 8.tif	**1920000**
1	Slide: Shearn 06-28-2023 all	Image: 955 CK7 100x 9.tif	**1920000**
1	Slide: Shearn 06-28-2023 all	Image: 955 CK7 100x 10.tif	**1920000**
1	Slide: Shearn 06-28-2023 all	Image: 956 CK7 100x 1.tif	**1920000**
1	Slide: Shearn 06-28-2023 all	Image: 956 CK7 100x 2.tif	**1920000**
1	Slide: Shearn 06-28-2023 all	Image: 956 CK7 100x 3.tif	**1920000**
1	Slide: Shearn 06-28-2023 all	Image: 956 CK7 100x 4.tif	**1920000**
1	Slide: Shearn 06-28-2023 all	Image: 956 CK7 100x 5.tif	**1920000**
1	Slide: Shearn 06-28-2023 all	Image: 956 CK7 100x 6.tif	**1920000**
1	Slide: Shearn 06-28-2023 all	Image: 956 CK7 100x 7.tif	**1920000**
1	Slide: Shearn 06-28-2023 all	Image: 956 CK7 100x 8.tif	**1920000**
1	Slide: Shearn 06-28-2023 all	Image: 956 CK7 100x 9.tif	**1920000**
1	Slide: Shearn 06-28-2023 all	Image: 956 CK7 100x 10.tif	**1920000**
1	Slide: Shearn 06-28-2023 all	Image: 957 CK7 100x 1.tif	**1920000**
1	Slide: Shearn 06-28-2023 all	Image: 957 CK7 100x 2.tif	**1920000**
1	Slide: Shearn 06-28-2023 all	Image: 957 CK7 100x 3.tif	**1920000**
1	Slide: Shearn 06-28-2023 all	Image: 957 CK7 100x 4.tif	**1920000**
1	Slide: Shearn 06-28-2023 all	Image: 957 CK7 100x 5.tif	**1920000**
1	Slide: Shearn 06-28-2023 all	Image: 957 CK7 100x 6.tif	**1920000**
1	Slide: Shearn 06-28-2023 all	Image: 957 CK7 100x 7.tif	**1920000**
1	Slide: Shearn 06-28-2023 all	Image: 957 CK7 100x 8.tif	**1920000**
1	Slide: Shearn 06-28-2023 all	Image: 957 CK7 100x 9.tif	**1920000**
1	Slide: Shearn 06-28-2023 all	Image: 957 CK7 100x 10.tif	**1920000**
1	Slide: Shearn 06-28-2023 all	Image: TPN-349 CK7 100x 1.tif	**1920000**
1	Slide: Shearn 06-28-2023 all	Image: TPN-349 CK7 100x 2.tif	**1920000**
1	Slide: Shearn 06-28-2023 all	Image: TPN-349 CK7 100x 3.tif	**1920000**
1	Slide: Shearn 06-28-2023 all	Image: TPN-349 CK7 100x 4.tif	**1920000**
1	Slide: Shearn 06-28-2023 all	Image: TPN-349 CK7 100x 5.tif	**1920000**
1	Slide: Shearn 06-28-2023 all	Image: TPN-349 CK7 100x 6.tif	**1920000**
1	Slide: Shearn 06-28-2023 all	Image: TPN-349 CK7 100x 7.tif	**1920000**
1	Slide: Shearn 06-28-2023 all	Image: TPN-349 CK7 100x 8.tif	**1920000**
1	Slide: Shearn 06-28-2023 all	Image: TPN-349 CK7 100x 9.tif	**1920000**
1	Slide: Shearn 06-28-2023 all	Image: TPN-349 CK7 100x 10.tif	**1920000**
1	Slide: Shearn 06-28-2023 all	Image: TPN-350 CK7 100x 1.tif	**1920000**
1	Slide: Shearn 06-28-2023 all	Image: TPN-350 CK7 100x 2.tif	**1920000**
1	Slide: Shearn 06-28-2023 all	Image: TPN-350 CK7 100x 3.tif	**1920000**
1	Slide: Shearn 06-28-2023 all	Image: TPN-350 CK7 100x 4.tif	**1920000**
1	Slide: Shearn 06-28-2023 all	Image: TPN-350 CK7 100x 5.tif	**1920000**
1	Slide: Shearn 06-28-2023 all	Image: TPN-350 CK7 100x 6.tif	**1920000**
1	Slide: Shearn 06-28-2023 all	Image: TPN-350 CK7 100x 7.tif	**1920000**

1	Slide: Shearn 06-28-2023 all	Image: TPN-350 CK7 100x 8.tif	**1920000**
1	Slide: Shearn 06-28-2023 all	Image: TPN-350 CK7 100x 9.tif	**1920000**
1	Slide: Shearn 06-28-2023 all	Image: TPN-350 CK7 100x 10.tif	**1920000**
1	Slide: Shearn 06-28-2023 all	Image: TPN-351 CK7 100x 1.tif	**1920000**
1	Slide: Shearn 06-28-2023 all	Image: TPN-351 CK7 100x 2.tif	**1920000**
1	Slide: Shearn 06-28-2023 all	Image: TPN-351 CK7 100x 3.tif	**1920000**
1	Slide: Shearn 06-28-2023 all	Image: TPN-351 CK7 100x 4.tif	**1920000**
1	Slide: Shearn 06-28-2023 all	Image: TPN-351 CK7 100x 5.tif	**1920000**
1	Slide: Shearn 06-28-2023 all	Image: TPN-351 CK7 100x 6.tif	**1920000**
1	Slide: Shearn 06-28-2023 all	Image: TPN-351 CK7 100x 7.tif	**1920000**
1	Slide: Shearn 06-28-2023 all	Image: TPN-351 CK7 100x 8.tif	**1920000**
1	Slide: Shearn 06-28-2023 all	Image: TPN-351 CK7 100x 9.tif	**1920000**
1	Slide: Shearn 06-28-2023 all	Image: TPN-351 CK7 100x 10.tif	**1920000**
1	Slide: Shearn 06-28-2023 all	Image: TPN-364 CK7 100x 1.tif	**1920000**
1	Slide: Shearn 06-28-2023 all	Image: TPN-364 CK7 100x 2.tif	**1920000**
1	Slide: Shearn 06-28-2023 all	Image: TPN-364 CK7 100x 3.tif	**1920000**
1	Slide: Shearn 06-28-2023 all	Image: TPN-364 CK7 100x 4.tif	**1920000**
1	Slide: Shearn 06-28-2023 all	Image: TPN-364 CK7 100x 5.tif	**1920000**
1	Slide: Shearn 06-28-2023 all	Image: TPN-364 CK7 100x 6.tif	**1920000**
1	Slide: Shearn 06-28-2023 all	Image: TPN-364 CK7 100x 7.tif	**1920000**
1	Slide: Shearn 06-28-2023 all	Image: TPN-364 CK7 100x 8.tif	**1920000**
1	Slide: Shearn 06-28-2023 all	Image: TPN-364 CK7 100x 9.tif	**1920000**
1	Slide: Shearn 06-28-2023 all	Image: TPN-364 CK7 100x 10.tif	**1920000**
1	Slide: Shearn 06-28-2023 all	Image: TPN-365 CK7 100x 1.tif	**1920000**
1	Slide: Shearn 06-28-2023 all	Image: TPN-365 CK7 100x 2.tif	**1920000**
1	Slide: Shearn 06-28-2023 all	Image: TPN-365 CK7 100x 3.tif	**1920000**
1	Slide: Shearn 06-28-2023 all	Image: TPN-365 CK7 100x 4.tif	**1920000**
1	Slide: Shearn 06-28-2023 all	Image: TPN-365 CK7 100x 5.tif	**1920000**
1	Slide: Shearn 06-28-2023 all	Image: TPN-365 CK7 100x 6.tif	**1920000**
1	Slide: Shearn 06-28-2023 all	Image: TPN-365 CK7 100x 7.tif	**1920000**
1	Slide: Shearn 06-28-2023 all	Image: TPN-365 CK7 100x 8.tif	**1920000**
1	Slide: Shearn 06-28-2023 all	Image: TPN-365 CK7 100x 9.tif	**1920000**
1	Slide: Shearn 06-28-2023 all	Image: TPN-365 CK7 100x 10.tif	**1920000**
2	Image: 604 CK7 100x 1.tif		
2	Image: 604 CK7 100x 2.tif		
2	Image: 604 CK7 100x 3.tif		
2	Image: 604 CK7 100x 4.tif		
2	Image: 604 CK7 100x 5.tif		
2	Image: 604 CK7 100x 6.tif		
2	Image: 604 CK7 100x 7.tif		
2	Image: 604 CK7 100x 8.tif		
2	Image: 604 CK7 100x 9.tif		
2	Image: 604 CK7 100x 10.tif		
2	Image: 605 CK7 100x 1.tif		
2	Image: 605 CK7 100x 2.tif		
2	Image: 605 CK7 100x 3.tif		
2	Image: 605 CK7 100x 4.tif		

2 Image: 605 CK7 100x 5.tif
2 Image: 605 CK7 100x 6.tif
2 Image: 605 CK7 100x 7.tif
2 Image: 605 CK7 100x 8.tif
2 Image: 605 CK7 100x 9.tif
2 Image: 605 CK7 100x 10.tif
2 Image: 606 CK7 100x 1.tif
2 Image: 606 CK7 100x 2.tif
2 Image: 606 CK7 100x 3.tif
2 Image: 606 CK7 100x 4.tif
2 Image: 606 CK7 100x 5.tif
2 Image: 606 CK7 100x 6.tif
2 Image: 606 CK7 100x 7.tif
2 Image: 606 CK7 100x 8.tif
2 Image: 606 CK7 100x 9.tif
2 Image: 606 CK7 100x 10.tif
2 Image: 616 CK7 100x 1.tif
2 Image: 616 CK7 100x 2.tif
2 Image: 616 CK7 100x 3.tif
2 Image: 616 CK7 100x 4.tif
2 Image: 616 CK7 100x 5.tif
2 Image: 616 CK7 100x 6.tif
2 Image: 616 CK7 100x 7.tif
2 Image: 616 CK7 100x 8.tif
2 Image: 616 CK7 100x 9.tif
2 Image: 616 CK7 100x 10.tif
2 Image: 620 CK7 100x 1.tif
2 Image: 620 CK7 100x 2.tif
2 Image: 620 CK7 100x 3.tif
2 Image: 620 CK7 100x 4.tif
2 Image: 620 CK7 100x 5.tif
2 Image: 620 CK7 100x 6.tif
2 Image: 620 CK7 100x 7.tif
2 Image: 620 CK7 100x 8.tif
2 Image: 620 CK7 100x 9.tif
2 Image: 620 CK7 100x 10.tif
2 Image: 737 CK7 100x 1.tif
2 Image: 737 CK7 100x 2.tif
2 Image: 737 CK7 100x 3.tif
2 Image: 737 CK7 100x 4.tif
2 Image: 737 CK7 100x 5.tif
2 Image: 737 CK7 100x 6.tif
2 Image: 737 CK7 100x 7.tif
2 Image: 737 CK7 100x 8.tif
2 Image: 737 CK7 100x 9.tif
2 Image: 737 CK7 100x 10.tif
2 Image: 938 CK7 100x 1.tif

2 Image: 938 CK7 100x 2.tif
2 Image: 938 CK7 100x 3.tif
2 Image: 938 CK7 100x 4.tif
2 Image: 938 CK7 100x 5.tif
2 Image: 938 CK7 100x 6.tif
2 Image: 938 CK7 100x 7.tif
2 Image: 938 CK7 100x 8.tif
2 Image: 938 CK7 100x 9.tif
2 Image: 938 CK7 100x 10.tif
2 Image: 939 CK7 100x 1.tif
2 Image: 939 CK7 100x 2.tif
2 Image: 939 CK7 100x 3.tif
2 Image: 939 CK7 100x 4.tif
2 Image: 939 CK7 100x 5.tif
2 Image: 939 CK7 100x 6.tif
2 Image: 939 CK7 100x 7.tif
2 Image: 939 CK7 100x 8.tif
2 Image: 939 CK7 100x 9.tif
2 Image: 939 CK7 100x 10.tif
2 Image: 940 CK7 100x 1.tif
2 Image: 940 CK7 100x 2.tif
2 Image: 940 CK7 100x 3.tif
2 Image: 940 CK7 100x 4.tif
2 Image: 940 CK7 100x 5.tif
2 Image: 940 CK7 100x 6.tif
2 Image: 940 CK7 100x 7.tif
2 Image: 940 CK7 100x 8.tif
2 Image: 940 CK7 100x 9.tif
2 Image: 940 CK7 100x 10.tif
2 Image: 955 CK7 100x 1.tif
2 Image: 955 CK7 100x 2.tif
2 Image: 955 CK7 100x 3.tif
2 Image: 955 CK7 100x 4.tif
2 Image: 955 CK7 100x 5.tif
2 Image: 955 CK7 100x 6.tif
2 Image: 955 CK7 100x 7.tif
2 Image: 955 CK7 100x 8.tif
2 Image: 955 CK7 100x 9.tif
2 Image: 955 CK7 100x 10.tif
2 Image: 956 CK7 100x 1.tif
2 Image: 956 CK7 100x 2.tif
2 Image: 956 CK7 100x 3.tif
2 Image: 956 CK7 100x 4.tif
2 Image: 956 CK7 100x 5.tif
2 Image: 956 CK7 100x 6.tif
2 Image: 956 CK7 100x 7.tif
2 Image: 956 CK7 100x 8.tif

2 Image: 956 CK7 100x 9.tif
2 Image: 956 CK7 100x 10.tif
2 Image: 957 CK7 100x 1.tif
2 Image: 957 CK7 100x 2.tif
2 Image: 957 CK7 100x 3.tif
2 Image: 957 CK7 100x 4.tif
2 Image: 957 CK7 100x 5.tif
2 Image: 957 CK7 100x 6.tif
2 Image: 957 CK7 100x 7.tif
2 Image: 957 CK7 100x 8.tif
2 Image: 957 CK7 100x 9.tif
2 Image: 957 CK7 100x 10.tif
2 Image: TPN-349 CK7 100x 1.tif
2 Image: TPN-349 CK7 100x 2.tif
2 Image: TPN-349 CK7 100x 3.tif
2 Image: TPN-349 CK7 100x 4.tif
2 Image: TPN-349 CK7 100x 5.tif
2 Image: TPN-349 CK7 100x 6.tif
2 Image: TPN-349 CK7 100x 7.tif
2 Image: TPN-349 CK7 100x 8.tif
2 Image: TPN-349 CK7 100x 9.tif
2 Image: TPN-349 CK7 100x 10.tif
2 Image: TPN-350 CK7 100x 1.tif
2 Image: TPN-350 CK7 100x 2.tif
2 Image: TPN-350 CK7 100x 3.tif
2 Image: TPN-350 CK7 100x 4.tif
2 Image: TPN-350 CK7 100x 5.tif
2 Image: TPN-350 CK7 100x 6.tif
2 Image: TPN-350 CK7 100x 7.tif
2 Image: TPN-350 CK7 100x 8.tif
2 Image: TPN-350 CK7 100x 9.tif
2 Image: TPN-350 CK7 100x 10.tif
2 Image: TPN-351 CK7 100x 1.tif
2 Image: TPN-351 CK7 100x 2.tif
2 Image: TPN-351 CK7 100x 3.tif
2 Image: TPN-351 CK7 100x 4.tif
2 Image: TPN-351 CK7 100x 5.tif
2 Image: TPN-351 CK7 100x 6.tif
2 Image: TPN-351 CK7 100x 7.tif
2 Image: TPN-351 CK7 100x 8.tif
2 Image: TPN-351 CK7 100x 9.tif
2 Image: TPN-351 CK7 100x 10.tif
2 Image: TPN-364 CK7 100x 1.tif
2 Image: TPN-364 CK7 100x 2.tif
2 Image: TPN-364 CK7 100x 3.tif
2 Image: TPN-364 CK7 100x 4.tif
2 Image: TPN-364 CK7 100x 5.tif

2 Image: TPN-364 CK7 100x 6.tif
2 Image: TPN-364 CK7 100x 7.tif
2 Image: TPN-364 CK7 100x 8.tif
2 Image: TPN-364 CK7 100x 9.tif
2 Image: TPN-364 CK7 100x 10.tif
2 Image: TPN-365 CK7 100x 1.tif
2 Image: TPN-365 CK7 100x 2.tif
2 Image: TPN-365 CK7 100x 3.tif
2 Image: TPN-365 CK7 100x 4.tif
2 Image: TPN-365 CK7 100x 5.tif
2 Image: TPN-365 CK7 100x 6.tif
2 Image: TPN-365 CK7 100x 7.tif
2 Image: TPN-365 CK7 100x 8.tif
2 Image: TPN-365 CK7 100x 9.tif
2 Image: TPN-365 CK7 100x 10.tif
3 Mask: dapi-0-135
3 Mask: dapi-0-135
3 Mask: dapi-0-135
3 Mask: dapi-0-135
3 Mask: dapi-0-135
3 Mask: dapi-0-135
3 Mask: dapi-0-135
3 Mask: dapi-0-135
3 Mask: dapi-0-135
3 Mask: dapi-0-135
3 Mask: dapi-0-135
3 Mask: dapi-0-135
3 Mask: dapi-0-135
3 Mask: dapi-0-135
3 Mask: dapi-0-135
3 Mask: dapi-0-135
3 Mask: dapi-0-135
3 Mask: dapi-0-135
3 Mask: dapi-0-135
3 Mask: dapi-0-135
3 Mask: dapi-0-135
3 Mask: dapi-0-135
3 Mask: dapi-0-135
3 Mask: dapi-0-135
3 Mask: dapi-0-135
3 Mask: dapi-0-135
3 Mask: dapi-0-135
3 Mask: dapi-0-135
3 Mask: dapi-0-135
3 Mask: dapi-0-135
3 Mask: dapi-0-135
3 Mask: dapi-0-135

3	Mask: dapi-0-135
3	Mask: dapi-0-135
3	Mask: dapi-0-135
3	Mask: dapi-0-135
3	Mask: dapi-0-135
3	Mask: dapi-0-135
3	Mask: dapi-0-135
3	Mask: dapi-0-135
3	Mask: dapi-0-135
3	Mask: dapi-0-135
3	Mask: dapi-0-135
3	Mask: dapi-0-135
3	Mask: dapi-0-135
3	Mask: dapi-0-135
3	Mask: dapi-0-135
3	Mask: dapi-0-135
3	Mask: dapi-0-135
3	Mask: dapi-0-135
3	Mask: dapi-0-135
3	Mask: dapi-0-135
3	Mask: dapi-0-135
3	Mask: dapi-0-135
3	Mask: dapi-0-135
3	Mask: dapi-0-135
3	Mask: dapi-0-135
3	Mask: dapi-0-135
3	Mask: dapi-0-135
3	Mask: dapi-0-135
3	Mask: dapi-0-135
3	Mask: dapi-0-135
3	Mask: dapi-0-135
3	Mask: dapi-0-135
3	Mask: dapi-0-135
3	Mask: dapi-0-135
3	Mask: dapi-0-135
3	Mask: dapi-0-135
3	Mask: dapi-0-135
3	Mask: dapi-0-135
3	Mask: dapi-0-135
3	Mask: dapi-0-135
3	Mask: dapi-0-135
3	Mask: dapi-0-135
3	Mask: dapi-0-135
3	Mask: dapi-0-135
3	Mask: dapi-0-135
3	Mask: dapi-0-135
3	Mask: dapi-0-135

3 Mask: dapi-0-135
3 Mask: dapi-0-135
3 Mask: dapi-0-135
3 Mask: dapi-0-135
3 Mask: dapi-0-135
3 Mask: dapi-0-135
3 Mask: dapi-0-135
3 Mask: dapi-0-135
3 Mask: dapi-0-135
3 Mask: dapi-0-135
3 Mask: dapi-0-135
3 Mask: dapi-0-135
3 Mask: dapi-0-135
3 Mask: dapi-0-135
3 Mask: dapi-0-135
3 Mask: dapi-0-135
3 Mask: dapi-0-135
3 Mask: dapi-0-135
3 Mask: dapi-0-135
3 Mask: dapi-0-135
3 Mask: dapi-0-135
3 Mask: dapi-0-135
3 Mask: dapi-0-135
3 Mask: dapi-0-135
3 Mask: dapi-0-135
3 Mask: dapi-0-135
3 Mask: dapi-0-135
3 Mask: dapi-0-135
3 Mask: dapi-0-135
3 Mask: dapi-0-135
3 Mask: dapi-0-135
3 Mask: dapi-0-135
3 Mask: dapi-0-135
3 Mask: dapi-0-135
3 Mask: dapi-0-135
3 Mask: dapi-0-135
3 Mask: dapi-0-135
3 Mask: dapi-0-135
3 Mask: dapi-0-135
3 Mask: dapi-0-135
3 Mask: dapi-0-135
3 Mask: dapi-0-135
3 Mask: dapi-0-135
3 Mask: dapi-0-135
3 Mask: dapi-0-135
3 Mask: dapi-0-135
3 Mask: dapi-0-135

3	Mask: dapi-0-135	
3	Mask: dapi-0-135	
3	Mask: dapi-0-135	
3	Mask: dapi-0-135	
3	Mask: dapi-0-135	
3	Mask: dapi-0-135	
3	Mask: dapi-0-135	
3	Mask: dapi-0-135	
3	Mask: dapi-0-135	
3	Mask: dapi-0-135	
3	Mask: dapi-0-135	
3	Mask: dapi-0-135	
3	Mask: dapi-0-135	
3	Mask: dapi-0-135	
3	Mask: dapi-0-135	
3	Mask: dapi-0-135	
3	Mask: dapi-0-135	
3	Mask: dapi-0-135	
3	Mask: dapi-0-135	
3	Mask: dapi-0-135	
3	Mask: dapi-0-135	
3	Mask: dapi-0-135	
3	Mask: dapi-0-135	
3	Mask: dapi-0-135	
3	Mask: dapi-0-135	
3	Mask: dapi-0-135	
3	Mask: dapi-0-135	
3	Mask: dapi-0-135	
3	Mask: dapi-0-135	
3	Mask: dapi-0-135	
3	Mask: dapi-0-135	
3	Mask: dapi-0-135	
3	Mask: dapi-0-135	
3	Mask: dapi-0-135	
3	Mask: dapi-0-135	
3	Mask: dapi-0-135	
3	Mask: dapi-0-135	
3	Mask: dapi-0-135	
3	Mask: dapi-0-135	
3	Mask: dapi-0-135	
3	Mask: dapi-0-135	
3	Mask: dapi-0-135	
3	Mask: dapi-0-135	
3	Mask: dapi-0-135	
4		6/29/2023 7:11
4		6/29/2023 7:11
4		6/29/2023 7:10

4	6/29/2023 7:10
4	6/29/2023 7:10
4	6/29/2023 7:10
4	6/29/2023 7:09
4	6/29/2023 7:09
4	6/29/2023 7:09
4	6/29/2023 7:09
4	6/29/2023 7:00
4	6/29/2023 7:00
4	6/29/2023 7:00
4	6/29/2023 6:59
4	6/29/2023 6:59
4	6/29/2023 6:59
4	6/29/2023 6:59
4	6/29/2023 6:58
4	6/29/2023 6:58
4	6/29/2023 6:58
4	6/29/2023 7:05
4	6/29/2023 7:05
4	6/29/2023 7:05
4	6/29/2023 7:05
4	6/29/2023 7:04
4	6/29/2023 7:04
4	6/29/2023 7:04
4	6/29/2023 7:04
4	6/29/2023 7:03
4	6/29/2023 7:03
4	6/29/2023 6:55
4	6/29/2023 6:54
4	6/29/2023 6:54
4	6/29/2023 6:54
4	6/29/2023 6:53
4	6/29/2023 6:53
4	6/29/2023 6:53
4	6/29/2023 6:53
4	6/29/2023 6:53
4	6/29/2023 6:52
4	6/29/2023 6:50
4	6/29/2023 6:49
4	6/29/2023 6:49
4	6/29/2023 6:49
4	6/29/2023 6:49
4	6/29/2023 6:49
4	6/29/2023 6:48
4	6/29/2023 6:48
4	6/29/2023 6:48
4	6/29/2023 6:48

4	6/29/2023 6:36
4	6/29/2023 6:35
4	6/29/2023 6:35
4	6/29/2023 6:35
4	6/29/2023 6:35
4	6/29/2023 6:35
4	6/29/2023 6:34
4	6/29/2023 6:34
4	6/29/2023 6:34
4	6/29/2023 6:34
4	6/29/2023 7:17
4	6/29/2023 7:16
4	6/29/2023 7:16
4	6/29/2023 7:16
4	6/29/2023 7:16
4	6/29/2023 7:15
4	6/29/2023 7:15
4	6/29/2023 7:15
4	6/29/2023 7:15
4	6/29/2023 7:14
4	6/29/2023 7:28
4	6/29/2023 7:28
4	6/29/2023 7:27
4	6/29/2023 7:27
4	6/29/2023 7:27
4	6/29/2023 7:26
4	6/29/2023 7:26
4	6/29/2023 7:26
4	6/29/2023 7:26
4	6/29/2023 7:25
4	6/29/2023 7:23
4	6/29/2023 7:22
4	6/29/2023 7:22
4	6/29/2023 7:22
4	6/29/2023 7:21
4	6/29/2023 7:21
4	6/29/2023 7:21
4	6/29/2023 7:20
4	6/29/2023 7:20
4	6/29/2023 7:20
4	6/29/2023 7:38
4	6/29/2023 7:38
4	6/29/2023 7:38
4	6/29/2023 7:38
4	6/29/2023 7:37
4	6/29/2023 7:37
4	6/29/2023 7:37

4	6/29/2023 7:37
4	6/29/2023 7:36
4	6/29/2023 7:36
4	6/29/2023 7:44
4	6/29/2023 7:43
4	6/29/2023 7:43
4	6/29/2023 7:43
4	6/29/2023 7:42
4	6/29/2023 7:42
4	6/29/2023 7:42
4	6/29/2023 7:42
4	6/29/2023 7:41
4	6/29/2023 7:41
4	6/29/2023 7:33
4	6/29/2023 7:32
4	6/29/2023 7:32
4	6/29/2023 7:32
4	6/29/2023 7:32
4	6/29/2023 7:31
4	6/29/2023 7:31
4	6/29/2023 7:31
4	6/29/2023 7:31
4	6/29/2023 7:31
4	6/29/2023 6:31
4	6/29/2023 6:31
4	6/29/2023 6:31
4	6/29/2023 6:31
4	6/29/2023 6:31
4	6/29/2023 6:30
4	6/29/2023 6:30
4	6/29/2023 6:30
4	6/29/2023 6:30
4	6/29/2023 6:30
4	6/29/2023 6:27
4	6/29/2023 6:27
4	6/29/2023 6:27
4	6/29/2023 6:27
4	6/29/2023 6:26
4	6/29/2023 6:26
4	6/29/2023 6:26
4	6/29/2023 6:26
4	6/29/2023 6:26
4	6/29/2023 6:26
4	6/29/2023 6:22
4	6/29/2023 6:22
4	6/29/2023 6:22
4	6/29/2023 6:22

4	6/29/2023 6:22
4	6/29/2023 6:21
4	6/29/2023 6:21
4	6/29/2023 6:21
4	6/29/2023 6:21
4	6/29/2023 6:21
4	6/29/2023 6:45
4	6/29/2023 6:45
4	6/29/2023 6:44
4	6/29/2023 6:44
4	6/29/2023 6:44
4	6/29/2023 6:44
4	6/29/2023 6:43
4	6/29/2023 6:43
4	6/29/2023 6:43
4	6/29/2023 6:43
4	6/29/2023 6:40
4	6/29/2023 6:40
4	6/29/2023 6:40
4	6/29/2023 6:39
4	6/29/2023 6:39
4	6/29/2023 6:39
4	6/29/2023 6:39
4	6/29/2023 6:38
4	6/29/2023 6:38
4	6/29/2023 6:38
5	
5	
5	
5	
5	
5	
5	
5	
5	
5	
5	
5	
5	
5	
5	
5	
5	
5	
5	
5	
5	

5
5
5
5
5
5
5
5
5
5
5
5
5
5
5
5
5
5
5
5
5
5
5
5
5
5
5
5
5
5
5
5
5
5
5
5
5
5
5
5
5
5
5
5
5
5
5

5
5
5
5
5
5
5
5
5
5
5
5
5
5
5
5
5
5
5
5
5
5
5
5
5
5
5
5
5
5
5
5
5
5
5
5
5
5
5
5
5
5
5
5
5
5
5

5
5
5
5
5
5
5
5
5
5
5
5
5
5
5
5
5
5
5
5
5
5
5
5
5
5
5
5
5
5
5
5
5
5
5
5
5
5
5
5
5
5
5
5
5
5
5

5	
5	
5	
5	
5	
5	
5	
5	
6	Entire Masks
6	Entire Masks
6	Entire Masks
6	Entire Masks
6	Entire Masks
6	Entire Masks
6	Entire Masks
6	Entire Masks
6	Entire Masks
6	Entire Masks
6	Entire Masks
6	Entire Masks
6	Entire Masks
6	Entire Masks
6	Entire Masks
6	Entire Masks
6	Entire Masks
6	Entire Masks
6	Entire Masks
6	Entire Masks
6	Entire Masks
6	Entire Masks
6	Entire Masks
6	Entire Masks
6	Entire Masks
6	Entire Masks
6	Entire Masks
6	Entire Masks
6	Entire Masks
6	Entire Masks
6	Entire Masks
6	Entire Masks
6	Entire Masks
6	Entire Masks
6	Entire Masks
6	Entire Masks
6	Entire Masks
6	Entire Masks
6	Entire Masks

6 Entire Masks
6 Entire Masks
6 Entire Masks
6 Entire Masks
6 Entire Masks
6 Entire Masks
6 Entire Masks
6 Entire Masks
6 Entire Masks
6 Entire Masks
6 Entire Masks
6 Entire Masks
6 Entire Masks
6 Entire Masks
6 Entire Masks
6 Entire Masks
6 Entire Masks
6 Entire Masks
6 Entire Masks
6 Entire Masks
6 Entire Masks
6 Entire Masks
6 Entire Masks
6 Entire Masks
6 Entire Masks
6 Entire Masks
6 Entire Masks
6 Entire Masks
6 Entire Masks
6 Entire Masks
6 Entire Masks
6 Entire Masks
6 Entire Masks
6 Entire Masks
6 Entire Masks
6 Entire Masks
6 Entire Masks
6 Entire Masks
6 Entire Masks
6 Entire Masks
6 Entire Masks
6 Entire Masks
6 Entire Masks
6 Entire Masks
6 Entire Masks
6 Entire Masks
6 Entire Masks

6 Entire Masks
6 Entire Masks
6 Entire Masks
6 Entire Masks
6 Entire Masks
6 Entire Masks
6 Entire Masks
6 Entire Masks
6 Entire Masks
6 Entire Masks
6 Entire Masks
6 Entire Masks
6 Entire Masks
6 Entire Masks
6 Entire Masks
6 Entire Masks
6 Entire Masks
6 Entire Masks
6 Entire Masks
6 Entire Masks
6 Entire Masks
6 Entire Masks
6 Entire Masks
6 Entire Masks
6 Entire Masks
6 Entire Masks
6 Entire Masks
6 Entire Masks
6 Entire Masks
6 Entire Masks
6 Entire Masks
6 Entire Masks
6 Entire Masks
6 Entire Masks
6 Entire Masks
6 Entire Masks
6 Entire Masks
6 Entire Masks
6 Entire Masks
6 Entire Masks
6 Entire Masks
6 Entire Masks
6 Entire Masks
6 Entire Masks
6 Entire Masks
6 Entire Masks
6 Entire Masks

6	Entire Masks	
6	Entire Masks	
6	Entire Masks	
6	Entire Masks	
6	Entire Masks	
6	Entire Masks	
6	Entire Masks	
6	Entire Masks	
6	Entire Masks	
6	Entire Masks	
6	Entire Masks	
6	Entire Masks	
6	Entire Masks	
6	Entire Masks	
6	Entire Masks	
6	Entire Masks	
6	Entire Masks	
6	Entire Masks	
6	Entire Masks	
6	Entire Masks	
6	Entire Masks	
6	Entire Masks	
6	Entire Masks	
6	Entire Masks	
6	Entire Masks	
6	Entire Masks	
6	Entire Masks	
6	Entire Masks	
6	Entire Masks	
6	Entire Masks	
6	Entire Masks	
6	Entire Masks	
6	Entire Masks	
6	Entire Masks	
6	Entire Masks	
6	Entire Masks	
6	Entire Masks	
7	Mask	Area (pixels)
7	Mask	Area (pixels)
7	Mask	Area (pixels)
7	Mask	Area (pixels)
7	Mask	Area (pixels)
7	Mask	Area (pixels)
7	Mask	Area (pixels)
7	Mask	Area (pixels)
7	Mask	Area (pixels)
7	Mask	Area (pixels)

7	Mask	Area (pixels)
7	Mask	Area (pixels)
7	Mask	Area (pixels)
7	Mask	Area (pixels)
7	Mask	Area (pixels)
7	Mask	Area (pixels)
7	Mask	Area (pixels)
7	Mask	Area (pixels)
7	Mask	Area (pixels)
7	Mask	Area (pixels)
7	Mask	Area (pixels)
7	Mask	Area (pixels)
7	Mask	Area (pixels)
7	Mask	Area (pixels)
7	Mask	Area (pixels)
7	Mask	Area (pixels)
7	Mask	Area (pixels)
7	Mask	Area (pixels)
7	Mask	Area (pixels)
7	Mask	Area (pixels)
7	Mask	Area (pixels)
7	Mask	Area (pixels)
7	Mask	Area (pixels)
7	Mask	Area (pixels)
7	Mask	Area (pixels)
7	Mask	Area (pixels)
7	Mask	Area (pixels)
7	Mask	Area (pixels)
7	Mask	Area (pixels)
7	Mask	Area (pixels)
7	Mask	Area (pixels)
7	Mask	Area (pixels)
7	Mask	Area (pixels)
7	Mask	Area (pixels)
7	Mask	Area (pixels)
7	Mask	Area (pixels)
7	Mask	Area (pixels)
7	Mask	Area (pixels)
7	Mask	Area (pixels)
7	Mask	Area (pixels)
7	Mask	Area (pixels)
7	Mask	Area (pixels)
7	Mask	Area (pixels)
7	Mask	Area (pixels)
7	Mask	Area (pixels)
7	Mask	Area (pixels)
7	Mask	Area (pixels)

7	Mask	Area (pixels)
7	Mask	Area (pixels)
7	Mask	Area (pixels)
7	Mask	Area (pixels)
7	Mask	Area (pixels)
7	Mask	Area (pixels)
7	Mask	Area (pixels)
7	Mask	Area (pixels)
7	Mask	Area (pixels)
7	Mask	Area (pixels)
7	Mask	Area (pixels)
7	Mask	Area (pixels)
7	Mask	Area (pixels)
7	Mask	Area (pixels)
7	Mask	Area (pixels)
7	Mask	Area (pixels)
7	Mask	Area (pixels)
7	Mask	Area (pixels)
7	Mask	Area (pixels)
7	Mask	Area (pixels)
7	Mask	Area (pixels)
7	Mask	Area (pixels)
7	Mask	Area (pixels)
7	Mask	Area (pixels)
7	Mask	Area (pixels)
7	Mask	Area (pixels)
7	Mask	Area (pixels)
7	Mask	Area (pixels)
7	Mask	Area (pixels)
7	Mask	Area (pixels)
7	Mask	Area (pixels)
7	Mask	Area (pixels)
7	Mask	Area (pixels)
7	Mask	Area (pixels)
7	Mask	Area (pixels)
7	Mask	Area (pixels)
7	Mask	Area (pixels)
7	Mask	Area (pixels)
7	Mask	Area (pixels)
7	Mask	Area (pixels)
7	Mask	Area (pixels)
7	Mask	Area (pixels)
7	Mask	Area (pixels)
7	Mask	Area (pixels)
7	Mask	Area (pixels)
7	Mask	Area (pixels)
7	Mask	Area (pixels)

7	Mask	Area (pixels)
7	Mask	Area (pixels)
7	Mask	Area (pixels)
7	Mask	Area (pixels)
7	Mask	Area (pixels)
7	Mask	Area (pixels)
7	Mask	Area (pixels)
7	Mask	Area (pixels)
7	Mask	Area (pixels)
7	Mask	Area (pixels)
7	Mask	Area (pixels)
7	Mask	Area (pixels)
7	Mask	Area (pixels)
7	Mask	Area (pixels)
7	Mask	Area (pixels)
7	Mask	Area (pixels)
7	Mask	Area (pixels)
7	Mask	Area (pixels)
7	Mask	Area (pixels)
7	Mask	Area (pixels)
7	Mask	Area (pixels)
7	Mask	Area (pixels)
7	Mask	Area (pixels)
7	Mask	Area (pixels)
7	Mask	Area (pixels)
7	Mask	Area (pixels)
7	Mask	Area (pixels)
7	Mask	Area (pixels)
7	Mask	Area (pixels)
7	Mask	Area (pixels)
7	Mask	Area (pixels)
7	Mask	Area (pixels)
7	Mask	Area (pixels)
7	Mask	Area (pixels)
7	Mask	Area (pixels)
7	Mask	Area (pixels)
7	Mask	Area (pixels)
7	Mask	Area (pixels)
7	Mask	Area (pixels)
7	Mask	Area (pixels)
7	Mask	Area (pixels)
7	Mask	Area (pixels)
7	Mask	Area (pixels)
7	Mask	Area (pixels)
7	Mask	Area (pixels)
7	Mask	Area (pixels)
7	Mask	Area (pixels)

7	Mask		Area (pixels)
7	Mask		Area (pixels)
7	Mask		Area (pixels)
7	Mask		Area (pixels)
7	Mask		Area (pixels)
7	Mask		Area (pixels)
7	Mask		Area (pixels)
7	Mask		Area (pixels)
7	Mask		Area (pixels)
7	Mask		Area (pixels)
7	Mask		Area (pixels)
7	Mask		Area (pixels)
7	Mask		Area (pixels)
7	Mask		Area (pixels)
7	Mask		Area (pixels)
7	Mask		Area (pixels)
7	Mask		Area (pixels)
7	Mask		Area (pixels)
7	Mask		Area (pixels)
8		0	12789
8		0	8419
8		0	4617
8		0	2591
8		0	5245
8		0	4038
8		0	5518
8		0	4833
8		0	7770
8		0	4631
8		0	4959
8		0	2954
8		0	5131
8		0	2051
8		0	3554
8		0	5416
8		0	2307
8		0	1003
8		0	873
8		0	5886
8		0	12769
8		0	10864
8		0	5668
8		0	6118
8		0	15628
8		0	18596
8		0	5538
8		0	5820

8	0	2742
8	0	6263
8	0	5643
8	0	6200
8	0	3747
8	0	3352
8	0	7043
8	0	3973
8	0	9930
8	0	6788
8	0	10852
8	0	5305
8	0	1796
8	0	4116
8	0	5092
8	0	5728
8	0	2798
8	0	4402
8	0	3822
8	0	4057
8	0	5720
8	0	6009
8	0	5698
8	0	3476
8	0	15914
8	0	3820
8	0	3429
8	0	6514
8	0	12412
8	0	9728
8	0	8145
8	0	2247
8	0	14153
8	0	14001
8	0	9463
8	0	13791
8	0	9353
8	0	8031
8	0	8466
8	0	8601
8	0	6227
8	0	7084
8	0	7067
8	0	5195
8	0	3869
8	0	8589
8	0	7589

8	0	3738
8	0	3862
8	0	5706
8	0	4941
8	0	3903
8	0	4349
8	0	6463
8	0	8244
8	0	12167
8	0	10260
8	0	5547
8	0	8885
8	0	5631
8	0	6310
8	0	7132
8	0	7256
8	0	9364
8	0	7284
8	0	10445
8	0	7312
8	0	7912
8	0	1059
8	0	20069
8	0	14734
8	0	13947
8	0	6884
8	0	16255
8	0	15276
8	0	10856
8	0	18704
8	0	7460
8	0	5199
8	0	7064
8	0	11301
8	0	6383
8	0	7107
8	0	4252
8	0	5215
8	0	14653
8	0	4942
8	0	11352
8	0	4336
8	0	9105
8	0	6003
8	0	17877
8	0	20999
8	0	10967

8	0	4930
8	0	6467
8	0	9681
8	0	22072
8	0	2112
8	0	4293
8	0	16876
8	0	28914
8	0	23145
8	0	6570
8	0	9933
8	0	5511
8	0	8054
8	0	12823
8	0	11651
8	0	6030
8	0	8031
8	0	3315
8	0	5334
8	0	10029
8	0	11810
8	0	9544
8	0	8615
8	0	15471
8	0	6760
8	0	30086
8	0	4948
8	0	4817
8	0	14304
8	0	3085
8	0	9735
8	0	2435
8	0	15511
8	0	5091
8	0	12854
8	0	2366
8	0	5698
8	0	3915
8	0	4459
8	0	7701
8	0	16139
8	0	2043
8	0	4896
8	0	3658
8	0	3434
8	0	8682
8	0	4703

8	0	15792
9		
9		
9		
9		
9		
9		
9		
9		
9		
9		
9		
9		
9		
9		
9		
9		
9		
9		
9		
9		
9		
9		
9		
9		
9		
9		
9		
9		
9		
9		
9		
9		
9		
9		
9		
9		
9		
9		
9		
9		
9		
9		
9		
9		
9		
9		

9
9
9
9
9
9
9
9
9
9
9
9
9
9
9
9
9
9
9
9
9
9
9
9
9
9
9
9
9
9
9
9
9
9
9
9
9
9
9
9
9
9
9
9
9
9
9

9
9
9
9
9
9
9
9
9
9
9
9
9
9
9
9
9
9
9
9
9
9
9
9
9
9
9
9
9
9
9
9
9
9
9
9
9
9
9
9
9
9
9
9
9
9
9

9
9
9
9
9
9
9
9
9
9
9
9
9
9
9
9
9
9
9
9
9
9
9
9
9
9
9
9
9
9

g identical, import all images into SlideBook (Intelligent Imaging Innovations, Denver, Colorado; Colin Mon

Positive pixels	% positive pixels	ave/sld
12789	0.67	0.31
8419	0.44	
4617	0.24	
2591	0.13	
5245	0.27	
4038	0.21	
5518	0.29	
4833	0.25	
7770	0.40	
4631	0.24	
4959	0.26	0.18
2954	0.15	
5131	0.27	
2051	0.11	
3554	0.19	
5416	0.28	
2307	0.12	
1003	0.05	
873	0.05	
5886	0.31	
12769	0.67	0.47
10864	0.57	
5668	0.30	
6118	0.32	
15628	0.81	
18596	0.97	
5538	0.29	
5820	0.30	
2742	0.14	
6263	0.33	
5643	0.29	0.33
6200	0.32	
3747	0.20	
3352	0.17	
7043	0.37	
3973	0.21	
9930	0.52	
6788	0.35	
10852	0.57	
5305	0.28	
1796	0.09	0.23
4116	0.21	
5092	0.27	

Summary	
Image ID	ave/sld
604	0.31
605	0.18
606	0.47
616	0.33
620	0.23
737	0.37
938	0.52
939	0.28
940	0.39
955	0.52
956	0.55
957	0.44
TPN-349	0.66
TPN-350	0.50
TPN-351	0.56
TPN-364	0.39
TPN-365	0.37

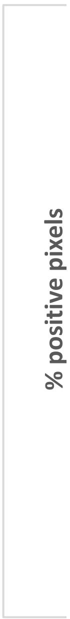

5728	0.30	
2798	0.15	
4402	0.23	
3822	0.20	
4057	0.21	
5720	0.30	
6009	0.31	
5698	0.30	0.37
3476	0.18	
15914	0.83	
3820	0.20	
3429	0.18	
6514	0.34	
12412	0.65	
9728	0.51	
8145	0.42	
2247	0.12	
14153	0.74	0.52
14001	0.73	
9463	0.49	
13791	0.72	
9353	0.49	
8031	0.42	
8466	0.44	
8601	0.45	
6227	0.32	
7084	0.37	
7067	0.37	0.28
5195	0.27	
3869	0.20	
8589	0.45	
7589	0.40	
3738	0.19	
3862	0.20	
5706	0.30	
4941	0.26	
3903	0.20	
4349	0.23	0.39
6463	0.34	
8244	0.43	
12167	0.63	
10260	0.53	
5547	0.29	
8885	0.46	
5631	0.29	
6310	0.33	
7132	0.37	

7256	0.38	0.52
9364	0.49	
7284	0.38	
10445	0.54	
7312	0.38	
7912	0.41	
1059	0.06	
20069	1.05	
14734	0.77	
13947	0.73	
6884	0.36	0.55
16255	0.85	
15276	0.80	
10856	0.57	
18704	0.97	
7460	0.39	
5199	0.27	
7064	0.37	
11301	0.59	
6383	0.33	
7107	0.37	0.44
4252	0.22	
5215	0.27	
14653	0.76	
4942	0.26	
11352	0.59	
4336	0.23	
9105	0.47	
6003	0.31	
17877	0.93	
20999	1.09	0.66
10967	0.57	
4930	0.26	
6467	0.34	
9681	0.50	
22072	1.15	
2112	0.11	
4293	0.22	
16876	0.88	
28914	1.51	
23145	1.21	0.50
6570	0.34	
9933	0.52	
5511	0.29	
8054	0.42	
12823	0.67	
11651	0.61	

6030	0.31	
8031	0.42	
3315	0.17	
5334	0.28	0.56
10029	0.52	
11810	0.62	
9544	0.50	
8615	0.45	
15471	0.81	
6760	0.35	
30086	1.57	
4948	0.26	
4817	0.25	
14304	0.75	0.39
3085	0.16	
9735	0.51	
2435	0.13	
15511	0.81	
5091	0.27	
12854	0.67	
2366	0.12	
5698	0.30	
3915	0.20	
4459	0.23	0.37
7701	0.40	
16139	0.84	
2043	0.11	
4896	0.26	
3658	0.19	
3434	0.18	
8682	0.45	
4703	0.24	
15792	0.82	

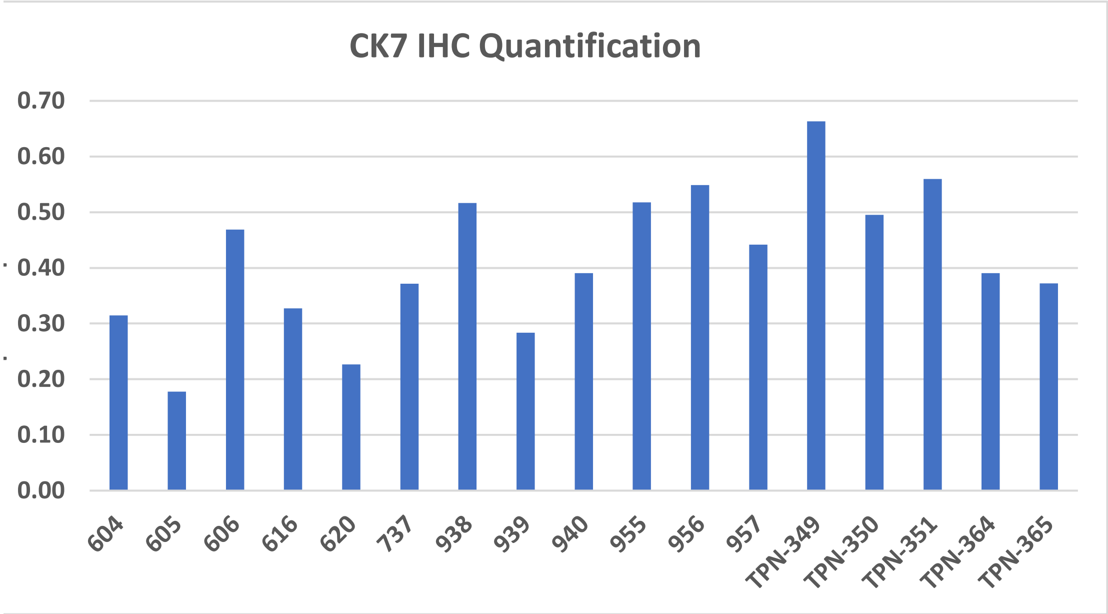

CK7 IHC Quantification
0.70
0.60
0.50
0.40
0.30
0.20
0.10
0.00
604
605
606
616
620
737
938
939
940
955
956
957
TPN-349
TPN-350
TPN-351
TPN-364
TPN-365

Statistics for Circadian DSS-Pn paper

AST

Tukey's multiple compa	Mean Diff.	95.00% CI	Below thre:	Summary	Adjusted P Value
Chow vs. IL1^{KO} Chow	8.222	-46.21 to 6	No	ns	0.9739
Chow vs. DSS-PN	-103.8	-144.9 to -6	Yes	****	<0.0001
Chow vs. DSS-PN/IL1^{K}	-8.978	-54.52 to 3	No	ns	0.945
IL1^{KO} Chow vs. DSS-PN	-112	-168.3 to -5	Yes	***	0.0001
IL1^{KO} Chow vs. DSS-PN	-17.2	-76.82 to 4	No	ns	0.8502
DSS-PN vs. DSS-PN/IL	94.8	47.00 to 14	Yes	***	0.0001

Tukey's multiple compa	Mean Diff.	95.00% CI	Below thre:	Summary	Adjusted P Value
Chow vs. TNFR$^{-/-}$	9.222	-31.23 to 4	No	ns	0.9217
Chow vs. DSS-PN	-103.8	-142.5 to -6	Yes	****	<0.0001
Chow vs. DSS-PN/TNF	0.5556	-39.90 to 4	No	ns	>0.9999
TNFR$^{-/-}$ vs. DSS-PN	-113	-155.7 to -7	Yes	****	<0.0001
TNFR$^{-/-}$ vs. DSS-PN/TN	-8.667	-52.98 to 3	No	ns	0.9485
DSS-PN vs. DSS-PN/TI	104.3	61.63 to 14	Yes	****	<0.0001

ALT

Tukey's multiple compa	Mean Diff.	95.00% CI	Below thre:	Summary	Adjusted P Value
Chow vs. IL1^{KO} Chow	-12.33	-28.83 to 4	No	ns	0.1897
Chow vs. DSS-PN	-23.19	-35.66 to -1	Yes	***	0.0002
Chow vs. DSS-PN/IL1^{K}	-17.93	-31.74 to -4	Yes	**	0.0082
IL1^{KO} Chow vs. DSS-PN	-10.86	-27.94 to 6	No	ns	0.3119
IL1^{KO} Chow vs. DSS-PN	-5.6	-23.68 to 1	No	ns	0.8216
DSS-PN vs. DSS-PN/IL	5.257	-9.236 to 1	No	ns	0.7425

Tukey's multiple compa	Mean Diff.	95.00% CI	Below thre:	Summary	Adjusted P Value
Chow vs. TNFR$^{-/-}$	2.667	-8.905 to 1	No	ns	0.9194
Chow vs. DSS-PN	-23.19	-34.25 to -1	Yes	****	<0.0001
Chow vs. DSS-PN/TNF	5.333	-6.238 to 1	No	ns	0.5892
TNFR$^{-/-}$ vs. DSS-PN	-25.86	-38.07 to -1	Yes	****	<0.0001
TNFR$^{-/-}$ vs. DSS-PN/TN	2.667	-10.01 to 1	No	ns	0.937
DSS-PN vs. DSS-PN/TI	28.52	16.31 to 40	Yes	****	<0.0001

Bile acids

Tukey's multiple compa	Mean Diff.	95.00% CI	Below thre:	Summary	Adjusted P Value
Chow vs. IL1^{KO} Chow	-1.502	-4.348 to 1	No	ns	0.4716
Chow vs. DSS-PN	-5.655	-7.757 to -3	Yes	****	<0.0001
Chow vs. DSS-PN/IL1^{K}	-0.2237	-2.494 to 2	No	ns	0.9925
IL1^{KO} Chow vs. DSS-PN	-4.153	-6.999 to -1	Yes	**	0.0029
IL1^{KO} Chow vs. DSS-PN	1.278	-1.694 to 4	No	ns	0.6343
DSS-PN vs. DSS-PN/IL	5.431	3.161 to 7.	Yes	****	<0.0001

Tukey's multiple compa	Mean Diff.	95.00% CI	Below thre:	Summary	Adjusted P Value
Chow vs. TNFR$^{-/-}$	0.4113	-1.469 to 2	No	ns	0.93
Chow vs. DSS-PN	-5.655	-7.396 to -3	Yes	****	<0.0001
Chow vs. DSS-PN/TNF	0.6046	-1.276 to 2	No	ns	0.8116
TNFR$^{-/-}$ vs. DSS-PN	-6.066	-7.947 to -4	Yes	****	<0.0001
TNFR$^{-/-}$ vs. DSS-PN/TN	0.1933	-1.817 to 2	No	ns	0.9933
DSS-PN vs. DSS-PN/TI	6.26	4.379 to 8.	Yes	****	<0.0001

F4/80

Tukey's multiple compa	Mean Diff.	95.00% CI	Below thre:	Summary	Adjusted P Value
**Chow** vs. **IL1^{KO}**	0.3675	-0.1954 to	No	ns	0.2581
**Chow** vs. **DSS-PN**	-2.344	-2.865 to -1	Yes	****	<0.0001
**Chow** vs. **DSS-PN/IL1^{K}**	0.1875	-0.3337 to	No	ns	0.7066
**IL1^{KO}** vs. **DSS-PN**	-2.712	-3.275 to -2	Yes	****	<0.0001
**IL1^{KO}** vs. **DSS-PN/IL1^{K}**	-0.18	-0.7429 to	No	ns	0.7728
**DSS-PN** vs. **DSS-PN/IL**	2.532	2.011 to 3.(	Yes	****	<0.0001

Tukey's multiple compa	Mean Diff.	95.00% CI	Below thre:	Summary	Adjusted P Value
**Chow** vs. **Chow TNFR**	0.1492	-0.7494 to	No	ns	0.9575
**Chow** vs. **DSS-PN**	-2.344	-3.176 to -1	Yes	****	<0.0001
**Chow** vs. **DSS-PN/TNF**	-0.2758	-1.108 to 0	No	ns	0.7537
**Chow TNFR^{KO}** vs. **DSS**	-2.493	-3.392 to -1	Yes	****	<0.0001
**Chow TNFR^{KO}** vs. **DSS**	-0.425	-1.324 to 0	No	ns	0.5115
**DSS-PN** vs. **DSS-PN/TI**	2.068	1.236 to 2.9	Yes	****	<0.0001

CK7

Tukey's multiple compa	Mean Diff.	95.00% CI	Below thre:	Summary	Adjusted P Value
Chow vs. DSS-PN	0.1067	-0.09727 to	No	ns	0.3945
Chow vs. IL1^{KO} Chow	0.1267	-0.07727 to	No	ns	0.2678
Chow vs. IL1^{KO} DSS-PN	-0.07	-0.2739 to	No	ns	0.6999
DSS-PN vs. IL1^{KO} Chow	0.02	-0.1839 to	No	ns	0.9885
DSS-PN vs. IL1^{KO} DSS-	-0.1767	-0.3806 to	No	ns	0.0915
IL1^{KO} Chow vs. IL1^{KO} DS	-0.1967	-0.4006 to	No	ns	0.0587

Tukey's multiple compa	Mean Diff.	95.00% CI	Below thre:	Summary	Adjusted P Value
Chow vs. DSS-PN	0.1067	-0.1868 to	No	ns	0.6441
Chow vs. TNFR^{KO}CHOV	0.2233	-0.1048 to	No	ns	0.1987
Chow vs. TNFR^{KO}DSS-	0.1833	-0.1101 to	No	ns	0.2512
DSS-PN vs. TNFR^{KO}CH	0.1167	-0.2114 to	No	ns	0.6585
DSS-PN vs. TNFR^{KO}DS	0.07667	-0.2168 to	No	ns	0.8226
TNFR^{KO}CHOW vs. TNF	-0.04	-0.3681 to	No	ns	0.9761

Ki67

Tukey's multiple compa	Mean Diff.	95.00% CI	Below thres	Summary	Adjusted P Value
Chow vs. DSS-PN	5.411	-6.004 to 1	No	ns	0.6179
Chow vs. IL1^{KO} Chow	2.411	-9.004 to 1	No	ns	0.9771
Chow vs. IL1^{KO} DSS-PN	4.289	-7.126 to 1	No	ns	0.7991
Chow vs. TNFR^{KO}CHOV	7.322	-4.093 to 1	No	ns	0.324
Chow vs. TNFR^{KO}DSS-	-2.789	-14.20 to 8	No	ns	0.9579
DSS-PN vs. IL1^{KO} Chow	-3	-14.42 to 8	No	ns	0.9437
DSS-PN vs. IL1^{KO} DSS-	-1.122	-12.54 to 1	No	ns	0.9993
DSS-PN vs. TNFR^{KO}CH	1.911	-9.504 to 1	No	ns	0.9918
DSS-PN vs. TNFR^{KO}DS	-8.2	-19.62 to 3	No	ns	0.2257
IL1^{KO} Chow vs. IL1^{KO} DS	1.878	-9.537 to 1	No	ns	0.9924
IL1^{KO} Chow vs. TNFR^{KO}	4.911	-6.504 to 1	No	ns	0.7017
IL1^{KO} Chow vs. TNFR^{KO}	-5.2	-16.62 to 6	No	ns	0.6536
IL1^{KO} DSS-PN vs. TNFR	3.033	-8.382 to 1	No	ns	0.9412
IL1^{KO} DSS-PN vs. TNFR	-7.078	-18.49 to 4	No	ns	0.3561
TNFR^{KO}CHOW vs. TNF	-10.11	-21.53 to 1	No	ns	0.0943

Westerns

reverba

Tukey's multiple compa	Mean Diff.	95.00% CI	Below thres	Summary	Adjusted P Value
CHOW vs. DSS-PN	0.2008	-0.2140 to	No	ns	0.4546
CHOW vs. IL1^{KO}CHOW	-0.2407	-0.6555 to	No	ns	0.3161
CHOW vs. IL1^{KO}DSS-P	-0.4081	-0.8229 to	No	ns	0.0538
DSS-PN vs. IL1^{KO}CHOV	-0.4415	-0.8563 to	Yes	*	0.0375
DSS-PN vs. IL1^{KO}DSS-	-0.6088	-1.024 to -(	Yes	**	0.0067
IL1^{KO}CHOW vs. IL1^{KO}D	-0.1673	-0.5822 to	No	ns	0.5922

Tukey's multiple compa	Mean Diff.	95.00% CI	Below thres	Summary	Adjusted P Value
CHOW vs. DSS-PN	0.3842	0.01341 to	Yes	*	0.0425
CHOW vs. TNFR^{KO} CH	0.2303	-0.1406 to	No	ns	0.268
CHOW vs. TNFR^{KO} DS	0.1845	-0.1863 to	No	ns	0.4332
DSS-PN vs. TNFR^{KO} CI	-0.154	-0.5248 to	No	ns	0.5713
DSS-PN vs. TNFR^{KO} DS	-0.1997	-0.5706 to	No	ns	0.3719
TNFR^{KO} CHOW vs. TN	-0.04576	-0.4166 to	No	ns	0.9777

Nfil3

Tukey's multiple compa	Mean Diff.	95.00% CI	Below thres	Summary	Adjusted P Value
CHOW vs. DSS-PN	0.0948	-0.3979 to	No	ns	0.9241
CHOW vs. IL1^{KO}CHOW	0.4396	-0.05312 tc	No	ns	0.0814
CHOW vs. IL1^{KO}DSS-P	0.3311	-0.1616 to	No	ns	0.2162
DSS-PN vs. IL1^{KO}CHOV	0.3448	-0.1479 to	No	ns	0.1919
DSS-PN vs. IL1^{KO}DSS-	0.2363	-0.2564 to	No	ns	0.4616

IL1^{KO}CHOW vs. IL1^{KO}D	-0.1084	-0.6011 to	No	ns	0.8924

Tukey's multiple compa	Mean Diff.	95.00% CI	Below thre	Summary	Adjusted P Value
CHOW vs. DSS-PN	0.1116	-0.5399 to	No	ns	0.9444
CHOW vs. TNFR^{KO} CH	-0.1387	-0.7901 to	No	ns	0.9012
CHOW vs. TNFR^{KO} DS	0.04872	-0.6028 to	No	ns	0.9948
DSS-PN vs. TNFR^{KO} C	-0.2502	-0.9017 to	No	ns	0.6268
DSS-PN vs. TNFR^{KO} D	-0.06284	-0.7143 to	No	ns	0.989
TNFR^{KO} CHOW vs. TN	0.1874	-0.4641 to	No	ns	0.7948

Bmal

Tukey's multiple compa	Mean Diff.	95.00% CI	Below thre	Summary	Adjusted P Value
CHOW vs. DSS-PN	0.2521	0.01938 to	Yes	*	0.0344
CHOW vs. IL1^{KO}CHOW	-0.01028	-0.2430 to	No	ns	0.9989
CHOW vs. IL1^{KO}DSS-P	-0.2431	-0.4758 to	Yes	*	0.041
DSS-PN vs. IL1^{KO}CHO	-0.2624	-0.4951 to	Yes	*	0.0283
DSS-PN vs. IL1^{KO}DSS-	-0.4952	-0.7279 to	Yes	***	0.0006
IL1^{KO}CHOW vs. IL1^{KO}D	-0.2328	-0.4655 to	Yes	*	0.05

Tukey's multiple compa	Mean Diff.	95.00% CI	Below thre	Summary	Adjusted P Value
CHOW vs. DSS-PN	0.5441	0.3334 to (	Yes	***	0.0002
CHOW vs. TNFR^{KO} CH	0.05878	-0.1520 to	No	ns	0.8088
CHOW vs. TNFR^{KO} DS	-0.2156	-0.4264 to	Yes	*	0.0451
DSS-PN vs. TNFR^{KO} C	-0.4853	-0.6961 to	Yes	***	0.0004
DSS-PN vs. TNFR^{KO} D	-0.7597	-0.9705 to	Yes	****	<0.0001
TNFR^{KO} CHOW vs. TN	-0.2744	-0.4851 to	Yes	*	0.0133

RORa

Tukey's multiple compa	Mean Diff.	95.00% CI	Below thre	Summary	Adjusted P Value
CHOW vs. DSS-PN	-2.146	-5.098 to 0	No	ns	0.1704
CHOW vs. IL1^{KO}CHOW	-0.2892	-3.241 to 2	No	ns	0.9885
CHOW vs. IL1^{KO}DSS-P	-0.1546	-3.106 to 2	No	ns	0.9982
DSS-PN vs. IL1^{KO}CHO	1.857	-1.095 to 4	No	ns	0.2591
DSS-PN vs. IL1^{KO}DSS-	1.992	-0.9602 to	No	ns	0.2138
IL1^{KO}CHOW vs. IL1^{KO}D	0.1346	-2.817 to 3	No	ns	0.9988

Tukey's multiple compa	Mean Diff.	95.00% CI	Below thre	Summary	Adjusted P Value
CHOW vs. DSS-PN	-2.063	-5.402 to 1	No	ns	0.2716
CHOW vs. TNFR^{KO} CH	-0.249	-3.588 to 3	No	ns	0.9948
CHOW vs. TNFR^{KO} DS	-8.579	-11.92 to -5	Yes	***	0.0002
DSS-PN vs. TNFR^{KO} C	1.814	-1.525 to 5	No	ns	0.3655
DSS-PN vs. TNFR^{KO} D	-6.516	-9.855 to -3	Yes	**	0.0011
TNFR^{KO} CHOW vs. TN	-8.33	-11.67 to -4	Yes	***	0.0002

Dbp

Tukey's multiple compa	Mean Diff.	95.00% CI	Below thres	Summary	Adjusted P Value
CHOW vs. DSS-PN	0.2691	0.05988 to	Yes	*	0.0142
CHOW vs. IL1^{KO}CHOW	0.03334	-0.1759 to	No	ns	0.9543
CHOW vs. IL1^{KO}DSS-P	0.2802	0.07100 to	Yes	*	0.0114
DSS-PN vs. IL1^{KO}CHOV	-0.2357	-0.4449 to	Yes	*	0.0284
DSS-PN vs. IL1^{KO}DSS-	0.01112	-0.1981 to	No	ns	0.9981
IL1^{KO}CHOW vs. IL1^{KO}D	0.2469	0.03766 to	Yes	*	0.0225

Tukey's multiple compa	Mean Diff.	95.00% CI	Below thres	Summary	Adjusted P Value
CHOW vs. DSS-PN	0.247	-0.03107 tc	No	ns	0.0829
CHOW vs. TNFR^{KO} CH	0.07693	-0.2012 to	No	ns	0.8124
CHOW vs. TNFR^{KO} DS	-0.4713	-0.7494 to	Yes	**	0.0028
DSS-PN vs. TNFR^{KO} CI	-0.1701	-0.4482 to	No	ns	0.2784
DSS-PN vs. TNFR^{KO} D:	-0.7184	-0.9965 to	Yes	***	0.0002
TNFR^{KO} CHOW vs. TN	-0.5482	-0.8264 to	Yes	**	0.001

Per2

Tukey's multiple compa	Mean Diff.	95.00% CI	Below thres	Summary	Adjusted P Value
CHOW vs. DSS-PN	0.6055	-0.3281 to	No	ns	0.2388
CHOW vs. IL1^{KO}CHOW	-0.482	-1.416 to 0	No	ns	0.4045
CHOW vs. IL1^{KO}DSS-P	-0.3931	-1.327 to 0	No	ns	0.5611
DSS-PN vs. IL1^{KO}CHOV	-1.087	-2.021 to -(	Yes	*	0.024
DSS-PN vs. IL1^{KO}DSS-	-0.9986	-1.932 to -(	Yes	*	0.0366
IL1^{KO}CHOW vs. IL1^{KO}D	0.08893	-0.8447 to	No	ns	0.9894

Tukey's multiple compa	Mean Diff.	95.00% CI	Below thres	Summary	Adjusted P Value
CHOW vs. DSS-PN	0.6698	0.2676 to 1	Yes	**	0.0031
CHOW vs. TNFR^{KO} CH	0.2908	-0.1115 to	No	ns	0.1735
CHOW vs. TNFR^{KO} DS	0.2446	-0.1576 to	No	ns	0.2825
DSS-PN vs. TNFR^{KO} CI	-0.379	-0.7813 to	No	ns	0.0649
DSS-PN vs. TNFR^{KO} D:	-0.4252	-0.8274 to	Yes	*	0.0387
TNFR^{KO} CHOW vs. TN	-0.04615	-0.4484 to	No	ns	0.9819

Mtnr1a

Tukey's multiple compa	Mean Diff.	95.00% CI	Below thres	Summary	Adjusted P Value
CHOW vs. DSS-PN	-0.1206	-0.3882 to	No	ns	0.5099
CHOW vs. IL1^{KO}CHOW	-0.3317	-0.5993 to	Yes	*	0.0174
CHOW vs. IL1^{KO}DSS-P	-0.5808	-0.8484 to	Yes	***	0.0005
DSS-PN vs. IL1^{KO}CHOV	-0.2111	-0.4787 to	No	ns	0.1296
DSS-PN vs. IL1^{KO}DSS-	-0.4602	-0.7278 to	Yes	**	0.0025
IL1^{KO}CHOW vs. IL1^{KO}D	-0.2492	-0.5168 to	No	ns	0.0683

Tukey's multiple compa	Mean Diff.	95.00% CI	Below thre:	Summary	Adjusted P Value
CHOW vs. DSS-PN	-0.07808	-0.6281 to	No	ns	0.9668
CHOW vs. TNFR^{KO} CH	0.1297	-0.4202 to	No	ns	0.872
CHOW vs. TNFR^{KO} DS	0.3963	-0.1537 to	No	ns	0.1753
DSS-PN vs. TNFR^{KO} Cl	0.2078	-0.3422 to	No	ns	0.638
DSS-PN vs. TNFR^{KO} D!	0.4744	-0.07557 tc	No	ns	0.093
TNFR^{KO} CHOW vs. TN	0.2666	-0.2834 to	No	ns	0.4535

Mtnr1b

Tukey's multiple compa	Mean Diff.	95.00% CI	Below thre:	Summary	Adjusted P Value
CHOW vs. DSS-PN	0.9259	0.5772 to 1	Yes	***	0.0001
CHOW vs. IL1^{KO}CHOW	-0.3833	-0.7320 to	Yes	*	0.0321
CHOW vs. IL1^{KO}DSS-P	0.4209	0.07217 to	Yes	*	0.02
DSS-PN vs. IL1^{KO}CHO\	-1.309	-1.658 to -(	Yes	****	<0.0001
DSS-PN vs. IL1^{KO}DSS-	-0.505	-0.8537 to	Yes	**	0.0072
IL1^{KO}CHOW vs. IL1^{KO}D	0.8042	0.4555 to 1	Yes	***	0.0004

Tukey's multiple compa	Mean Diff.	95.00% CI	Below thre:	Summary	Adjusted P Value
CHOW vs. DSS-PN	0.8483	0.4876 to 1	Yes	***	0.0003
CHOW vs. TNFR^{KO} CH	0.4209	0.06019 to	Yes	*	0.0238
CHOW vs. TNFR^{KO} DS	-0.2255	-0.5861 to	No	ns	0.2633
DSS-PN vs. TNFR^{KO} Cl	-0.4274	-0.7881 to	Yes	*	0.022
DSS-PN vs. TNFR^{KO} D!	-1.074	-1.434 to -(	Yes	****	<0.0001
TNFR^{KO} CHOW vs. TN	-0.6463	-1.007 to -(	Yes	**	0.0019

i.p. Injections

AST

Column B	IL-1β	Column C	TNFα
vs.	vs.	vs.	vs.
Column A	Chow	Column A	Chow
Unpaired t test		Unpaired t test	
P value	0.0127	P value	0.0092
P value summary	*	P value su	**
Significantly different (P	Yes	Significantl	Yes
One- or two-tailed P val	Two-tailed	One- or tw	Two-tailed

ALT

Column B	IL-1β	Column C	TNFα
vs.	vs.	vs.	vs.
Column A	Chow	Column A	Chow
Unpaired t test		Unpaired t test	
P value	0.01	P value	0.0075

P value summary	*	P value su	**
Significantly different (P	Yes	Significantl	Yes
One- or two-tailed P val	Two-tailed	One- or tw	Two-tailed
qPCR			
Nr1d1			
Column B	IL-1β	Column C	TNFα
vs.	vs.	vs.	vs.
Column A	Chow	Column A	Chow
Unpaired t test		Unpaired t test	
P value	0.0469	P value	0.6383
P value summary	*	P value su	ns
Significantly different (P	Yes	Significantl	No
One- or two-tailed P val	Two-tailed	One- or tw	Two-tailed
Nr1f1			
Column B	IL-1β	Column C	TNFα
vs.	vs.	vs.	vs.
Column A	Chow	Column A	Chow
Unpaired t test		Unpaired t test	
P value	0.0013	P value	0.0197
P value summary	**	P value su	*
Significantly different (P	Yes	Significantl	Yes
One- or two-tailed P val	Two-tailed	One- or tw	Two-tailed
Nfil3			
Column C	TNFα	Column B	IL-1β
vs.	vs.	vs.	vs.
Column A	Chow	Column A	Chow
Unpaired t test		Unpaired t test	
P value	0.4669	P value	0.8232
P value summary	ns	P value su	ns
Significantly different (P	No	Significantl	No
One- or two-tailed P val	Two-tailed	One- or tw	Two-tailed
Bmal			
Column B	IL-1β	Column C	TNFα
vs.	vs.	vs.	vs.
Column A	Chow	Column A	Chow
Unpaired t test		Unpaired t test	
P value	0.0009	P value	0.0002
P value summary	***	P value su	***
Significantly different (P	Yes	Significantl	Yes

One- or two-tailed P val	Two-tailed	One- or tw	Two-tailed
Clcok			
Column B	IL-1β	Column C	TNFα
vs.	vs.	vs.	vs.
Column A	Chow	Column A	Chow
Unpaired t test		Unpaired t test	
P value	0.0222	P value	0.0017
P value summary	*	P value su	**
Significantly different (P	Yes	Significantl	Yes
One- or two-tailed P val	Two-tailed	One- or tw	Two-tailed
Dbp			
Column B	IL-1β	Column C	TNFα
vs.	vs.	vs.	vs.
Column A	Chow	Column A	Chow
Unpaired t test		Unpaired t test	
P value	0.0208	P value	0.05
P value summary	*	P value su	*
Significantly different (P	Yes	Significantl	No
One- or two-tailed P val	Two-tailed	One- or tw	Two-tailed
Per1			
Column B	IL-1β	Column C	TNFα
vs.	vs.	vs.	vs.
Column A	Chow	Column A	Chow
Unpaired t test		Unpaired t test	
P value	0.0844	P value	0.3514
P value summary	ns	P value su	ns
Significantly different (P	No	Significantl	No
One- or two-tailed P val	Two-tailed	One- or tw	Two-tailed
Per2			
Column B	IL-1β	Column C	TNFα
vs.	vs.	vs.	vs.
Column A	Chow	Column A	Chow
Unpaired t test		Unpaired t test	
P value	0.0021	P value	0.0066
P value summary	**	P value su	**
Significantly different (P	Yes	Significantl	Yes
One- or two-tailed P val	Two-tailed	One- or tw	Two-tailed
Cry1			

Column B	IL-1β	Column C	TNFα
vs.	vs.	vs.	vs.
Column A	Chow	Column A	Chow
Unpaired t test		Unpaired t test	
P value	0.8403	P value	0.2558
P value summary	ns	P value sui	ns
Significantly different (P	No	Significantl	No
One- or two-tailed P val	Two-tailed	One- or tw(	Two-tailed

Cry2

Column B	IL-1β	Column B	IL-1β
vs.	vs.	vs.	vs.
Column A	Chow	Column A	Chow
Unpaired t test		Unpaired t test	
P value	0.2854	P value	0.2854
P value summary	ns	P value sui	ns
Significantly different (P	No	Significantl	No
One- or two-tailed P val	Two-tailed	One- or tw(	Two-tailed

1-Dec

Column B	IL-1β	Column C	TNFα
vs.	vs.	vs.	vs.
Column A	Chow	Column A	Chow
Unpaired t test		Unpaired t test	
P value	0.2854	P value	0.405
P value summary	ns	P value sui	ns
Significantly different (P	No	Significantl	No
One- or two-tailed P val	Two-tailed	One- or tw(	Two-tailed

Dec2

Column B	IL-1β	Column C	TNFα
vs.	vs.	vs.	vs.
Column A	Chow	Column A	Chow
Unpaired t test		Unpaired t test	
P value	0.0009	P value	0.0099
P value summary	***	P value sui	**
Significantly different (P	Yes	Significantl	Yes
One- or two-tailed P val	Two-tailed	One- or tw(	Two-tailed

Knockouts

nr1d1

Tukey's multiple compa	Mean Diff.	95.00% CI	Below thre:	Summary	Adjusted P Value
Chow vs. DSS-PN	0.08618	-4.071 to 4	No	ns	>0.9999

Chow vs. IL1^{KO} Chow	0.4299	-4.060 to 4	No	ns	0.9912
Chow vs. IL1^{KO} DSS-PN	-6.915	-11.07 to -	Yes	**	0.0019
DSS-PN vs. IL1^{KO} Chow	0.3437	-4.146 to 4	No	ns	0.9954
DSS-PN vs. IL1^{KO} DSS-	-7.001	-11.16 to -	Yes	**	0.0017
IL1^{KO} Chow vs. IL1^{KO} DS	-7.345	-11.83 to -	Yes	**	0.0022

Tukey's multiple compa	Mean Diff.	95.00% CI	Below thre	Summary	Adjusted P Value
CHOW vs. DSS-PN	0.08618	-1.192 to 1	No	ns	0.997
CHOW vs. TNFR^{KO} CH	0.887	-0.3912 to	No	ns	0.2206
CHOW vs. TNFR^{KO} DS	1.226	-0.05167 t	No	ns	0.0615
DSS-PN vs. TNFR^{KO} Cl	0.8008	-0.4774 to	No	ns	0.2945
DSS-PN vs. TNFR^{KO} DS	1.14	-0.1379 to	No	ns	0.0863
TNFR^{KO} CHOW vs. TN	0.3395	-0.9387 to	No	ns	0.8582

ROR

Tukey's multiple compa	Mean Diff.	95.00% CI	Below thre	Summary	Adjusted P Value
Chow vs. DSS-PN	0.3487	0.01038 to	Yes	*	0.0429
Chow vs. IL1^{KO} Chow	-0.09684	-0.4623 to	No	ns	0.8541
Chow vs. IL1^{KO} DSS-PN	-0.1379	-0.4763 to	No	ns	0.6237
DSS-PN vs. IL1^{KO} Chow	-0.4455	-0.8110 to	Yes	*	0.0166
DSS-PN vs. IL1^{KO} DSS-	-0.4866	-0.8250 to	Yes	**	0.0056
IL1^{KO} Chow vs. IL1^{KO} DS	-0.0411	-0.4065 to	No	ns	0.9859

Tukey's multiple compa	Mean Diff.	95.00% CI	Below thre	Summary	Adjusted P Value
Chow vs. DSS-PN	0.3487	0.01038 to	Yes	*	0.0429
Chow vs. IL1^{KO} Chow	-0.09684	-0.4623 to	No	ns	0.8541
Chow vs. IL1^{KO} DSS-PN	-0.1379	-0.4763 to	No	ns	0.6237
DSS-PN vs. IL1^{KO} Chow	-0.4455	-0.8110 to	Yes	*	0.0166
DSS-PN vs. IL1^{KO} DSS-	-0.4866	-0.8250 to	Yes	**	0.0056
IL1^{KO} Chow vs. IL1^{KO} DS	-0.0411	-0.4065 to	No	ns	0.9859

Nfil3

Tukey's multiple compa	Mean Diff.	95.00% CI	Below thre	Summary	Adjusted P Value
Chow vs. DSS-PN	-3.044	-7.363 to 1	No	ns	0.2058
Chow vs. IL1^{KO} Chow	-0.7146	-5.380 to 3	No	ns	0.966
Chow vs. IL1^{KO} DSS-PN	-1.498	-5.817 to 2	No	ns	0.7285
DSS-PN vs. IL1^{KO} Chow	2.33	-2.335 to 6	No	ns	0.468
DSS-PN vs. IL1^{KO} DSS-	1.546	-2.773 to 5	No	ns	0.7095
IL1^{KO} Chow vs. IL1^{KO} DS	-0.7832	-5.448 to 3	No	ns	0.9561

Tukey's multiple compa	Mean Diff.	95.00% CI	Below thre	Summary	Adjusted P Value
CHOW vs. DSS-PN	-3.044	-9.013 to 2	No	ns	0.4595
CHOW vs. TNFR^{KO} CH	-0.01088	-5.980 to 5	No	ns	>0.9999

CHOW vs. TNFR^{KO} DS	-2.99	-8.959 to 2	No	ns	0.4741
DSS-PN vs. TNFR^{KO} C	3.033	-2.936 to 9	No	ns	0.4624
DSS-PN vs. TNFR^{KO} D	0.05392	-5.915 to 6	No	ns	>0.9999
TNFR^{KO} CHOW vs. TN	-2.979	-8.948 to 2	No	ns	0.477

Bmal

Tukey's multiple compa	Mean Diff.	95.00% CI	Below thre	Summary	Adjusted P Value
Chow vs. DSS-PN	0.4376	-0.2958 to	No	**	0.0063
Chow vs. IL1^{KO} Chow	-1.332	-2.124 to -(	Yes	**	0.0018
Chow vs. IL1^{KO} DSS-PN	-0.1228	-0.8561 to	No	ns	0.9565
DSS-PN vs. IL1^{KO} Chow	-1.77	-2.562 to -(	Yes	***	0.0002
DSS-PN vs. IL1^{KO} DSS-	-0.5604	-1.294 to 0	No	ns	0.1572
IL1^{KO} Chow vs. IL1^{KO} D	1.209	0.4171 to 2	Yes	**	0.0037

Tukey's multiple compa	Mean Diff.	95.00% CI	Below thre	Summary	Adjusted P Value
CHOW vs. DSS-PN	0.657	-0.03206 tc	No	*	0.0154
CHOW vs. TNFR^{KO} CH	-0.06539	-0.7544 to	No	ns	0.9918
CHOW vs. TNFR^{KO} DS	-0.2092	-0.8983 to	No	ns	0.8044
DSS-PN vs. TNFR^{KO} C	-0.7224	-1.411 to -(	Yes	*	0.039
DSS-PN vs. TNFR^{KO} D	-0.8662	-1.555 to -(	Yes	*	0.0132
TNFR^{KO} CHOW vs. TN	-0.1438	-0.8329 to	No	ns	0.9238

Clock

Tukey's multiple compa	Mean Diff.	95.00% CI	Below thre	Summary	Adjusted P Value
Chow vs. DSS-PN	-0.3865	-1.611 to 0	No	ns	0.7793
Chow vs. IL1^{KO} Chow	-1.195	-2.517 to 0	No	ns	0.0806
Chow vs. IL1^{KO} DSS-PN	-2.258	-3.482 to -1	Yes	***	0.0008
DSS-PN vs. IL1^{KO} Chow	-0.8086	-2.131 to 0	No	ns	0.3065
DSS-PN vs. IL1^{KO} DSS-	-1.871	-3.096 to -(	Yes	**	0.0036
IL1^{KO} Chow vs. IL1^{KO} D	-1.063	-2.385 to 0	No	ns	0.1306

Tukey's multiple compa	Mean Diff.	95.00% CI	Below thre	Summary	Adjusted P Value
CHOW vs. DSS-PN	0.09291	-0.4553 to	No	ns	0.9568
CHOW vs. TNFR^{KO} CH	0.278	-0.2702 to	No	*	0.0135
CHOW vs. TNFR^{KO} DS	0.187	-0.3611 to	No	ns	0.7452
DSS-PN vs. TNFR^{KO} C	0.1851	-0.3631 to	No	ns	0.751
DSS-PN vs. TNFR^{KO} D	0.09413	-0.4540 to	No	ns	0.9552
TNFR^{KO} CHOW vs. TN	-0.09095	-0.6391 to	No	ns	0.9593

Dbp

Tukey's multiple compa	Mean Diff.	95.00% CI	Below thre	Summary	Adjusted P Value
Chow vs. DSS-PN	-3.201	-21.37 to 1	No	**	0.0097
Chow vs. IL1^{KO} Chow	-0.01871	-19.65 to 1	No	ns	>0.9999

Chow vs. IL1^{KO} DSS-PN	-30.47	-48.64 to -1	Yes	**	0.0018
DSS-PN vs. IL1^{KO} Chow	3.182	-16.45 to 2	No	ns	0.9602
DSS-PN vs. IL1^{KO} DSS-	-27.26	-45.44 to -9	Yes	**	0.0041
IL1^{KO} Chow vs. IL1^{KO} DS	-30.45	-50.08 to -1	Yes	**	0.0033

Tukey's multiple compa	Mean Diff.	95.00% CI	Below thres	Summary	Adjusted P Value
CHOW vs. DSS-PN	-14.14	-22.07 to -6	Yes	**	0.0011
CHOW vs. TNFR^{KO} CH	-0.4963	-8.434 to 7	No	ns	0.9975
CHOW vs. TNFR^{KO} DS	-0.3007	-8.874 to 8	No	ns	0.9996
DSS-PN vs. TNFR^{KO} CH	13.64	5.702 to 21	Yes	**	0.0015
DSS-PN vs. TNFR^{KO} DS	13.83	5.262 to 22	Yes	**	0.0024
TNFR^{KO} CHOW vs. TN	0.1956	-8.378 to 8	No	ns	0.9999

Per1

Tukey's multiple compa	Mean Diff.	95.00% CI	Below thres	Summary	Adjusted P Value
Chow vs. DSS-PN	0.3895	-0.6159 to	No	ns	0.659
Chow vs. IL1^{KO} Chow	1.02	-0.06582 to	No	ns	0.0676
Chow vs. IL1^{KO} DSS-PN	-2.06	-3.066 to -1	Yes	***	0.0003
DSS-PN vs. IL1^{KO} Chow	0.6306	-0.4553 to	No	ns	0.3467
DSS-PN vs. IL1^{KO} DSS-	-2.45	-3.455 to -1	Yes	****	<0.0001
IL1^{KO} Chow vs. IL1^{KO} DS	-3.081	-4.167 to -1	Yes	****	<0.0001

Tukey's multiple compa	Mean Diff.	95.00% CI	Below thres	Summary	Adjusted P Value
CHOW vs. DSS-PN	0.2757	-0.01538 to	No	ns	0.0651
CHOW vs. TNFR^{KO} CH	0.6337	0.3426 to 0	Yes	***	0.0002
CHOW vs. TNFR^{KO} DS	0.803	0.4886 to 1	Yes	****	<0.0001
DSS-PN vs. TNFR^{KO} CH	0.358	0.06687 to	Yes	*	0.0157
DSS-PN vs. TNFR^{KO} DS	0.5273	0.2129 to 0	Yes	**	0.0018
TNFR^{KO} CHOW vs. TN	0.1693	-0.1451 to	No	ns	0.4069

Per2

Tukey's multiple compa	Mean Diff.	95.00% CI	Below thres	Summary	Adjusted P Value
Chow vs. DSS-PN	-3.197	-8.529 to 2	No	ns	0.3216
Chow vs. IL1^{KO} Chow	-2.633	-8.392 to 3	No	**	0.0043
Chow vs. IL1^{KO} DSS-PN	-11.76	-17.09 to -6	Yes	***	0.0002
DSS-PN vs. IL1^{KO} Chow	0.5643	-5.195 to 6	No	ns	0.9906
DSS-PN vs. IL1^{KO} DSS-	-8.558	-13.89 to -3	Yes	**	0.0025
IL1^{KO} Chow vs. IL1^{KO} DS	-9.122	-14.88 to -3	Yes	**	0.0028

Tukey's multiple compa	Mean Diff.	95.00% CI	Below thres	Summary	Adjusted P Value
CHOW vs. DSS-PN	-2.246	-4.239 to -0	Yes	*	0.0259
CHOW vs. TNFR^{KO} CH	-0.7156	-2.708 to 1	No	ns	0.7156
CHOW vs. TNFR^{KO} DS	-1.43	-3.423 to 0	No	ns	0.1987

DSS-PN vs. TNFR^{KO} Cl	1.531	-0.4621 to	No	ns	0.1573
DSS-PN vs. TNFR^{KO} D!	0.8166	-1.176 to 2	No	ns	0.6287
TNFR^{KO} CHOW vs. TN	-0.7142	-2.707 to 1	No	ns	0.7167

Dec1

Tukey's multiple compa	Mean Diff.	95.00% CI	Below thre:	Summary	Adjusted P Value
CHOW vs. DSS-PN	-2.246	-4.239 to -(	Yes	*	0.0259
CHOW vs. TNFR^{KO} CH	-0.7156	-2.708 to 1	No	ns	0.7156
CHOW vs. TNFR^{KO} DS	-1.43	-3.423 to 0	No	ns	0.1987
DSS-PN vs. TNFR^{KO} Cl	1.531	-0.4621 to	No	ns	0.1573
DSS-PN vs. TNFR^{KO} D!	0.8166	-1.176 to 2	No	ns	0.6287
TNFR^{KO} CHOW vs. TN	-0.7142	-2.707 to 1	No	ns	0.7167

Tukey's multiple compa	Mean Diff.	95.00% CI	Below thre:	Summary	Adjusted P Value
CHOW vs. DSS-PN	0.7247	-0.09507 to	No	ns	0.0898
CHOW vs. TNFR^{KO} CH	0.6572	-0.1626 to	No	ns	0.1343
CHOW vs. TNFR^{KO} DS	0.5385	-0.2813 to	No	ns	0.2593
DSS-PN vs. TNFR^{KO} Cl	-0.06755	-0.8874 to	No	ns	0.9946
DSS-PN vs. TNFR^{KO} D!	-0.1863	-1.006 to 0	No	ns	0.9047
TNFR^{KO} CHOW vs. TN	-0.1187	-0.9385 to	No	ns	0.9722

Dec2

Tukey's multiple compa	Mean Diff.	95.00% CI	Below thre:	Summary	Adjusted P Value
Chow vs. DSS-PN	-74.48	-180.7 to 3	No	ns	0.2091
Chow vs. IL1^{KO} Chow	-50.16	-164.9 to 6	No	ns	0.5724
Chow vs. IL1^{KO} DSS-PN	-142.4	-248.6 to -:	Yes	**	0.0091
DSS-PN vs. IL1^{KO} Chow	24.32	-90.40 to 1	No	ns	0.9175
DSS-PN vs. IL1^{KO} DSS-	-67.87	-174.1 to 3	No	ns	0.2736
IL1^{KO} Chow vs. IL1^{KO} D!	-92.2	-206.9 to 2	No	ns	0.1307

Sample N	Target Na	RQ							
313r1	Clock	0.225181	2.0%DSS 4D/TPN 14D	CHOW	DSS	PN	DSS-PN	TNF CHO	TNF DSS-PN
313r2	Clock	0.22745		0.865302	0.651567	1.083039	0.232062	0.530537	1.131399
313r3	Clock	0.243556							
317r1	Clock	0.619034	2.0%DSS 4D/CHOW 14D						
317r2	Clock	0.661737		0.982079	1.079945	1.506914	1.101462	0.452393	0.452135
317r3	Clock	0.673931							
321r1	Clock	0.991966	2.0%DSS 4D/CHOW 14D						
321r2	Clock	1.111063		0.796676	0.609665	1.101068	0.701055	0.600377	0.499601
321r3	Clock	1.136805							
339r1	Clock	1.005267	TPN 14D						
339r2	Clock	1.118686		0.631141	0.498395	0.781504	0.868977	0.579935	0.443911
339r3	Clock	1.125166							
343r1	Clock	1.280439	TPN 14D						
343r2	Clock	1.601457							
343r3	Clock	1.638847							
344r1	Clock	1.047792	TPN 14D						
344r2	Clock	1.09317							
344r3	Clock	1.162242							
345r1	Clock	0.725717	TPN 14D						
345r2	Clock	0.822957							
345r3	Clock	0.795838							
568r1	Clock	0.577327	2.0%DSS 4D/CHOW 14D						
568r2	Clock	0.609803							
568r3	Clock	0.641864							
571r1	Clock	0.470519	2.0%DSS 4D/CHOW 14D						
571r2	Clock	0.526271							
571r3	Clock	0.470673							
587r1	Clock	1.084491	TNF(-/-) 2.0%DSS(4D)/TPN 14D						
587r2	Clock	1.065795							
587r3	Clock	1.243913							
604r1	Clock	0.426119	TNF(-/-) 2.0%DSS(4D)/TPN 14D						
604r2	Clock	0.482352							
604r3	Clock	0.447936							
605r1	Clock	0.502087	TNF(-/-) 2.0%DSS(4D)/TPN 14D						
605r2	Clock	0.561224							
605r3	Clock	0.435491							
606r1	Clock	0.44476	TNF(-/-) 2.0%DSS(4D)/TPN 14D						
606r2	Clock	0.411349							
606r3	Clock	0.475622							
616r1	Clock	0.534992	TNF(-/-)/CHOW 14D						
616r2	Clock	0.470355							
616r3	Clock	0.586265							
618r1	Clock	0.467166	TNF(-/-)/CHOW 14D						
618r2	Clock	0.474556							
618r3	Clock	0.415458							
619r1	Clock	0.566599	TNF(-/-)/CHOW 14D						
619r2	Clock	0.63814							
619r3	Clock	0.596393							
620r1	Clock	0.550071	TNF(-/-)/CHOW 14D						
620r2	Clock	0.589269							
620r3	Clock	0.600466							
688r1	Clock	1.064348	2.0%DSS 4D/TPN 14D						
688r2	Clock	1.014196							
688r3	Clock	1.225842							
689r1	Clock	0.642611	2.0%DSS 4D/TPN 14D						
689r2	Clock	0.699369							
689r3	Clock	0.761185							
690r1	Clock	0.793186	2.0%DSS 4D/TPN 14D						
690r2	Clock	0.885181							
690r3	Clock	0.928564							
847r1	Clock	0.747869	CHOW						
847r2	Clock	0.923268							
847r3	Clock	0.924769							
848r1	Clock	1	CHOW						
848r2	Clock	1.016348							
848r3	Clock	0.929888							
849r1	Clock	0.72524	CHOW						
849r2	Clock	0.759169							
849r3	Clock	0.905617							
850r1	Clock	0.650109	CHOW						
850r2	Clock	0.61634							
850r3	Clock	0.626974							

Sample N	Target Na	RQ							
313r1	Per1	0.693221	2.0%DSS 4D/TPN 14D	CHOW	DSS	PN	DSS-PN	TNF CHO	TNF DSS-PN
313r2	Per1	0.489843		0.768656	0.073876	0.955605	0.598054	0.310038	1.374772
313r3	Per1	0.611099							
317r1	Per1	0.06294	2.0%DSS 4D/CHOW 14D						
317r2	Per1	0.079351		1.131529	0.128587	1.093755	0.567933	0.197566	0.031607
317r3	Per1	0.079337							
321r1	Per1	0.191315	2.0%DSS 4D/CHOW 14D						
321r2	Per1	0.155833		0.932145	0.178254	0.054137	0.765284	0.177267	0.116632
321r3	Per1	0.038615							
339r1	Per1	0.910558	TPN 14D						
339r2	Per1	1.002609		0.63826	0.054982	0.219283	0.43645	0.250973	0.045633
339r3	Per1	0.953648							
343r1	Per1	1.27867	TPN 14D						
343r2	Per1	0.968487							
343r3	Per1	1.034107							
344r1	Per1	0.095166	TPN 14D						
344r2	Per1	0.03315							
344r3	Per1	0.034095							
345r1	Per1	0.176803	TPN 14D						
345r2	Per1	0.256168							
345r3	Per1	0.224878							
568r1	Per1	0.126617	2.0%DSS 4D/CHOW 14D						
568r2	Per1	0.158343							
568r3	Per1	0.249803							
571r1	Per1	0.036897	2.0%DSS 4D/CHOW 14D						
571r2	Per1	0.073067							
571r3	Per1	0.091962							
587r1	Per1	1.151578	TNF(-/-) 2.0%DSS(4D)/TPN 14D						
587r2	Per1	1.434067							
587r3	Per1	1.538672							
604r1	Per1	0.047976	TNF(-/-) 2.0%DSS(4D)/TPN 14D						
604r2	Per1	0.026333							
604r3	Per1	0.020514							
605r1	Per1	0.086483	TNF(-/-) 2.0%DSS(4D)/TPN 14D						
605r2	Per1	0.154188							
605r3	Per1	0.109226							
606r1	Per1	0.023806	TNF(-/-) 2.0%DSS(4D)/TPN 14D						
606r2	Per1	0.042411							
606r3	Per1	0.070682							
616r1	Per1	0.250989	TNF(-/-)/CHOW 14D						
616r2	Per1	0.284865							
616r3	Per1	0.39426							
618r1	Per1	0.19415	TNF(-/-)/CHOW 14D						
618r2	Per1	0.182538							
618r3	Per1	0.21601							
619r1	Per1	0.182437	TNF(-/-)/CHOW 14D						
619r2	Per1	0.210823							
619r3	Per1	0.138541							
620r1	Per1	0.224689	TNF(-/-)/CHOW 14D						
620r2	Per1	0.28056							
620r3	Per1	0.247669							
688r1	Per1	0.485099	2.0%DSS 4D/TPN 14D						
688r2	Per1	0.428835							
688r3	Per1	0.789864							
689r1	Per1	0.723229	2.0%DSS 4D/TPN 14D						
689r2	Per1	0.794225							
689r3	Per1	0.7784							
690r1	Per1	0.375595	2.0%DSS 4D/TPN 14D						
690r2	Per1	0.421883							
690r3	Per1	0.511872							
847r1	Per1	0.70138	CHOW						
847r2	Per1	0.816784							
847r3	Per1	0.787805							
848r1	Per1	1	CHOW						
848r2	Per1	1.178218							
848r3	Per1	1.216369							
849r1	Per1	0.881701	CHOW						
849r2	Per1	0.874798							
849r3	Per1	1.039935							
850r1	Per1	0.530192	CHOW						
850r2	Per1	0.625728							
850r3	Per1	0.758858							

Sample N	Target Na	RQ						
314r1	Dec1	0.557969	2.0%DSS 4D/TPN 14D	Dec1	Chow	DSS-PN	TNFRKO	TNFRKO DSS-PN
314r2	Dec1	0.446277			0.459123	0.488325	0.594636	1.338657
314r3	Dec1	0.460729						
317r1	Dec1	0.615722	2.0%DSS 4D/CHOW 14D					
317r2	Dec1	0.85005			1.030582	1.390671	0.541527	0.340254
317r3	Dec1	0.731632						
321r1	Dec1	1.247957	2.0%DSS 4D/CHOW 14D					
321r2	Dec1	1.487046			1.674076	0.623709	0.702732	0.738623
321r3	Dec1	1.426207						
339r1	Dec1	#VALUE!	TPN 14D					
339r2	Dec1	#VALUE!			1.792307	0.513929	0.488411	0.384684
339r3	Dec1	#VALUE!						
343r1	Dec1	#VALUE!	TPN 14D					
343r2	Dec1	#VALUE!				0.431162		
343r3	Dec1	#VALUE!						
344r1	Dec1	#VALUE!	TPN 14D					
344r2	Dec1	#VALUE!						
344r3	Dec1	#VALUE!						
345r1	Dec1	#VALUE!	TPN 14D					
345r2	Dec1	#VALUE!						
345r3	Dec1	#VALUE!						
545r1	Dec1	0.38379	2.0%DSS 4D/TPN 14D					
545r2	Dec1	0.392563						
545r3	Dec1	0.517134						
547r1	Dec1	#VALUE!						
547r2	Dec1	#VALUE!						
547r3	Dec1	#VALUE!						
568r1	Dec1	0.284465	2.0%DSS 4D/CHOW 14D					
568r2	Dec1	0.332764						
568r3	Dec1	0.329119						
571r1	Dec1	0.176097	2.0%DSS 4D/CHOW 14D					
571r2	Dec1	0.161061						
571r3	Dec1	0.132021						
587r1	Dec1	1.292919	TNF(-/-) 2.0%DSS(4D)/TPN 14D					
587r2	Dec1	1.131663						
587r3	Dec1	1.591388						
604r1	Dec1	0.293522	TNF(-/-) 2.0%DSS(4D)/TPN 14D					
604r2	Dec1	0.318136						
604r3	Dec1	0.409105						
605r1	Dec1	0.7027	TNF(-/-) 2.0%DSS(4D)/TPN 14D					
605r2	Dec1	0.822963						
605r3	Dec1	0.690208						
606r1	Dec1	0.410186	TNF(-/-) 2.0%DSS(4D)/TPN 14D					
606r2	Dec1	0.38738						
606r3	Dec1	0.356487						
616r1	Dec1	0.559421	TNF(-/-)/CHOW 14D					
616r2	Dec1	0.670922						
616r3	Dec1	0.553564						
618r1	Dec1	0.438462	TNF(-/-)/CHOW 14D					
618r2	Dec1	0.58627						
618r3	Dec1	0.59985						
619r1	Dec1	0.689373	TNF(-/-)/CHOW 14D					
619r2	Dec1	0.703757						
619r3	Dec1	0.715065						
620r1	Dec1	0.463673	TNF(-/-)/CHOW 14D					
620r2	Dec1	0.494586						
620r3	Dec1	0.506975						
686r1	Dec1	#VALUE!						
686r2	Dec1	#VALUE!						
686r3	Dec1	#VALUE!						
688r1	Dec1	1.069766	2.0%DSS 4D/TPN 14D					
688r2	Dec1	1.455562						
688r3	Dec1	1.646684						
689r1	Dec1	0.64556	2.0%DSS 4D/TPN 14D					
689r2	Dec1	0.617935						
689r3	Dec1	0.607632						
690r1	Dec1	0.482	2.0%DSS 4D/TPN 14D					
690r2	Dec1	0.522219						
690r3	Dec1	0.537567						
847r1	Dec1	0.428712	CHOW					
847r2	Dec1	0.458526						
847r3	Dec1	0.49013						
848r1	Dec1	1	CHOW					
848r2	Dec1	1.083468						
848r3	Dec1	1.008277						
849r1	Dec1	1.5008	CHOW					
849r2	Dec1	1.680645						
849r3	Dec1	1.840783						
850r1	Dec1	1.687581	CHOW					
850r2	Dec1	1.668471						
850r3	Dec1	2.020869						

Sample N	Target Na	RQ						
314r1	Dec2	#VALUE!	2.0%DSS	Dec2	Chow	DSS-PN	TNFRKO	TNFRKO DSS-PN
314r2	Dec2	#VALUE!			0.769141	24.34463	29.40974	532.9739
314r3	Dec2	#VALUE!						
317r1	Dec2	27.41462	2.0%DSS 4D/CHOW 14D					
317r2	Dec2	21.62215			1.80477	70.23922	41.88387	88.16236
317r3	Dec2	25.18012						
321r1	Dec2	214.7589	2.0%DSS 4D/CHOW 14D					
321r2	Dec2	214.4198			3.581337	321.3772	37.43031	116.6535
321r3	Dec2	250.0825						
339r1	Dec2	#VALUE!	TPN 14D					
339r2	Dec2	#VALUE!			3.355206	196.7116	20.20075	31.74342
339r3	Dec2	#VALUE!						
343r1	Dec2	#VALUE!	TPN 14D					
343r2	Dec2	#VALUE!						
343r3	Dec2	#VALUE!						
344r1	Dec2	#VALUE!	TPN 14D					
344r2	Dec2	#VALUE!						
344r3	Dec2	#VALUE!						
345r1	Dec2	#VALUE!	TPN 14D					
345r2	Dec2	#VALUE!						
345r3	Dec2	#VALUE!						
545r1	Dec2	18.40298	2.0%DSS 4D/TPN 14D					
545r2	Dec2	32.35339						
545r3	Dec2	22.27753						
547r1	Dec2	94.42216						
547r2	Dec2	70.79595						
547r3	Dec2	101.8704						
568r1	Dec2	22.76293	2.0%DSS 4D/CHOW 14D					
568r2	Dec2	29.5916						
568r3	Dec2	33.84564						
571r1	Dec2	37.05725	2.0%DSS 4D/CHOW 14D					
571r2	Dec2	25.32567						
571r3	Dec2	32.46539						
587r1	Dec2	455.6562	TNF(-/-) 2.0%DSS(4D)/TPN 14D					
587r2	Dec2	492.7386						
587r3	Dec2	650.5269						
604r1	Dec2	57.05731	TNF(-/-) 2.0%DSS(4D)/TPN 14D					
604r2	Dec2	83.87851						
604r3	Dec2	123.5513						
605r1	Dec2	99.03224	TNF(-/-) 2.0%DSS(4D)/TPN 14D					
605r2	Dec2	92.36226						
605r3	Dec2	158.566						
606r1	Dec2	34.31271	TNF(-/-) 2.0%DSS(4D)/TPN 14D					
606r2	Dec2	30.77218						
606r3	Dec2	30.14537						
616r1	Dec2	21.79568	TNF(-/-)/CHOW 14D					
616r2	Dec2	28.45838						
616r3	Dec2	37.97516						
618r1	Dec2	41.44094	TNF(-/-)/CHOW 14D					
618r2	Dec2	39.56658						
618r3	Dec2	44.64409						
619r1	Dec2	25.31659	TNF(-/-)/CHOW 14D					
619r2	Dec2	23.7107						
619r3	Dec2	63.26363						
620r1	Dec2	13.4602	TNF(-/-)/CHOW 14D					
620r2	Dec2	21.1064						
620r3	Dec2	26.03565						
686r1	Dec2	#VALUE!						
686r2	Dec2	#VALUE!						
686r3	Dec2	#VALUE!						
688r1	Dec2	44.31654	2.0%DSS 4D/TPN 14D					
688r2	Dec2	96.00619						
688r3	Dec2	70.39492						
689r1	Dec2	324.3525	2.0%DSS 4D/TPN 14D					
689r2	Dec2	323.7805						
689r3	Dec2	315.9987						
690r1	Dec2	179.7317	2.0%DSS 4D/TPN 14D					
690r2	Dec2	197.7122						
690r3	Dec2	212.6909						
847r1	Dec2	0.766277	CHOW					
847r2	Dec2	0.995528						
847r3	Dec2	0.545618						
848r1	Dec2	1	CHOW					
848r2	Dec2	2.429129						
848r3	Dec2	1.985181						
849r1	Dec2	2.512881	CHOW					
849r2	Dec2	4.574649						
849r3	Dec2	3.656482						
850r1	Dec2	1.973593	CHOW					
850r2	Dec2	4.294064						
850r3	Dec2	3.79796						

Sample N	Target Na	RQ						
314r1	Npas2	#VALUE!	2.0%DSS	Npas2	Chow	DSS-PN	TNFRKO	TNFRKO DSS-PN
314r2	Npas2	#VALUE!			0.587695	0.867812155	0.912965	0.669269
314r3	Npas2	#VALUE!						
317r1	Npas2	0.234634	2.0%DSS 4D/CHOW 14D					
317r2	Npas2	0.18325			1.045107	0.635032213	0.5727	0.707872
317r3	Npas2	0.435017						
321r1	Npas2	0.279782	2.0%DSS 4D/CHOW 14D					
321r2	Npas2	0.423266			0.732999	0.252897107	0.716248	0.439425
321r3	Npas2	0.450581						
339r1	Npas2	0.305066	TPN 14D					
339r2	Npas2	0.53025			0.41795	0.276087436	0.433551	0.582416
339r3	Npas2	0.510848						
343r1	Npas2	1.374154	TPN 14D					
343r2	Npas2	1.363208						
343r3	Npas2	1.60492						
344r1	Npas2	0.722634	TPN 14D					
344r2	Npas2	0.729936						
344r3	Npas2	0.933912						
345r1	Npas2	0.460459	TPN 14D					
345r2	Npas2	0.628113						
345r3	Npas2	0.413986						
545r1	Npas2	0.638039	2.0%DSS 4D/TPN 14D					
545r2	Npas2	0.721842						
545r3	Npas2	0.545215						
547r1	Npas2	#VALUE!						
547r2	Npas2	#VALUE!						
547r3	Npas2	#VALUE!						
568r1	Npas2	0.487462	2.0%DSS 4D/CHOW 14D					
568r2	Npas2	0.711244						
568r3	Npas2	0.569589						
571r1	Npas2	0.395255	2.0%DSS 4D/CHOW 14D					
571r2	Npas2	0.309802						
571r3	Npas2	0.390815						
587r1	Npas2	0.448292	TNF(-/-) 2.0%DSS(4D)/TPN 14D					
587r2	Npas2	0.951091						
587r3	Npas2	0.608425						
604r1	Npas2	0.556172	TNF(-/-) 2.0%DSS(4D)/TPN 14D					
604r2	Npas2	0.6962						
604r3	Npas2	0.871244						
605r1	Npas2	0.347543	TNF(-/-) 2.0%DSS(4D)/TPN 14D					
605r2	Npas2	0.474827						
605r3	Npas2	0.495906						
606r1	Npas2	0.4185	TNF(-/-) 2.0%DSS(4D)/TPN 14D					
606r2	Npas2	0.632878						
606r3	Npas2	0.695869						
616r1	Npas2	0.950501	TNF(-/-)/CHOW 14D					
616r2	Npas2	0.903727						
616r3	Npas2	0.884668						
618r1	Npas2	0.459595	TNF(-/-)/CHOW 14D					
618r2	Npas2	0.605251						
618r3	Npas2	0.653254						
619r1	Npas2	0.641454	TNF(-/-)/CHOW 14D					
619r2	Npas2	0.736028						
619r3	Npas2	0.771262						
620r1	Npas2	0.46367	TNF(-/-)/CHOW 14D					
620r2	Npas2	0.384666						
620r3	Npas2	0.452318						
686r1	Npas2	0.873213	2.0%DSS 4D/TPN 14D					
686r2	Npas2	0.813384						
686r3	Npas2	0.91684						
688r1	Npas2	#VALUE!	2.0%DSS 4D/TPN 14D					
688r2	Npas2	#VALUE!						
688r3	Npas2	#VALUE!						
689r1	Npas2	0.169636	2.0%DSS 4D/TPN 14D					
689r2	Npas2	0.222759						
689r3	Npas2	0.366297						
690r1	Npas2	0.284611	2.0%DSS 4D/TPN 14D					
690r2	Npas2	0.217463						
690r3	Npas2	0.326189						
847r1	Npas2	0.561546	CHOW					
847r2	Npas2	0.56893						
847r3	Npas2	0.632608						
848r1	Npas2	1	CHOW					
848r2	Npas2	0.992775						
848r3	Npas2	1.142545						
849r1	Npas2	0.725558	CHOW					
849r2	Npas2	0.774227						
849r3	Npas2	0.699213						
850r1	Npas2	0.388114	CHOW					
850r2	Npas2	0.425709						
850r3	Npas2	0.440027						

Sample N	Target Na	RQ								
313r1	Cry1	0.129413	2.0%DSS	Cry1	Chow	DSS-PN	IL1KO Ch	IL1KO DS	TNFRKO	TNFRKO DSS-PN
313r2	Cry1	0.140293			0.576317653	0.130756	1.64741	0.611749	1.167032	0.362351
313r3	Cry1	0.122563								
349r1	Cry1	0.71929	(IL1-/-) 2.0%DSS/TPN14D							
349r2	Cry1	0.52212			1.102197468	0.429307	1.729971	0.298474	0.758644	0.295147
349r3	Cry1	0.593838								
351r1	Cry1	0.36548	(IL1-/-) 2.0%DSS/TPN14D							
351r2	Cry1	0.248035			0.824543564	0.315321	1.038645	0.685113	0.7973	0.623702
351r3	Cry1	0.281908								
352r1	Cry1	0.521591	(IL1-/-) 2.0%DSS/TPN14D							
352r2	Cry1	0.671461			0.679233659	0.471318		0.409189	0.430835	0.579547
352r3	Cry1	0.862287								
353r1	Cry1	0.396953	(IL1-/-) 2.0%DSS/TPN14D							
353r2	Cry1	0.421088								
353r3	Cry1	0.409526		Cry1	Chow	DSS-PN	TNFRKO	TNFRKO DSS-PN		
364r1	Cry1	1.626414	(IL1-/-) CHOW 14D		0.576317653	0.130756	1.167032	0.362351		
364r2	Cry1	1.911567								
364r3	Cry1	1.404249								
365r1	Cry1	1.712678	(IL1-/-) CHOW 14D		1.102197468	0.429307	0.758644	0.295147		
365r2	Cry1	1.597047								
365r3	Cry1	1.880187								
587r1	Cry1	0.348978	TNF(-/-) 2.0%DSS(4I		0.824543564	0.315321	0.7973	0.623702		
587r2	Cry1	0.271306								
587r3	Cry1	0.46677								
604r1	Cry1	0.328845	TNF(-/-) 2.0%DSS(4I		0.679233659	0.471318	0.430835	0.579547		
604r2	Cry1	0.302302								
604r3	Cry1	0.254294								
605r1	Cry1	0.649368	TNF(-/-) 2.0%DSS(4D)/TPN 14D							
605r2	Cry1	0.631976								
605r3	Cry1	0.589762								
606r1	Cry1	0.578056	TNF(-/-) 2.0%DSS(4D)/TPN 14D							
606r2	Cry1	0.579197								
606r3	Cry1	0.581387								
616r1	Cry1	1.167972	TNF(-/-)/CHOW 14D							
616r2	Cry1	1.167096								
616r3	Cry1	1.166027								
618r1	Cry1	0.850011	TNF(-/-)/CHOW 14D							
618r2	Cry1	0.611728								
618r3	Cry1	0.814195								
619r1	Cry1	0.794487	TNF(-/-)/CHOW 14D							
619r2	Cry1	0.855346								
619r3	Cry1	0.742068								
620r1	Cry1	0.398961	TNF(-/-)/CHOW 14D							
620r2	Cry1	0.467473								
620r3	Cry1	0.426071								
665r1	Cry1	1.031525	(IL1-/-) CHOW 14D							
665r2	Cry1	1.081817								
665r3	Cry1	1.002594								
688r1	Cry1	0.421422	2.0%DSS 4D/TPN 14D							
688r2	Cry1	0.613329								
688r3	Cry1	0.379203								
689r1	Cry1	0.441626	2.0%DSS 4D/TPN 14D							
689r2	Cry1	0.472399								
689r3	Cry1	0.373896								
690r1	Cry1	0.284062	2.0%DSS 4D/TPN 14D							
690r2	Cry1	0.317342								
690r3	Cry1	0.344557								
847r1	Cry1	0.644962	CHOW							
847r2	Cry1	0.497409								
847r3	Cry1	0.586582								
848r1	Cry1	1	CHOW							
848r2	Cry1	1.075321								
848r3	Cry1	1.231272								
849r1	Cry1	0.785341	CHOW							
849r2	Cry1	0.820752								
849r3	Cry1	0.867538								
850r1	Cry1	0.65074	CHOW							
850r2	Cry1	0.667415								
850r3	Cry1	0.719546								

Sample N	Target Na	RQ								
313r1	Cry2	0.587364	2.0%DSS 4D/TPN 1	Cry2	Chow	DSS-PN	IL1KO Ch	IL1KO DS	TNFRKO	TNFRKO DSS-PN
313r2	Cry2	0.820008			0.637124	0.792824	1.984453	2.693141	0.62542	0.900402
313r3	Cry2	0.971099								
349r1	Cry2	1.656596	(IL1-/-) 2.0%DSS/TPN14D							
349r2	Cry2	4.009117			1.081262	0.777371	1.500794	0.716079	0.662262	0.173559
349r3	Cry2	2.41371								
351r1	Cry2	0.750891	(IL1-/-) 2.0%DSS/TPN14D							
351r2	Cry2	0.730379			1.283059	0.60282	0.765744	2.503196	0.440274	0.456865
351r3	Cry2	0.666967								
352r1	Cry2	1.791668	(IL1-/-) 2.0%DSS/TPN14D							
352r2	Cry2	2.457645			0.784425	0.61205		1.525419	0.515831	0.368171
352r3	Cry2	3.260276								
353r1	Cry2	1.426744	(IL1-/-) 2.0%DSS/TPN14D							
353r2	Cry2	1.520228		Cry2	Chow	DSS-PN	TNFRKO	TNFRKO DSS-PN		
353r3	Cry2	1.629285			0.637124	0.792824	0.62542	0.900402		
364r1	Cry2	1.705987	(IL1-/-) CHOW 14D							
364r2	Cry2	2.102436								
364r3	Cry2	2.144935			1.081262	0.777371	0.662262	0.173559		
365r1	Cry2	1.244451	(IL1-/-) CHOW 14D							
365r2	Cry2	1.40578								
365r3	Cry2	1.852152			1.283059	0.60282	0.440274	0.456865		
587r1	Cry2	1.069394	TNF(-/-) 2.0%DSS(4D)/TPN 14D							
587r2	Cry2	1.527224								
587r3	Cry2	0.104588			0.784425	0.61205	0.515831	0.368171		
604r1	Cry2	0.164428	TNF(-/-) 2.0%DSS(4D)/TPN 14D							
604r2	Cry2	0.120013								
604r3	Cry2	0.236238								
605r1	Cry2	0.412557	TNF(-/-) 2.0%DSS(4D)/TPN 14D							
605r2	Cry2	0.344225								
605r3	Cry2	0.613814								
606r1	Cry2	0.468634	TNF(-/-) 2.0%DSS(4D)/TPN 14D							
606r2	Cry2	0.341225								
606r3	Cry2	0.294654								
616r1	Cry2	0.551686	TNF(-/-)/CHOW 14D							
616r2	Cry2	0.713887								
616r3	Cry2	0.610688								
618r1	Cry2	0.481161	TNF(-/-)/CHOW 14D							
618r2	Cry2	0.689507								
618r3	Cry2	0.816118								
619r1	Cry2	0.520643	TNF(-/-)/CHOW 14D							
619r2	Cry2	0.380262								
619r3	Cry2	0.419919								
620r1	Cry2	0.419228	TNF(-/-)/CHOW 14D							
620r2	Cry2	0.522405								
620r3	Cry2	0.60586								
665r1	Cry2	0.7733	(IL1-/-) CHOW 14D							
665r2	Cry2	0.786388								
665r3	Cry2	0.737545								
688r1	Cry2	0.527401	2.0%DSS 4D/TPN 14D							
688r2	Cry2	0.625158								
688r3	Cry2	0.68359								
689r1	Cry2	0.776272	2.0%DSS 4D/TPN 14D							
689r2	Cry2	0.851683								
689r3	Cry2	0.704159								
690r1	Cry2	0.491793	2.0%DSS 4D/TPN 14D							
690r2	Cry2	0.498313								
690r3	Cry2	0.818353								
847r1	Cry2	0.607132	CHOW							
847r2	Cry2	0.560385								
847r3	Cry2	0.743856								
848r1	Cry2	1	CHOW							
848r2	Cry2	1.028421								
848r3	Cry2	1.215364								
849r1	Cry2	1.294848	CHOW							
849r2	Cry2	1.325136								
849r3	Cry2	1.229192								
850r1	Cry2	0.729858	CHOW							
850r2	Cry2	0.834057								
850r3	Cry2	0.789361								

Sample N	Target Na	RQ						
313r1	Nfil3	0.749414	2.0%DSS 4D/TPN 1	Nfil3	Chow	DSS-PN	TNFRKO	TNFRKO DSS-PN
313r2	Nfil3	0.936243			0.895549	0.870378	1.222214	10.66091
313r3	Nfil3	0.925479						
349r1	Nfil3	3.448293	(IL1-/-) 2.0%DSS/TPN14D					
349r2	Nfil3	5.120347			1.179318	9.100446	1.539075	1.749933
349r3	Nfil3	4.525426						
351r1	Nfil3	2.191984	(IL1-/-) 2.0%DSS/TPN14D					
351r2	Nfil3	2.43585			1.106297	4.615524	1.275573	3.620066
351r3	Nfil3	2.750388						
352r1	Nfil3	2.395014	(IL1-/-) 2.0%DSS/TPN14D					
352r2	Nfil3	2.307993			1.84045	2.612142	1.028271	0.951887
352r3	Nfil3	2.706853						
353r1	Nfil3	1.718484	(IL1-/-) 2.0%DSS/TPN14D					
353r2	Nfil3	1.727366			Chow	DSS-PN	IL1KO Ch	IL1KO DSS-PN
353r3	Nfil3	1.711233			0.895549	0.870378	3.418928	4.364689
364r1	Nfil3	3.226766	(IL1-/-) CHOW 14D					
364r2	Nfil3	3.541029						
364r3	Nfil3	3.488988			1.179318	9.100446	1.432586	2.459407
365r1	Nfil3	1.401226	(IL1-/-) CHOW 14D					
365r2	Nfil3	1.276512						
365r3	Nfil3	1.62002			1.106297	4.615524	1.058578	2.469953
587r1	Nfil3	10.39248	TNF(-/-) 2.0%DSS(4D)/TPN 14D					
587r2	Nfil3	10.87989						
587r3	Nfil3	10.71036			1.84045	2.612142		1.719028
604r1	Nfil3	1.709054	TNF(-/-) 2.0%DSS(4D)/TPN 14D					
604r2	Nfil3	1.79693						
604r3	Nfil3	1.743816						
605r1	Nfil3	3.573921	TNF(-/-) 2.0%DSS(4D)/TPN 14D					
605r2	Nfil3	3.451226						
605r3	Nfil3	3.835051						
606r1	Nfil3	0.974271	TNF(-/-) 2.0%DSS(4D)/TPN 14D					
606r2	Nfil3	0.888947						
606r3	Nfil3	0.992444						
616r1	Nfil3	1.230905	TNF(-/-)/CHOW 14D					
616r2	Nfil3	1.13496						
616r3	Nfil3	1.300776						
618r1	Nfil3	1.556445	TNF(-/-)/CHOW 14D					
618r2	Nfil3	1.412803						
618r3	Nfil3	1.647977						
619r1	Nfil3	1.251983	TNF(-/-)/CHOW 14D					
619r2	Nfil3	1.341673						
619r3	Nfil3	1.233063						
620r1	Nfil3	0.995175	TNF(-/-)/CHOW 14D					
620r2	Nfil3	1.054757						
620r3	Nfil3	1.03488						
665r1	Nfil3	1.014482	(IL1-/-) CHOW 14D					
665r2	Nfil3	1.105088						
665r3	Nfil3	1.056164						
688r1	Nfil3	2.272642	2.0%DSS 4D/TPN 14D					
688r2	Nfil3	2.754035						
688r3	Nfil3	2.809749						
689r1	Nfil3	8.659422	2.0%DSS 4D/TPN 14D					
689r2	Nfil3	9.24199						
689r3	Nfil3	9.399926						
690r1	Nfil3	4.147499	2.0%DSS 4D/TPN 14D					
690r2	Nfil3	4.384648						
690r3	Nfil3	5.314424						
847r1	Nfil3	0.814401	CHOW					
847r2	Nfil3	0.853073						
847r3	Nfil3	1.019172						
848r1	Nfil3	1	CHOW					
848r2	Nfil3	1.125814						
848r3	Nfil3	1.412141						
849r1	Nfil3	1.091532	CHOW					
849r2	Nfil3	1.14064						
849r3	Nfil3	1.086717						
850r1	Nfil3	1.637664	CHOW					
850r2	Nfil3	2.049557						
850r3	Nfil3	1.834129						

Sample N	Target Na	RQ						
314r1	Dec1	0.557969	2.0%DSS	Dec1	CHOW	DSS-PN	ILKo Chow	IL1 DSS-PN
314r2	Dec1	0.446277			0.459123	0.488325	1.029082	2.308826
314r3	Dec1	0.460729						
317r1	Dec1	0.615722	2.0%DSS 4D/CHOW 14D					
317r2	Dec1	0.85005			1.030582	0.431162	1.276017	0.866399
317r3	Dec1	0.731632						
321r1	Dec1	1.247957	2.0%DSS 4D/CHOW 14D					
321r2	Dec1	1.487046			1.674076	#VALUE!	0.645447	2.667716
321r3	Dec1	1.426207						
339r1	Dec1	#VALUE!						
339r2	Dec1	#VALUE!			1.792307	1.390671		1.346579
339r3	Dec1	#VALUE!						
343r1	Dec1	#VALUE!						
343r2	Dec1	#VALUE!				0.623709		
343r3	Dec1	#VALUE!						
344r1	Dec1	#VALUE!						
344r2	Dec1	#VALUE!				0.513929		
344r3	Dec1	#VALUE!						
345r1	Dec1	#VALUE!						
345r2	Dec1	#VALUE!						
345r3	Dec1	#VALUE!						
349r1	Dec1	2.30693	(IL1-/-) 2.0%DSS/TPN14D					
349r2	Dec1	2.200543						
349r3	Dec1	2.419004						
351r1	Dec1	0.864984	(IL1-/-) 2.0%DSS/TPN14D					
351r2	Dec1	0.895167						
351r3	Dec1	0.839046						
352r1	Dec1	2.571299	(IL1-/-) 2.0%DSS/TPN14D					
352r2	Dec1	2.550768						
352r3	Dec1	2.881081						
353r1	Dec1	1.293303	(IL1-/-) 2.0%DSS/TPN14D					
353r2	Dec1	1.28964						
353r3	Dec1	1.456794						
364r1	Dec1	0.901866	(IL1-/-) CHOW 14D					
364r2	Dec1	1.047593						
364r3	Dec1	1.137787						
365r1	Dec1	1.177313	(IL1-/-) CHOW 14D					
365r2	Dec1	1.29332						
365r3	Dec1	1.357418						
545r1	Dec1	0.38379	2.0%DSS 4D/TPN 14D					
545r2	Dec1	0.392563						
545r3	Dec1	0.517134						
547r1	Dec1	#VALUE!						
547r2	Dec1	#VALUE!						
547r3	Dec1	#VALUE!						
568r1	Dec1	0.284465	2.0%DSS 4D/CHOW 14D					
568r2	Dec1	0.332764						
568r3	Dec1	0.329119						
571r1	Dec1	0.176097	2.0%DSS 4D/CHOW 14D					
571r2	Dec1	0.161061						
571r3	Dec1	0.132021						
665r1	Dec1	0.61401	(IL1-/-) CHOW 14D					
665r2	Dec1	0.632119						
665r3	Dec1	0.690213						
686r1	Dec1	#VALUE!	2.0%DSS 4D/TPN 14D					
686r2	Dec1	#VALUE!						
686r3	Dec1	#VALUE!						
688r1	Dec1	1.069766	2.0%DSS 4D/TPN 14D					
688r2	Dec1	1.455562						
688r3	Dec1	1.646684						
689r1	Dec1	0.64556	2.0%DSS 4D/TPN 14D					
689r2	Dec1	0.617935						
689r3	Dec1	0.607632						
690r1	Dec1	0.482	2.0%DSS 4D/TPN 14D					
690r2	Dec1	0.522219						
690r3	Dec1	0.537567						
847r1	Dec1	0.428712	CHOW					
847r2	Dec1	0.458526						
847r3	Dec1	0.49013						
848r1	Dec1	1	CHOW					
848r2	Dec1	1.083468						
848r3	Dec1	1.008277						
849r1	Dec1	1.5008	CHOW					
849r2	Dec1	1.680645						
849r3	Dec1	1.840783						
850r1	Dec1	1.687581	CHOW					
850r2	Dec1	1.668471						
850r3	Dec1	2.020869						

Sample N	Target Na	RQ						
314r1	Npas2	#VALUE!	2.0%DSS	Npas2	CHOW	DSS-PN	ILKo Chow	IL1 DSS-PN
314r2	Npas2	#VALUE!			0.588602	#VALUE!	1.688838	0.41587
314r3	Npas2	#VALUE!						
317r1	Npas2	0.234804	2.0%DSS 4D/CHOW 14D					
317r2	Npas2	0.183248			1.045745	0.636837	1.660238	0.516619
317r3	Npas2	0.435299						
321r1	Npas2	0.279704	2.0%DSS 4D/CHOW 14D					
321r2	Npas2	0.424014			0.734379	0.870169	0.785812	0.572813
321r3	Npas2	0.451096						
339r1	Npas2	0.305744						
339r2	Npas2	0.531093			0.418796	#VALUE!		0.429608
339r3	Npas2	0.511609						
343r1	Npas2	1.374751						
343r2	Npas2	1.363947				0.253648		
343r3	Npas2	1.606823						
344r1	Npas2	0.723072						
344r2	Npas2	0.730651				0.276546		
344r3	Npas2	0.935328						
345r1	Npas2	0.46137						
345r2	Npas2	0.628922						
345r3	Npas2	0.414605						
349r1	Npas2	0.404865	(IL1-/-) 2.0%DSS/TPN14D					
349r2	Npas2	0.235745						
349r3	Npas2	0.606999						

Sample N	Target Na	RQ						
314r1	Dec2	#VALUE!	2.0%DSS	Dec2	CHOW	DSS-PN	ILKo Chow	IL1 DSS-PN
314r2	Dec2	#VALUE!			0.722807	#VALUE!	98.00840624	239.6306
314r3	Dec2	#VALUE!						
317r1	Dec2	24.63138	2.0%DSS 4D/CHOW 14D					
317r2	Dec2	20.54508			1.840927	20.27114	27.65561415	105.582
317r3	Dec2	23.96961						
321r1	Dec2	179.9571	2.0%DSS 4D/CHOW 14D					
321r2	Dec2	199.9318			3.436663	#VALUE!	31.67385278	146.4364
321r3	Dec2	230.979						
339r1	Dec2	#VALUE!						
339r2	Dec2	#VALUE!			3.157655	57.97207		86.9243
339r3	Dec2	#VALUE!						
343r1	Dec2	#VALUE!						
343r2	Dec2	#VALUE!				255.426		
343r3	Dec2	#VALUE!						
344r1	Dec2	#VALUE!						
344r2	Dec2	#VALUE!				159.1985		
344r3	Dec2	#VALUE!						
345r1	Dec2	#VALUE!						
345r2	Dec2	#VALUE!				69.63439		
345r3	Dec2	#VALUE!						
349r1	Dec2	226.9617	(IL1-/-) 2.0%DSS/TPN14D					
349r2	Dec2	129.2568						
349r3	Dec2	362.6734						

Sample N	Target Na	RQ										
313r1	Nr1d1	1.363052	2.0%DSS	Nr1d1	Chow	DSS	PN	DSS-PN	IL1 Chow	IL1 DSS_I	TNF Chow	TNF DSS-PN
313r2	Nr1d1	1.343058			0.814005	2.408622	4.568178	1.424303284	1.850646	9.32389	0.277754	0.54147
313r3	Nr1d1	1.5668										
317r1	Nr1d1	2.658243	2.0%DSS 4D/CHOW 14D									
317r2	Nr1d1	2.023489			1.01958	0.075343	0.379186	0.208813029	0.852668	4.839841	0.949856	0.131811
317r3	Nr1d1	2.544134										
339r1	Nr1d1	4.138014	TPN 14D									
339r2	Nr1d1	4.94645			2.318226	0.340782	0.84973	1.352426715	0.390726	12.8359	0.424526	0.099796
339r3	Nr1d1	4.62007										
343r1	Nr1d1	0.371327	TPN 14D									
343r2	Nr1d1	0.454214			1.693251	1.394348	1.702966	2.514792652		6.504919	0.644937	0.16601
343r3	Nr1d1	0.312017										
344r1	Nr1d1	0.697626	TPN 14D									
344r2	Nr1d1	0.879219										
344r3	Nr1d1	0.972346		Nr1d1	Chow	DSS-PN	TNF Chow	TNF DSS-PN				
345r1	Nr1d1	1.439694	TPN 14D		0.814005	1.424303	0.277754	0.541470167				
345r2	Nr1d1	1.653187										
345r3	Nr1d1	2.016018										
349r1	Nr1d1	8.665404	(IL1-/-) 2.0%DSS/TPN		1.01958	0.208813	0.949856	0.131811061				
349r2	Nr1d1	10.04904										
349r3	Nr1d1	9.257222										
351r1	Nr1d1	4.449604	(IL1-/-) 2.0%DSS/TPN		2.318226	1.352427	0.424526	0.099795766				
351r2	Nr1d1	4.711714										
351r3	Nr1d1	5.358204										

Sample N	Target Na	RQ										
313r1	Rora	0.341968	2.0%DSS	Rora	Chow	DSS	PN	DSS-PN	IL1 Chow	IL1 DSS_I	TNF Chow	TNF DSS-PN
313r2	Rora	0.370518			0.888309469	0.829704	0.819087	0.36177	1.19388	1.244496	0.891387	0.877183
313r3	Rora	0.372824										
317r1	Rora	0.821459	2.0%DSS 4D/CHOW 14D									
317r2	Rora	0.877123			0.994071262	0.687663	1.369816	0.874661	1.097893	1.053639	0.912869	0.590344
317r3	Rora	0.79053										
339r1	Rora	0.823662	TPN 14D									
339r2	Rora	0.823263			1.123516591	0.629075	0.67914	0.700611	0.900556	1.16212	0.870823	0.519849
339r3	Rora	0.810337										
343r1	Rora	1.284256	TPN 14D									
343r2	Rora	1.343473			0.863195428	0.869008	0.977851	0.537238		0.960582	0.845112	0.650985
343r3	Rora	1.481719										
344r1	Rora	0.677708	TPN 14D									
344r2	Rora	0.671334										
344r3	Rora	0.688377		Rora	Chow	DSS-PN	IL1 Chow	IL1 DSS_PN				
345r1	Rora	1.108008	TPN 14D		0.888309469	0.36177	1.19388	1.244496				
345r2	Rora	0.909122										
345r3	Rora	0.916425										
349r1	Rora	1.173455	(IL1-/-) 2.0%DSS/TPN		0.994071262	0.874661	1.097893	1.053639				
349r2	Rora	1.306207										
349r3	Rora	1.253824										
351r1	Rora	1.00684	(IL1-/-) 2.0%DSS/TPN		1.123516591	0.700611	0.900556	1.16212				
351r2	Rora	1.054504										
351r3	Rora	1.099574										

351r1	Npas2	0.62503	(IL1-/-) 2.0%DSS/TPN14D
351r2	Npas2	0.382564	
351r3	Npas2	0.542263	
352r1	Npas2	0.590546	(IL1-/-) 2.0%DSS/TPN14D
352r2	Npas2	0.664088	
352r3	Npas2	0.463805	
353r1	Npas2	0.384666	(IL1-/-) 2.0%DSS/TPN14D
353r2	Npas2	0.473407	
353r3	Npas2	0.430751	
364r1	Npas2	1.728142	(IL1-/-) CHOW 14D
364r2	Npas2	1.570329	
364r3	Npas2	1.768043	
365r1	Npas2	1.674131	(IL1-/-) CHOW 14D
365r2	Npas2	1.504905	
365r3	Npas2	1.801677	
545r1	Npas2	0.639951	2.0%DSS 4D/TPN 14D
545r2	Npas2	0.724186	
545r3	Npas2	0.546375	
547r1	Npas2	#VALUE!	
547r2	Npas2	#VALUE!	
547r3	Npas2	#VALUE!	
568r1	Npas2	0.487907	2.0%DSS 4D/CHOW 14D
568r2	Npas2	0.729946	
568r3	Npas2	0.570558	
571r1	Npas2	0.396065	2.0%DSS 4D/CHOW 14D
571r2	Npas2	0.309918	
571r3	Npas2	0.391621	
665r1	Npas2	0.839098	(IL1-/-) CHOW 14D
665r2	Npas2	0.698602	
665r3	Npas2	0.819736	
686r1	Npas2	0.876238	2.0%DSS 4D/TPN 14D
686r2	Npas2	0.814368	
686r3	Npas2	0.919902	
688r1	Npas2	#VALUE!	2.0%DSS 4D/TPN 14D
688r2	Npas2	#VALUE!	
688r3	Npas2	#VALUE!	
689r1	Npas2	0.170084	2.0%DSS 4D/TPN 14D
689r2	Npas2	0.223185	
689r3	Npas2	0.367675	
690r1	Npas2	0.285014	2.0%DSS 4D/TPN 14D
690r2	Npas2	0.217957	
690r3	Npas2	0.326668	
847r1	Npas2	0.562299	CHOW
847r2	Npas2	0.569417	
847r3	Npas2	0.634091	
848r1	Npas2	1	CHOW
848r2	Npas2	0.993202	
848r3	Npas2	1.144034	
849r1	Npas2	0.726431	CHOW
849r2	Npas2	0.776279	
849r3	Npas2	0.700428	
850r1	Npas2	0.388622	CHOW
850r2	Npas2	0.426511	
850r3	Npas2	0.441255	

351r1	Dec2	96.94902	(IL1-/-) 2.0%DSS/TPN14D
351r2	Dec2	76.3003	
351r3	Dec2	143.4966	
352r1	Dec2	113.1612	(IL1-/-) 2.0%DSS/TPN14D
352r2	Dec2	115.3207	
352r3	Dec2	210.8272	
353r1	Dec2	94.64723	(IL1-/-) 2.0%DSS/TPN14D
353r2	Dec2	64.16593	
353r3	Dec2	101.9598	
364r1	Dec2	85.5236	(IL1-/-) CHOW 14D
364r2	Dec2	116.5052	
364r3	Dec2	91.99645	
365r1	Dec2	35.14138	(IL1-/-) CHOW 14D
365r2	Dec2	27.1743	
365r3	Dec2	20.65116	
545r1	Dec2	14.94599	2.0%DSS 4D/TPN 14D
545r2	Dec2	27.92979	
545r3	Dec2	17.93763	
547r1	Dec2	68.60674	2.0%DSS 4D/TPN 14D
547r2	Dec2	60.01605	
547r3	Dec2	80.28037	
568r1	Dec2	16.48541	2.0%DSS 4D/CHOW 14D
568r2	Dec2	24.35433	
568r3	Dec2	27.58147	
571r1	Dec2	26.50009	2.0%DSS 4D/CHOW 14D
571r2	Dec2	19.5692	
571r3	Dec2	24.38046	
665r1	Dec2	34.13952	(IL1-/-) CHOW 14D
665r2	Dec2	33.7295	
665r3	Dec2	27.15254	
686r1	Dec2	#VALUE!	2.0%DSS 4D/TPN 14D
686r2	Dec2	#VALUE!	
686r3	Dec2	#VALUE!	
688r1	Dec2	30.65596	2.0%DSS 4D/TPN 14D
688r2	Dec2	82.33852	
688r3	Dec2	60.92173	
689r1	Dec2	249.0393	2.0%DSS 4D/TPN 14D
689r2	Dec2	258.0771	
689r3	Dec2	259.1616	
690r1	Dec2	141.5016	2.0%DSS 4D/TPN 14D
690r2	Dec2	154.3267	
690r3	Dec2	181.7671	
847r1	Dec2	0.769134	CHOW
847r2	Dec2	0.906959	
847r3	Dec2	0.492329	
848r1	Dec2	1	CHOW
848r2	Dec2	2.504165	
848r3	Dec2	2.018617	
849r1	Dec2	2.579978	CHOW
849r2	Dec2	4.450724	
849r3	Dec2	3.279289	
850r1	Dec2	2.011881	CHOW
850r2	Dec2	3.961832	
850r3	Dec2	3.499252	

352r1	Nr1d1	12.86556	(IL1-/-) 2.0%DSS/TPN	1.693251	2.514793	0.644937		0.166010006
352r2	Nr1d1	12.88807						
352r3	Nr1d1	12.75405						
353r1	Nr1d1	6.986104	(IL1-/-) 2.0%DSS/TPN	Chow	DSS-PN	IL1 Chow	IL1 DSS_PN	
353r2	Nr1d1	5.62509		0.814005	1.424303	1.850646		9.323889752
353r3	Nr1d1	6.903563						
364r1	Nr1d1	1.521257	(IL1-/-) CHOW 14D					
364r2	Nr1d1	1.820845		1.01958	0.208813	0.852668		4.839840829
364r3	Nr1d1	2.209836						
365r1	Nr1d1	0.763009	(IL1-/-) CHOW 14D					
365r2	Nr1d1	0.768575		2.318226	1.352427	0.390726		12.83589745
365r3	Nr1d1	1.02642						
545r1	Nr1d1	0.219714	2.0%DSS 4D/TPN 14D					
545r2	Nr1d1	0.195053		1.693251	2.514793			6.504918982
545r3	Nr1d1	0.211672						
571r1	Nr1d1	0.114311	2.0%DSS 4D/CHOW 14D					
571r2	Nr1d1	0.065586						
571r3	Nr1d1	0.046131						
587r1	Nr1d1	0.501155	TNF(-/-) 2.0%DSS(4D)/TPN 14D					
587r2	Nr1d1	0.731737						
587r3	Nr1d1	0.391518						
604r1	Nr1d1	0.14857	TNF(-/-) 2.0%DSS(4D)/TPN 14D					
604r2	Nr1d1	0.157738						
604r3	Nr1d1	0.089125						
605r1	Nr1d1	0.081692	TNF(-/-) 2.0%DSS(4D)/TPN 14D					
605r2	Nr1d1	0.110647						
605r3	Nr1d1	0.107048						
606r1	Nr1d1	0.15769	TNF(-/-) 2.0%DSS(4D)/TPN 14D					
606r2	Nr1d1	0.173248						
606r3	Nr1d1	0.167091						
616r1	Nr1d1	0.213677	TNF(-/-)/CHOW 14D					
616r2	Nr1d1	0.268732						
616r3	Nr1d1	0.350853						
618r1	Nr1d1	1.074504	TNF(-/-)/CHOW 14D					
618r2	Nr1d1	0.80287						
618r3	Nr1d1	0.972194						
619r1	Nr1d1	0.438617	TNF(-/-)/CHOW 14D					
619r2	Nr1d1	0.31498						
619r3	Nr1d1	0.51998						
620r1	Nr1d1	0.762148	TNF(-/-)/CHOW 14D					
620r2	Nr1d1	0.604118						
620r3	Nr1d1	0.568544						
665r1	Nr1d1	0.451075	(IL1-/-) CHOW 14D					
665r2	Nr1d1	0.327527						
665r3	Nr1d1	0.393577						
689r1	Nr1d1	1.40779	2.0%DSS 4D/TPN 14D					
689r2	Nr1d1	1.095038						
689r3	Nr1d1	1.554452						
690r1	Nr1d1	2.149811	2.0%DSS 4D/TPN 14D					
690r2	Nr1d1	2.753189						
690r3	Nr1d1	2.641378						
699r1	Nr1d1	0.279533	2.0%DSS 4D/CHOW 14D					
699r2	Nr1d1	0.428857						
699r3	Nr1d1	0.313956						
768r1	Nr1d1	1.278468	2.0%DSS 4D/CHOW 14D					
768r2	Nr1d1	1.503441						
768r3	Nr1d1	1.401134						
847r1	Nr1d1	0.770677	CHOW					
847r2	Nr1d1	0.87196						
847r3	Nr1d1	0.79938						
848r1	Nr1d1	1	CHOW					
848r2	Nr1d1	1.015948						
848r3	Nr1d1	1.042792						
849r1	Nr1d1	2.20317	CHOW					
849r2	Nr1d1	2.166209						
849r3	Nr1d1	2.585299						
850r1	Nr1d1	1.5087	CHOW					
850r2	Nr1d1	1.669473						
850r3	Nr1d1	1.90158						

352r1	Rora	1.078612	(IL1-/-) 2.0%DSS/TPN			0.863195428	0.537238		0.960582
352r2	Rora	1.220258							
352r3	Rora	1.18749							
353r1	Rora	0.85112	(IL1-/-) 2.0	Rora	Chow		DSS-PN	TNF Chow	TNF DSS-PN
353r2	Rora	1.015898				0.888309469	0.36177	0.891387	0.877183
353r3	Rora	1.014728							
364r1	Rora	1.060762	(IL1-/-) CHOW 14D						
364r2	Rora	1.23446				0.994071262	0.874661	0.912869	0.590344
364r3	Rora	1.286419							
365r1	Rora	1.022725	(IL1-/-) CHOW 14D						
365r2	Rora	1.048148				1.123516591	0.700611	0.870823	0.519849
365r3	Rora	1.222805							
545r1	Rora	0.812627	2.0%DSS 4D/TPN 14D						
545r2	Rora	0.809393				0.863195428	0.537238	0.845112	0.650985
545r3	Rora	1.001964							
571r1	Rora	0.743285	2.0%DSS 4D/CHOW 14D						
571r2	Rora	0.607069							
571r3	Rora	0.712635							
587r1	Rora	0.960294	TNF(-/-) 2.0%DSS(4D)/TPN 14D						
587r2	Rora	0.83759							
587r3	Rora	0.833664							
604r1	Rora	0.529632	TNF(-/-) 2.0%DSS(4D)/TPN 14D						
604r2	Rora	0.593691							
604r3	Rora	0.647707							
605r1	Rora	0.518629	TNF(-/-) 2.0%DSS(4D)/TPN 14D						
605r2	Rora	0.4578							
605r3	Rora	0.583118							
606r1	Rora	0.644848	TNF(-/-) 2.0%DSS(4D)/TPN 14D						
606r2	Rora	0.613676							
606r3	Rora	0.694431							
616r1	Rora	0.878643	TNF(-/-)/CHOW 14D						
616r2	Rora	0.912662							
616r3	Rora	0.882854							
618r1	Rora	0.853052	TNF(-/-)/CHOW 14D						
618r2	Rora	0.920645							
618r3	Rora	0.964909							
619r1	Rora	0.863229	TNF(-/-)/CHOW 14D						
619r2	Rora	0.818842							
619r3	Rora	0.930399							
620r1	Rora	0.796847	TNF(-/-)/CHOW 14D						
620r2	Rora	0.78928							
620r3	Rora	0.949208							
665r1	Rora	0.886387	(IL1-/-) CHOW 14D						
665r2	Rora	0.906154							
665r3	Rora	0.909126							
689r1	Rora	0.641879	2.0%DSS 4D/TPN 14D						
689r2	Rora	0.732862							
689r3	Rora	0.727092							
690r1	Rora	0.443303	2.0%DSS 4D/TPN 14D						
690r2	Rora	0.577324							
690r3	Rora	0.591085							
699r1	Rora	0.616546	2.0%DSS 4D/CHOW 14D						
699r2	Rora	0.664526							
699r3	Rora	0.606152							
768r1	Rora	0.863006	2.0%DSS 4D/CHOW 14D						
768r2	Rora	0.861418							
768r3	Rora	0.882601							
847r1	Rora	0.905054	CHOW						
847r2	Rora	0.866597							
847r3	Rora	0.893277							
848r1	Rora	1	CHOW						
848r2	Rora	0.974007							
848r3	Rora	1.008207							
849r1	Rora	1.029377	CHOW						
849r2	Rora	1.142498							
849r3	Rora	1.198675							
850r1	Rora	0.872626	CHOW						
850r2	Rora	0.826313							
850r3	Rora	0.890647							

Sample Name	Target Name	RQ		CHOW	DSS	PN	DSS-PN	TNF CHOW	TNF DSS-PN
313r1	Clock	0.22518	2.0%DSS 4D/TPN 14D	CHOW	DSS	PN	DSS-PN	TNF CHC	TNF DSS-PN
313r2	Clock	0.22745		0.8653	0.65157	1.08304	0.23206	0.53054	1.1314
313r3	Clock	0.24356							
317r1	Clock	0.61903	2.0%DSS 4D/CHOW 14D						
317r2	Clock	0.66174		0.98208	1.07994	1.50691	1.10146	0.45239	0.45214
317r3	Clock	0.67393							
321r1	Clock	0.99197	2.0%DSS 4D/CHOW 14D						
321r2	Clock	1.11106		0.79668	0.60966	1.10107	0.70105	0.60038	0.4996
321r3	Clock	1.1388							
339r1	Clock	1.00527	TPN 14D						
339r2	Clock	1.11869		0.63114	0.4984	0.7815	0.86898	0.57994	0.44391
339r3	Clock	1.12517							
343r1	Clock	1.28044	TPN 14D						
343r2	Clock	1.60148							
343r3	Clock	1.63885							
344r1	Clock	1.04779	TPN 14D						
344r2	Clock	1.09317							
344r3	Clock	1.16224							
345r1	Clock	0.72572	TPN 14D						
345r2	Clock	0.82296							
345r3	Clock	0.79584							
568r1	Clock	0.57733	2.0%DSS 4D/CHOW 14D						
568r2	Clock	0.6098							
568r3	Clock	0.64198							
571r1	Clock	0.47052	2.0%DSS 4D/CHOW 14D						
571r2	Clock	0.52627							
571r3	Clock	0.47067							
587r1	Clock	1.08449	TNF(-/-) 2.0%DSS(4D)/TPN 14D						
587r2	Clock	1.06570							
587r3	Clock	1.24391							
604r1	Clock	0.42612	TNF(-/-) 2.0%DSS(4D)/TPN 14D						
604r2	Clock	0.48235							
604r3	Clock	0.44794							
605r1	Clock	0.50200	TNF(-/-) 2.0% DSS(4D)/TPN 14D						
605r2	Clock	0.56122							
605r3	Clock	0.43549							
606r1	Clock	0.44476	TNF(-/-) 2.0%DSS(4D)/TPN 14D						
606r2	Clock	0.41135							
606r3	Clock	0.47582							
616r1	Clock	0.53499	TNF(-/-)/CHOW 14D						
616r2	Clock	0.47035							
616r3	Clock	0.58627							
618r1	Clock	0.46717	TNF(-/-)/CHOW 14D						
618r2	Clock	0.47458							
618r3	Clock	0.41546							
619r1	Clock	0.5666	TNF(-/-)/CHOW 14D						
619r2	Clock	0.63814							
619r3	Clock	0.59639							
620r1	Clock	0.55007	TNF(-/-)/CHOW 14D						
620r2	Clock	0.58927							
620r3	Clock	0.60047							
688r1	Clock	1.06435	2.0%DSS 4D/TPN 14D						
688r2	Clock	1.0142							
688r3	Clock	1.22584							
689r1	Clock	0.64261	2.0%DSS 4D/TPN 14D						
689r2	Clock	0.69937							
689r3	Clock	0.76119							
690r1	Clock	0.79319	2.0%DSS 4D/TPN 14D						
690r2	Clock	0.88518							
690r3	Clock	0.92856							
847r1	Clock	0.74787	CHOW						
847r2	Clock	0.92327							
847r3	Clock	0.92477							
848r1	Clock	1	CHOW						
848r2	Clock	1.01635							
848r3	Clock	0.92989							
849r1	Clock	0.72524	CHOW						
849r2	Clock	0.75917							
849r3	Clock	0.90582							
850r1	Clock	0.65011	CHOW						
850r2	Clock	0.61634							
850r3	Clock	0.62697							

Sample N	Target Na	RQ										
313r1	Aec1/Pu	3.62153	2.0%DSS	Aec1/Pucard	Chow	DSS	PN	DSS-PN	IL1 Chow	IL1 DSS	TNF Chw	TNF DSS-PN
313r2	Aec1/Pu	4.5612			1.10866	#DIV/0!	#DIV/0!	4.3308	8.13307	6.52159	2.09465	28.8688
313r3	Aec1/Pu	4.80967										
			2.0% DSS 4D/CHOW 14D									
					1.63711	#DIV/0!	#DIV/0!	5.60675	3.76428	15.4183	3.0697	7.85315
			TPN 14D									
					1.31267	#DIV/0!	#DIV/0!	10.0325	2.7871	5.09686	2.4073	11.8132
			TPN 14D									
					1.76976	#DIV/0!	#DIV/0!	6.83663		27.4367	3.06466	3.89693
			TPN 14D									
				Aec1/Pucard	Chow	DSS-PN	IL1 Chow	IL1 DSS-PN				
			TPN 14D		1.10866	4.3308	8.13307	6.52159				
349r1	Aec1/Pu	6.54178	(IL1-/-) 2.0% DSS/TPN14D		1.63711	5.60675	3.76428	15.4183				
349r2	Aec1/Pu	5.97899										
349r3	Aec1/Pu	7.04399										
351r1	Aec1/Pu	14.8522	(IL1-/-) 2.0%DSS/TPN14D		1.31267	10.0325	2.7871	5.09686				
351r2	Aec1/Pu	14.8134										
351r3	Aec1/Pu	18.5802										
352r1	Aec1/Pu	4.63617	(IL1-/-) 2.0%DSS/TPN14D		1.76976	6.83663		27.4367				
352r2	Aec1/Pu	5.73433										
352r3	Aec1/Pu	4.92009										
353r1	Aec1/Pu	24.5171	(IL1-/-) 2.	Aec1/Pucard	Chow	DSS-PN	TNF Chw	TNF DSS-PN				
353r2	Aec1/Pu	28.8404			1.10866	4.3308	2.09465	28.8688				
353r3	Aec1/Pu	30.9527										
364r1	Aec1/Pu	8.03803	(IL1-/-) CHOW 14D									
364r2	Aec1/Pu	8.42107			1.63711	5.60675	0.912868	7.85315				
364r3	Aec1/Pu	7.9401										
365r1	Aec1/Pu	4.45609	(IL1-/-) CHOW 14D									
365r2	Aec1/Pu	3.34586			1.31267	10.0325	2.4073	11.8132				
365r3	Aec1/Pu	3.49089										
			2.0%DSS 4D/TPN 14D									
					1.76976	6.83663	3.06466	3.89693				

[illegible]
